# Leucine‐Rich Repeat‐Containing G Protein‐Coupled Receptor 6 Ameliorates Pressure Overload‐Induced Cardiac Hypertrophy by Regulating Cardiomyocyte Metabolic Reprogramming

**DOI:** 10.1002/advs.202417597

**Published:** 2025-04-11

**Authors:** Mengmeng Zhao, Jianfang Liu, Shanshan Peng, Zihui Zheng, Siqi Liu, Jun Wan, Yao Xu, Menglong Wang

**Affiliations:** ^1^ Department of Cardiology Renmin Hospital of Wuhan University Wuhan 430060 China; ^2^ Cardiovascular Research Institute Wuhan University Wuhan 430060 China; ^3^ Hubei Key Laboratory of Cardiology Wuhan 430060 China

**Keywords:** cardiac hypertrophy, Lgr6, metabolic reprogramming, PPARα, USP4

## Abstract

Metabolic reprogramming is a pivotal mechanism in the pathogenesis of pathological cardiac hypertrophy. Leucine‐rich repeat‐containing G protein‐coupled receptor 6 (Lgr6) has emerged as a significant player in cardiovascular diseases. In this study, the potential of Lgr6 to counteract pressure overload (PO)‐induced cardiac hypertrophy is investigated, and the underlying mechanisms involved are elucidated. Transverse aortic constriction (TAC) is induced to establish an in vivo cardiac hypertrophy model. Adeno‐associated virus 9 and adenovirus vectors are utilized to knock down and overexpress Lgr6 in cardiomyocytes, respectively. The effects of Lgr6 and its downstream molecules are subsequently determined using RNA sequencing and chromatin immunoprecipitation. Significant downregulation of Lgr6 expression is observed in the heart after TAC and in cardiomyocytes treated with phenylephrine. Lgr6 deficiency accelerated and Lgr6 overexpression inhibits cardiac hypertrophy and dysfunction after TAC. Mechanistically, the in vivo and in vitro experiments suggest that Lgr6 regulates the expression of ubiquitin specific protease 4 (USP4) and peroxisome proliferator‐activated receptor alpha (PPARα) by activating the cGMP/PKG/CREB1 signalling pathway, thereby regulating cardiomyocyte metabolic reprogramming after PO. Targeting Lgr6 can be a potential therapeutic strategy to treat pathological cardiac hypertrophy.

## Graphical abstract

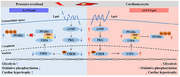

## 1 Introduction

Cardiac hypertrophy is a compensatory response of the myocardium to prolonged pressure or volume overload and is characterized primarily by increases in cardiomyocyte volume and extracellular matrix formation.^[^ ^1^ ^]^ Persistent pressure or volume overload can lead to decompensated cardiac hypertrophy, which can progress to end‐stage conditions such as dilated cardiomyopathy and heart failure.^[^ ^2^ ^]^ Research indicates that cardiac hypertrophy is an independent risk factor for heart failure and increases the risk of malignant arrhythmias, myocardial ischaemia, sudden death, and other conditions.^[^ ^3^ ^]^ However, the mechanisms underlying the onset and progression of cardiac hypertrophy remain incompletely understood, and there is a need for new, effective drugs to inhibit this process. Therefore, further elucidating the mechanisms of cardiac hypertrophy and identifying new drug targets for its prevention and treatment are of significant theoretical and clinical importance.

Analyses have demonstrated a reduction in fatty acid metabolism in myocardial samples from patients with end‐stage heart failure compared with normal myocardium samples, with a concomitant increase in the utilization of glucose and ketone bodies.^[^ ^4^ ^]^ This shift in metabolic substrate preference from fatty acids to glucose and ketone bodies is termed cardiomyocyte metabolic reprogramming.^[^ ^5^ ^]^ Interventions that inhibit glucose and ketone body utilization or enhance fatty acid oxidation have been shown to mitigate pathological cardiac hypertrophy.^[^ ^6^ ^]^ Studies indicate that promoting glycolysis or inhibiting fatty acid oxidation through various pathways can exacerbate pressure overload (PO)‐induced cardiac hypertrophy, whereas inhibiting glycolysis or promoting fatty acid oxidation can ameliorate it.^[^ ^7^ ^]^ For example, the upregulation of mitochondrial ATPase inhibitory factor 1 expression mediates an increase in glycolysis in the mouse heart, whereas the cardiac‐specific deletion of ATPIF1 in mice prevents metabolic reprogramming and pathological remodeling during chronic stress.^[^ ^8^ ^]^ Similarly, cardiac‐specific deletion of acetyl‐CoA carboxylase 2 enhances fatty acid oxidation, thereby ameliorating PO‐induced cardiac hypertrophy.^[^ ^9^ ^]^ Additionally, cardiac‐specific knockdown of phosphofructokinase‐1 or overexpression of long‐chain acyl‐CoA dehydrogenase mitigates PO‐induced cardiac hypertrophy and metabolic remodeling.^[^ ^6^ ^]^ The demethylase Jumonji C domain‐containing protein 4 regulates PO‐induced metabolic reprogramming and cardiac hypertrophy by degrading pyruvate kinase 2.^[^ ^10^ ^]^ These findings underscore the critical role of metabolic reprogramming in the progression of cardiac hypertrophy. Identifying key molecules that regulate cardiomyocyte metabolic reprogramming is therefore of paramount importance for developing interventions for cardiac hypertrophy.

G protein‐coupled receptors (GPCRs), also known as seven‐transmembrane receptors, constitute the largest family of transmembrane proteins in mammals and mediate a wide array of physiological processes.^[^ ^11^ ^]^ Members of the leucine‐rich repeat containing G protein‐coupled receptor (Lgr) family, a subgroup within the GPCR family, are highly conserved proteins with numerous leucine‐rich repeats, which are crucial for ligand binding, in their extracellular domains.^[^ ^12^ ^]^ The Lgr family comprises seven members: Lgr1 (follicle‐stimulating hormone receptor), Lgr2 (luteinizing hormone receptor), Lgr3 (thyroid‐stimulating hormone receptor), Lgr4‐6, Lgr7 (Relaxin family peptide receptor 1), and Lgr8 (Relaxin family peptide receptor 2).^[^ ^13^ ^]^ Previous studies have uncovered the role of Lgr family members in development and growth.^[^ ^13^ ^]^ However, recent studies have highlighted the regulatory roles of Lgrs in energy metabolism. For example, Lgr1 activates brown adipose tissue and enhances thermogenesis by increasing mitochondrial density.^[^ ^14^ ^]^ Tissue‐specific knockout of Lgr3 in white adipose tissue leads to increased adipocyte size and reduced thyrotropin‐induced lipolysis.^[^ ^13^ ^]^ Lgr4 is involved in mitigating cardiac ischaemia‒reperfusion injury by regulating extracellular signal‐regulated kinase activation to restore mitochondrial functional homeostasis.^[^ ^15^ ^]^ Lgr6 has been implicated in various cardiovascular diseases, including pulmonary hypertension and abdominal aortic aneurysm.^[^ ^16^ ^]^ Collectively, these findings suggest that members of the Lgr family may serve as critical targets for regulating energy metabolism in cardiovascular and metabolic diseases.

In this study, we observed that Lgr6 was downregulated in the myocardial tissues of PO model mice and patients with hypertrophic cardiomyopathy. Compared with PBS, phenylephrine (PE) reduced Lgr6 expression in mouse, rat, and human cardiomyocytes in vitro. Cardiomyocyte‐specific deficiency of Lgr6 exacerbated PO‐induced cardiac hypertrophy and dysfunction, whereas Lgr6 overexpression mitigated these effects. Mechanistically, Lgr6 reversed the PO‐induced reprogramming of cardiomyocyte metabolism by regulating the ubiquitin specific protease 4 (USP4)/Peroxisome proliferator‐activated receptor alpha (PPARα) signaling pathway. Overexpression of PPARα and USP4 ameliorated PO‐induced cardiac hypertrophy in Lgr6‐deficient mice. Collectively, these findings suggest that Lgr6 mediates metabolic reprogramming via the USP4/PPARα pathway, mitigating PO‐induced cardiac dysfunction and hypertrophy.

## 2 Results

### 2.1 Lgr6 is Downregulated During Cardiac Hypertrophy

**Figure 1:**
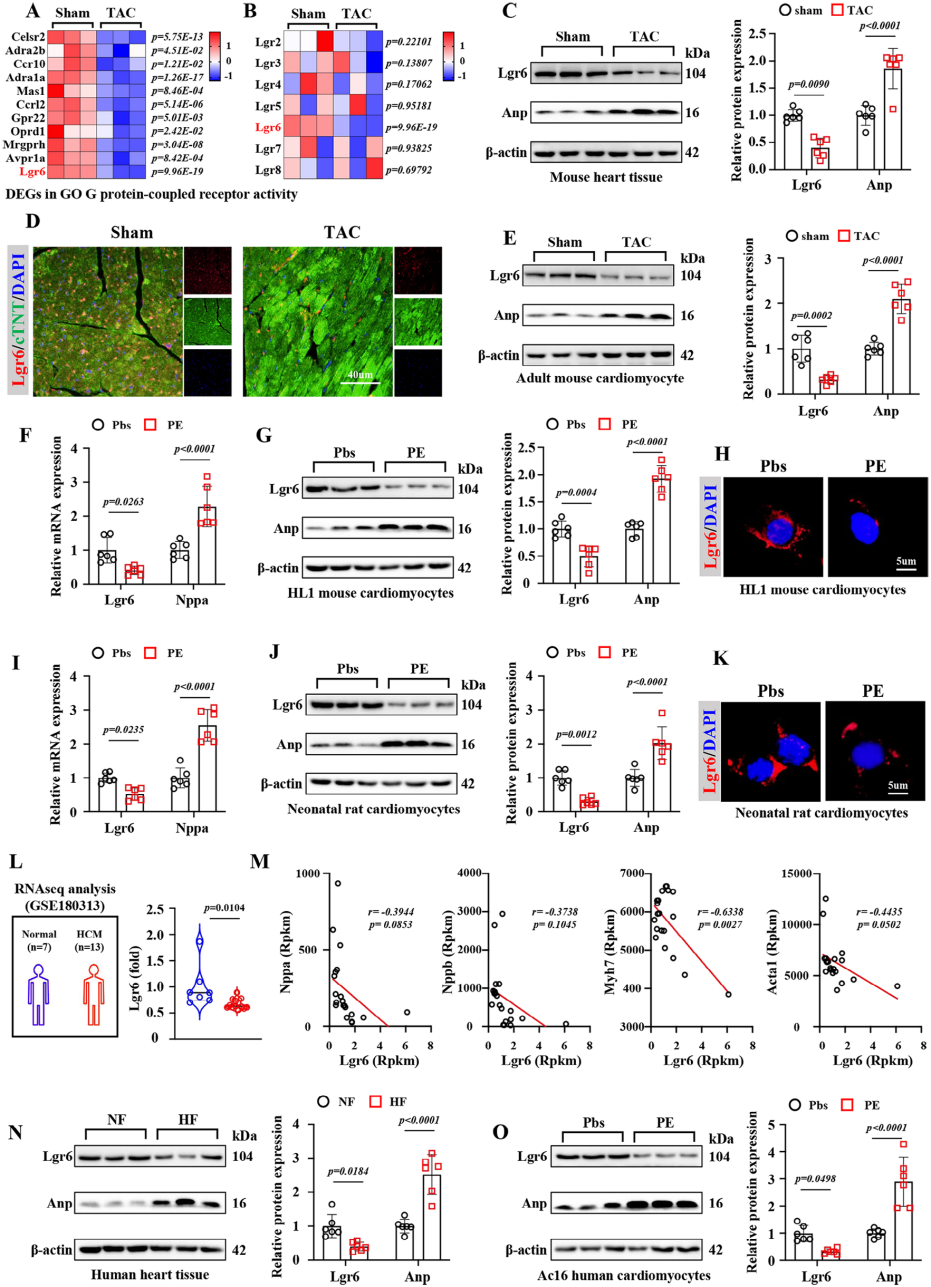
Lgr6 is downregulated during cardiac hypertrophy. A) Heatmap showing the cardiac expression of GPCRs in sham and TAC mice (n = 3). B) Heatmap showing the cardiac expression of Lgrs in sham and TAC mice (n = 3). C) Representative immunoblots and corresponding quantification showing cardiac Lgr6 and Anp in sham and TAC mice (n = 6). D) Representative immunofluorescence staining images of cardiac tissues for troponin and Lgr6 (n = 6). E) Representative immunoblots and corresponding quantification showing Lgr6 and Anp expression in adult mice cardiomyocytes from sham and TAC mice (n = 6). F) mRNA levels of Lgr6 and Nppa in HL1 mouse cardiomyocytes (n = 6). G) Representative immunoblots and corresponding quantification showing Lgr6 and Anp in HL1s (n = 6). H) Representative immunofluorescence staining images for Lgr6 in HL1s (n = 6). I,J) mRNA and protein levels of Lgr6 and Anp in NRCMs (n = 6). K) Representative immunofluorescence staining images for Lgr6 in NRCMs (n = 6). L) Cardiac expression of Lgr6 in normal and hypertrophic cardiomyopathy patients from GSE180313 datasets. M) Pearson correlation coefficient between the Lgr6 expression and biomarkers of cardiac hypertrophy, including Nppa, Nppb, Myh7 and Acta1, in GSE180313 datasets. N) Representative immunoblots and corresponding quantification showing Lgr6 and Anp expression in patients with heart failure and healthy donors (n = 6). O) Representative immunoblots and corresponding quantification showing Lgr6 and Anp expression in AC16 human cardiomyocytes (n = 6). All data are presented as mean ± standard error mean. Significance was assessed by two‐tailed unpaired Student's t‐test. Lgr6, leucine rich repeat containing G protein‐coupled receptor 6; Anp, atrial natriuretic peptide; Bnp, brain natriuretic peptide; TAC, transverse aortic constriction; PE, phenylephrine; NRCMs, neonatal rat cardiomyocytes; HCM, hypertrophic cardiomyopathy.

To identify potential regulators of cardiac hypertrophy, we first analyzed the gene expression profiles of left ventricular tissues from sham mice and PO model mice using RNA sequencing (RNAseq) (Figure S1A, Supporting Information). Gene set enrichment analysis (GSEA) analysis results suggested that Gene ontology G protein‐coupled receptor activity pathway was significantly downregulated (Figure S1B, Supporting Information). GPCRs have been reported to play an important role in cardiac hypertrophy.^[^ ^11^ ^]^ To investigate genes with functional significance in the G protein‐coupled receptor activity pathway, we further analyzed the differentially expressed genes (DEGs) in the G protein‐coupled receptor activity pathway and identified 11 downregulated genes, among which the downregulation of Lgr6 was most prominent in PO hearts (**Figure** **1**A). We then evaluated the expression of other members of the Lgr family and found that only Lgr6 was significantly downregulated in PO hearts (Figure 1B). Compared with sham hearts, Lgr6 expression was decreased in PO hearts, while Anp expression was upregulated (Figure 1C). To clarify the expression profile of Lgr6, online single‐cell dataset (GSE271946) of left ventricular tissues from sham and TAC mice was analyzed (Figure S1C, Supporting Information). Lgr6 was found to be widely expressed in the heart, with the highest expression in cardiomyocytes (Figure S1C, Supporting Information). We further compared the expression levels of Lgr6 in different cells in the hearts of sham and TAC mice and found that only the expression of Lgr6 in cardiomyocytes was significantly reduced in TAC mice (Figure S1D, Supporting Information). Reduced Lgr6 expression in cardiomyocytes under PO conditions was confirmed by immunofluorescence staining and immunoblotting (Figure 1D‐E). In vitro, PE effectively induced hypertrophy of HL‐1 mouse cardiomyocytes and neonatal rat cardiomyocytes (NRCMs), as shown by upregulated Anp mRNA and protein expression (Figure 1F–K). However, compared with PBS, PE significantly inhibited the expression of Lgr6 in HL‐1 cardiomyocytes and NRCMs (Figure 1F–K). Moreover, mechanical stretching experiments were employed to simulate pressure overload in vitro. Compared to static conditions, cyclic stretching inhibited the expression of Lgr6 in NRCMs (Figure S2A,B, Supporting Information).

To investigate the role of Lgr6 in the progression of normal heart to cardiac hypertrophy, we further analyzed the RNAseq data of left ventricular tissues from patients with hypertrophic cardiomyopathy (HCM) and healthy controls (Figure 1L). Compared with healthy controls, the expression of Lgr6 in left ventricular tissue of patients with HCM is significantly reduced (Figure 1L). Notably, the expression of Lgr6 is significantly negatively correlated with that of myosin heavy chain 7 (Myh7) (Figure 1M). Although Lgr6 also exhibits a negative correlation trend with the expression of natriuretic peptide precursor a (Nppa), natriuretic peptide precursor b (Nppb), and actin alpha 1, these correlations are not statistically significant. These results suggest that Lgr6 may be involved in the progression of cardiac hypertrophy (Figure 1M). In addition, compared with non‐heart failure patients, the expression of Lgr6 in the left ventricular tissue of heart failure patients caused by dilated cardiomyopathy is downregulated (Figure 1N). We then treated AC16 human cardiomyocytes with PE and found that compared to PBS, PE significantly inhibited the expression of Lgr6 in AC16 human cardiomyocytes (Figure 1O). These data indicate that downregulation of Lgr6 occurs in cardiomyocytes of PO mice.

### 2.2 Lgr6 Deficiency Accelerates PO‐Induced Cardiac Hypertrophy in Mice

To validate the role of Lgr6 in cardiac hypertrophy, we generated adeno‐associated virus 9 (AAV9) carrying the cTnT promoter to selectively knockdown Lgr6 expression in mouse cardiomyocytes (AAV9‐cTnT‐shLgr6 and AAV9‐cTnT‐null) (Figure S3A, Supporting Information). AAV9‐shLgr6 treatment effectively suppressed Lgr6 expression in the cardiomyocytes (Figure S3B,C, Supporting Information). Compared with AAV9‐null, four weeks of AAV9‐shLgr6 treatment had no significance on the cardiac function in sham mice (Figure S3D, Supporting Information). AAV9‐shLgr6 treatment did not increase the heart volume, the ratio of heart weight (HW) to body weight (BW), and the expression of Nppa and Nppb in sham mice (Figure S3E–G, Supporting Information).

**Figure 2:**
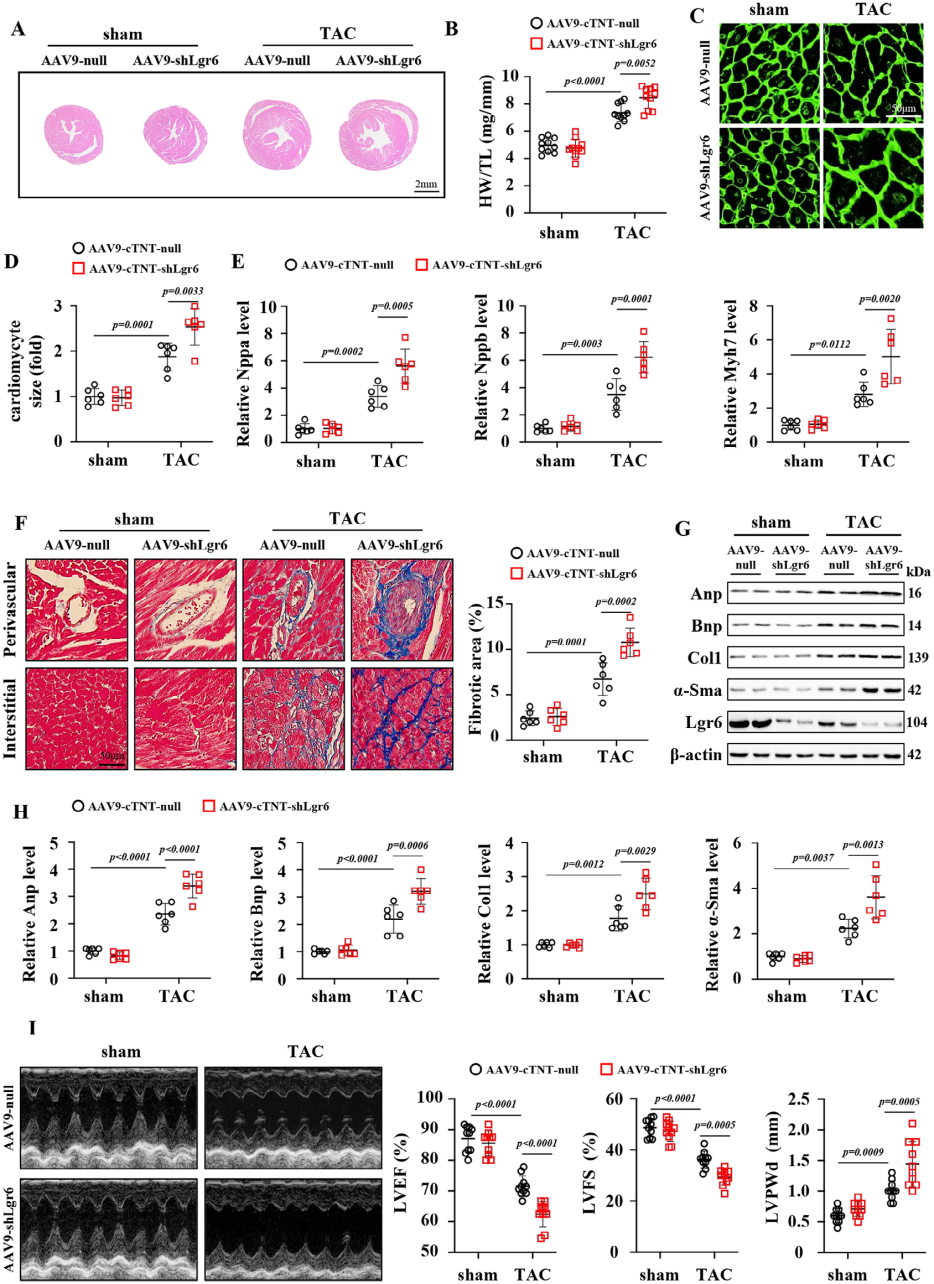
Lgr6 deficiency accelerates PO‐induced cardiac hypertrophy in mice. A) Representative images of H&E‐stained sections (n = 6). B) Heart weight (mg) and tibia length (mm) ratio (HW/TL) (n = 10). C,D) Representative images of WGA‐stained sections, and quantification of the cardiomyocyte cross‐sectional area based on WGA staining (n = 6). E) Real‐time quantitative reverse transcription polymerase chain reaction analysis comparing Nppa, Nppb and Myh7 mRNA expression in sham and TAC hearts (n = 6). F) Representative cardiac Masson trichrome staining in the peripheral (top) and interstitial (bottom) areas and quantification of the LV collagen volume (n = 6). G,H) Representative immunoblots and corresponding quantification showing cardiac protein expression of Anp, Bnp, Col1 and α‐Sma in sham and TAC mice (n = 6). I) Representative M‐mode echocardiographic images of the left ventricle and corresponding parameters showing cardiac function (n = 10). Significance was assessed by 2‐way ANOVA and Tukey's post hoc test. The data are shown as the mean±SD. Anp, atrial natriuretic peptide; Bnp, brain natriuretic peptide; Col1, collagen 1; Myh7, myosin heavy chain 7; α‐Sma, α‐smooth muscle actin; EF, ejection fraction; FS, fractional shortening; LVPWd, left ventricular posterior wall thickness at end‐diastole.

Compared to AAV9‐null, PO mice with Lgr6 deficiency exhibited increased heart volume and higher ratios of HW to tibia length (TL) (**Figure** **2**A,B). The cardiomyocyte size of PO mice with Lgr6 deficiency was significantly larger, as evidenced by WGA staining (Figure 2C,D). Additionally, mice with Lgr6 deficiency showed upregulated expression levels of cardiac hypertrophy‐related markers, including Nppa, Nppb, and Myh7, suggesting that Lgr6 deficiency exacerbates PO‐induced cardiac hypertrophy (Figure 2E). Obvious perivascular and myocardial interstitial collagen deposition was observed in mice after TAC surgery, which was further aggravated by AAV9‐shLgr6 treatment (Figure 2F). Compared with AAV9‐null, AAV9‐shLgr6 treatment increased the cardiac protein level of Anp, brain natriuretic peptide (Bnp), collagen type I (Col1), and α‐smooth muscle actin (α‐Sma) in PO mice (Figure 2G,H). PO‐induced cardiac remodeling is often accompanied by cardiac dysfunction. Cardiac ultrasound evaluations revealed that PO mice with Lgr6 deficiency exhibited more severe cardiac dysfunction compared to mice with AAV9‐null, as shown by decreased ejection fraction (EF), fractional shortening (FS), and increased left ventricular posterior wall thickness at end‐diastole (LVPWd) (Figure 2I).

To directly assess the role of Lgr6 in cardiomyocyte enlargement, we constructed small interfering RNA (siRNA) for Lgr6 (siLgr6) and normal control (siNC) to treat NRCMs (Figure S4A, Supporting Information). Compared to siNC, siLgr6 significantly increased the cellular surface area of cardiomyocytes under PE conditions (Figure S4B, Supporting Information). Consistently, Lgr6 deficiency promoted the mRNA and protein expression of hypertrophy‐related markers, including Nppa, Nppb, and Myh7 (Figure S4C,D, Supporting Information). In addition, Lgr6 deficiency exacerbated cyclic stretching‐induced hypertrophy in NRCMs, as shown by cell size and the expression of Nppa, Nppb, and Myh7 (Figure S5A–C, Supporting Information). These results suggest that Lgr6 deficiency exacerbates PE‐induced cardiomyocyte hypertrophy.

### 2.3 Cardiomyocyte Specific Lgr6 Overexpression Inhibits PO‐Induced Cardiac Hypertrophy in Mice

**Figure 3:**
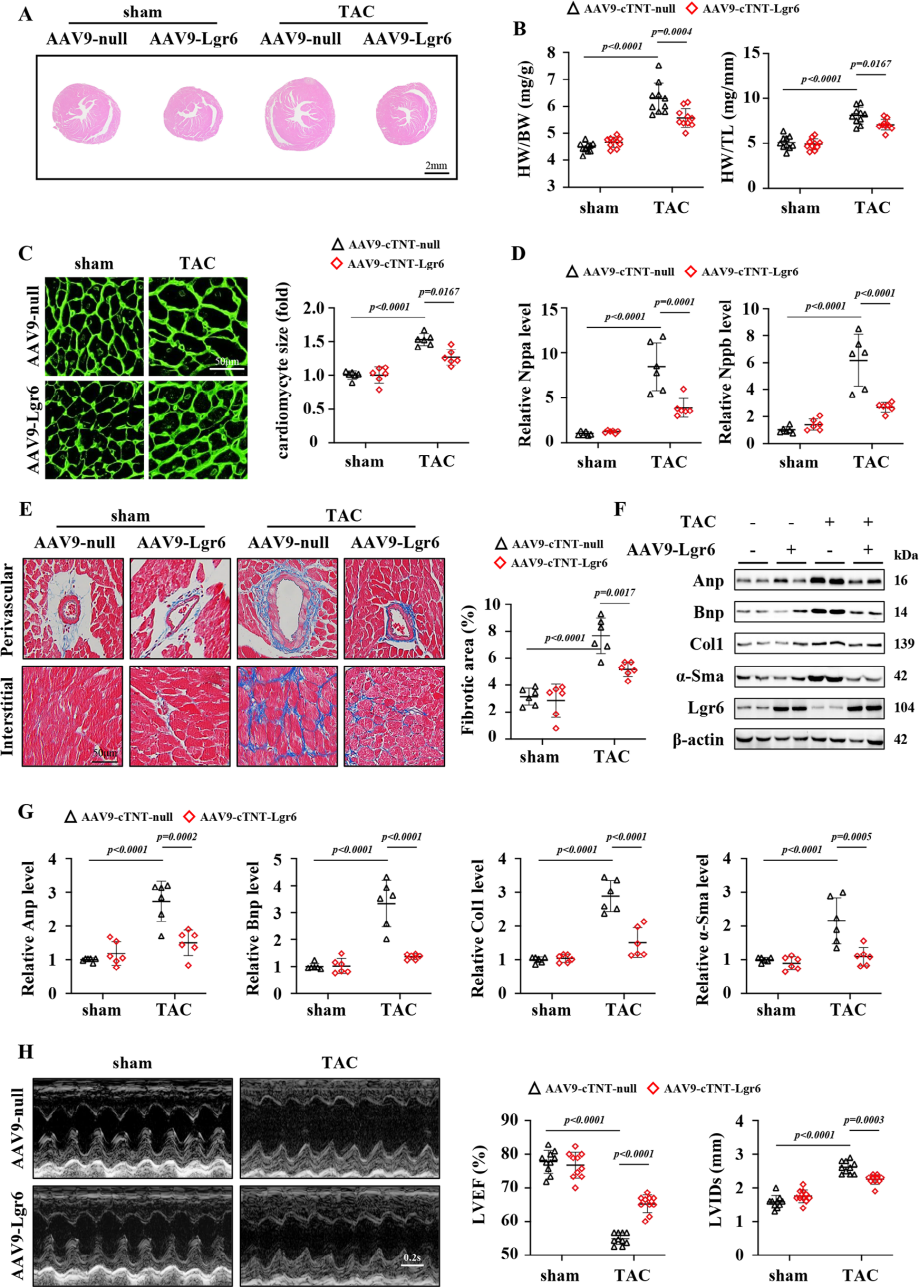
Cardiomyocyte specific Lgr6 overexpression inhibits PO‐induced cardiac hypertrophy in mice. A) Representative images of H&E‐stained sections (n = 6). B) Heart weight (mg) and body weight (g) ratio (HW/BW), heart weight (mg) and tibia length (mm) ratio (HW/TL) (n = 10). C) Representative images of WGA‐stained sections, and quantification of the cardiomyocyte cross‐sectional area based on WGA staining (n = 6). D) Real‐time quantitative reverse transcription polymerase chain reaction analysis comparing Nppa and Nppb mRNA expression in sham and TAC hearts (n = 6). E) Representative cardiac Masson trichrome staining in the peripheral (top) and interstitial (bottom) areas and quantification of the LV collagen volume (n = 6). F,G) Representative immunoblots and corresponding quantification showing cardiac protein expression of Anp, Bnp, Col1 and α‐Sma in sham and TAC mice (n = 6). H) Representative M‐mode echocardiographic images of the left ventricle and corresponding parameters showing cardiac function (n = 10). Significance was assessed by 2‐way ANOVA and Tukey's post hoc test. The data are shown as the mean±SD. Anp, atrial natriuretic peptide; Bnp, brain natriuretic peptide; Col1, collagen 1; α‐Sma, α‐smooth muscle actin.

To validate the functional role of Lgr6 in pathological cardiac hypertrophy, we generated AAVs carrying the cTnT promoter to selectively enhance Lgr6 expression in mouse cardiomyocytes (AAV9‐cTnT‐Lgr6 and AAV9‐cTnT‐null) (Figure S6, Supporting Information). Compared to AAV9‐null, mice with Lgr6 overexpression exhibited reduced heart volume and lower ratios of HW to BW and TL (**Figure** **3**A,B). The cardiomyocyte size of mice with Lgr6 overexpression was significantly smaller in PO mice, as evidenced by WGA staining (Figure 3C). Additionally, mice with Lgr6 overexpression showed downregulated mRNA levels of cardiac hypertrophy‐related markers, including Nppa and Nppb, suggesting that Lgr6 overexpression mitigated PO‐induced cardiac hypertrophy (Figure 3D). Compared AAV9‐null, AAV9‐Lgr6 treatment reduced the perivascular and myocardial interstitial collagen deposition in PO mice (Figure 3E). In addition, Lgr6 overexpression reduced the cardiac protein level of Anp, Bnp, Col1, and α‐Sma in PO mice (Figure 3F,G). Cardiac ultrasound evaluations revealed that mice with Lgr6 overexpression exhibited improved cardiac function compared to mice with AAV9‐null, as shown by increased EF and decreased left ventricular internal diameter during systole (LVIDs) (Figure 3H). In vitro, we transfected NRCMs with an adenovirus to overexpress Lgr6 (Ad‐Lgr6) (Figure S7A, Supporting Information). Compared to the control (Ad‐null), Ad‐Lgr6 significantly inhibited PE‐induced cardiomyocyte hypertrophy (Figure S7B,C, Supporting Information). In addition, Lgr6 overexpression attenuated cyclic stretching‐induced hypertrophy in NRCMs, as shown by cell size and the expression of Nppa, Nppb, and Myh7 (Figure S8A–C, Supporting Information). These results suggest that Lgr6 overexpression suppresses PO‐induced cardiac hypertrophy in mice.

### 2.4 Lgr6 Restores the Balance Between Glycolysis and Fatty Acid Metabolism in PO Mice

**Figure 4:**
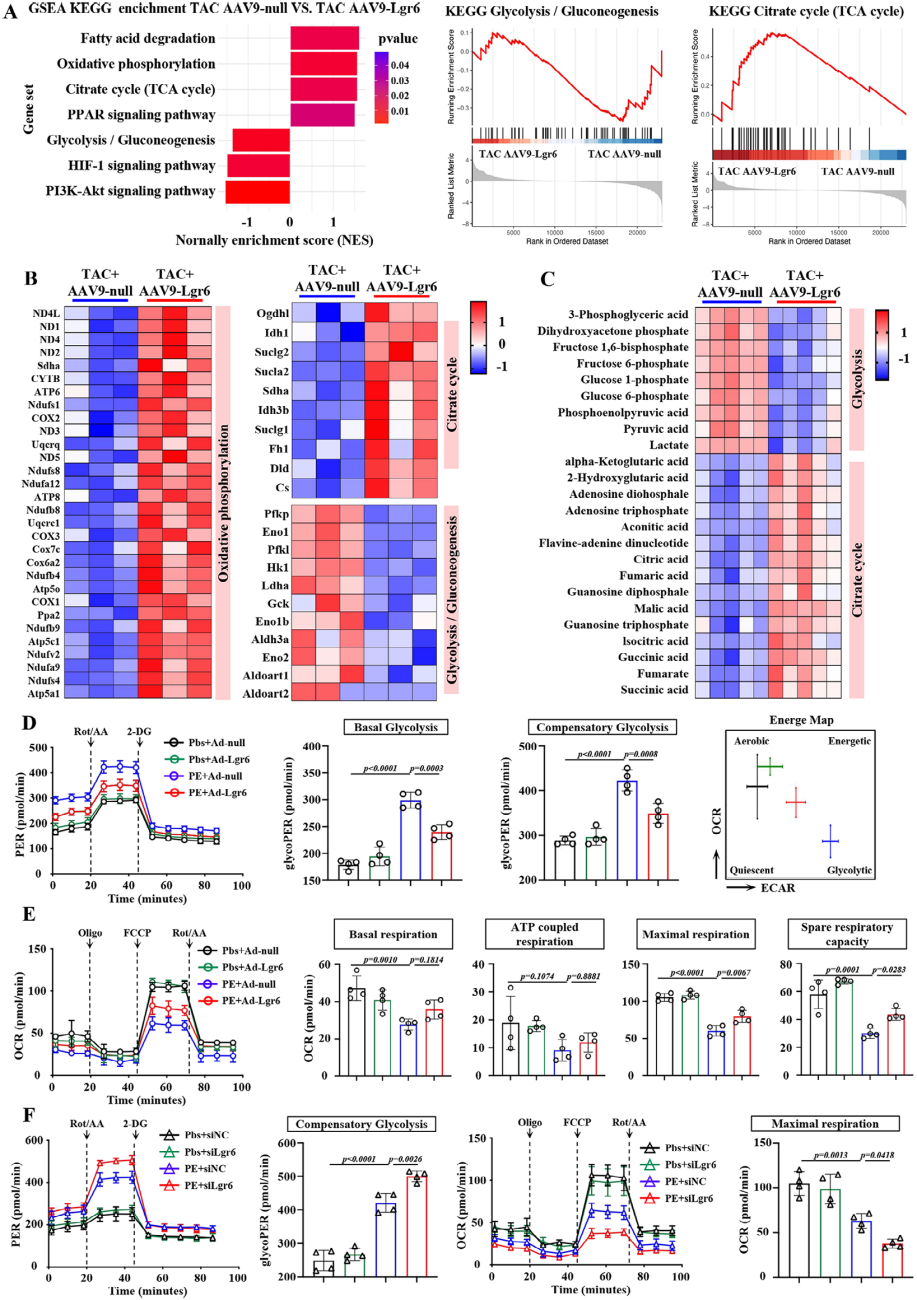
Lgr6 restores the balance between glycolysis and fatty acid metabolism in PO mice. A) Gene set enrichment analysis (GSEA) and enrichment plots showing KEGG fatty acid degradation, oxidative phosphorylation, citrate cycle (TCA cycle), PPAR signaling pathway, glycolysis/gluconeogenesis, HIF‐1 signaling pathway, and PI3K‐Akt signaling pathway. B) Heatmaps showing the cardiac expression of the differentially expressed genes resolved in KEGG oxidative phosphorylation, citrate cycle and glycolysis/gluconeogenesis pathway (n = 3). C) Analysis of liquid chromatography‐mass spectrometry‐based metabolomics on left ventricular tissue showing metabolites resolved in glycolysis and citrate cycle (n = 5). D) Glycolytic proton efflux rate (GlycoPER) profiles of NRCMs transfected with Ad‐null or Ad‐Lgr6 under sham or PE conditions using the Seahorse analyzer and glycoPER assay (n = 4). E) Mitochondrial respiration profiles of NRCMs transfected with Ad‐null or Ad‐Lgr6 under sham or PE conditions using the Seahorse analyzer (n = 4). F) Glycolytic proton efflux rate and mitochondrial respiration profiles of NRCMs transfected with siNC or siLgr6 under sham or PE conditions using the Seahorse analyzer (n = 4). Significance was assessed by 1‐way ANOVA and Tukey's post hoc test. The data are shown as the mean±SD.

Given the protective role of Lgr6 in pathological cardiac hypertrophy, we investigated its molecular mechanism in regulating cardiac hypertrophy. We conducted RNA‐sequencing analysis on left ventricular tissue from PO mice treated with AAV9‐null and AAV9‐Lgr6. We identified 706 differentially expressed genes (DEGs), including 238 up‐regulated genes and 468 down‐regulated genes (Figure S9A, Supporting Information). Kyoto encyclopedia of genes and genomes (KEGG) annotation revealed that Metabolic pathways had the largest number of DEGs (Figure S9B, Supporting Information). Additionally, GSEA KEGG analysis highlighted significant enrichment of Fatty acid degradation, Oxidative phosphorylation, Citrate cycle (TCA cycle), and PPAR signaling pathway among the up‐regulated genes (**Figure** **4**A). Conversely, Glycolysis/Gluconeogenesis, HIF‐1 signaling pathway, and PI3K‐Akt signaling pathway were significantly enriched among the down‐regulated genes (Figure 4A). Compared to AAV9‐null controls, AAV9‐Lgr6 significantly upregulated genes associated with oxidative phosphorylation and the citrate cycle, while concurrently suppressing those involved in glycolysis and gluconeogenesis (Figure 4B). To further elucidate these metabolic shifts, we examined the mRNA expression levels of key genes in the glycolysis and citrate cycle pathways in mouse hearts (Figure S10A, Supporting Information). Consistent with RNAseq results, Lgr6 overexpression robustly inhibited the expression of glycolysis‐related genes and enhanced the expression of citrate cycle‐related genes in the hearts of PO mice (Figure S10A, Supporting Information). Moreover, Lgr6 overexpression led to a significant reduction in lactate levels in PO hearts, indicative of suppressed glycolysis (Figure S10B, Supporting Information). Additionally, Lgr6 overexpression decreased the NADH/NAD⁺ ratio and increased ATP levels in PO hearts, suggesting a restoration of oxidative phosphorylation (Figure S10C,D, Supporting Information). To explore the link between Lgr6 and PO‐induced metabolic reprogramming further, we performed liquid chromatography‐mass spectrometry‐based metabolomics on left ventricular tissue from PO mice (Figure 4C). Lgr6 overexpression down‐regulated Glycolysis‐related metabolites but up‐regulated Citrate cycle‐related metabolites, suggesting that Lgr6 promotes cardiometabolic reprogramming in PO mice (Figure 4C). Fatty acid metabolism plays a crucial role in myocardial energy metabolism. We further assessed the levels of free fatty acids (FFAs) and triglycerides (TGs) in myocardial tissue. Compared to sham hearts, PO hearts exhibited increased levels of FFAs and TGs (Figure S10E,F, Supporting Information). However, Lgr6 overexpression significantly reduced the levels of FFAs and TGs in PO hearts (Figure S10E,F, Supporting Information). Consistent with these findings, metabolomics results indicated that Lgr6 overexpression markedly decreased the levels of FFAs and TGs in PO hearts, suggesting that Lgr6 overexpression promotes fatty acid metabolism in the hearts of PO mice (Figure S10G, Supporting Information). We ultimately evaluated the impact of Lgr6 overexpression on mitochondria and found that Lgr6 overexpression reduced the number of damaged mitochondria in cardiomyocytes of PO mice (Figure S10H, Supporting Information). These results demonstrate that Lgr6 restores the balance between glycolysis and fatty acid metabolism in PO mice.

Given the impact of Lgr6 on NRCMs hypertrophy, we explored metabolic activities associated with Lgr6 expression in NRCMs. Using a Seahorse XF24 extracellular flux analyzer, we monitored real‐time cellular oxygen consumption rate (OCR) and extracellular acidification rate to estimate mitochondrial respiration and glycolysis. Compared to PBS treatment, NRCMs treated with PE exhibited higher basal and compensatory glycolysis rates, along with lower basal, ATP production‐coupled, maximal and spare respiratory OCR (Figure 4D,E). These findings suggest metabolic reprogramming in hypertrophic cardiomyocytes with PE condition. Additionally, Ad‐Lgr6 effectively inhibited glycolysis and promoted mitochondrial respiration of NRCMs under PE conditions compared to Ad‐null (Figure 4D,E). Consistent with the Seahorse results, Lgr6 overexpression suppressed the expression of glycolysis‐related genes and enhanced the expression of oxidative phosphorylation‐related genes in NRCMs under PE stimulation (Figure S11A, Supporting Information). Moreover, Lgr6 overexpression inhibited the accumulation of lactate and FFAs in NRCMs under PE conditions, decreased the NADH/NAD⁺ ratio, and increased ATP production (Figure S11B–E, Supporting Information). Additionally, Lgr6 overexpression alleviated mitochondrial damage in NRCMs induced by PE (Figure S11F, Supporting Information). These findings collectively suggest that Lgr6 overexpression promotes beneficial metabolic reprogramming in NRCMs under PE conditions.

Conversely, Lgr6 deficiency exacerbated glycolysis and suppressed mitochondrial respiration in NRCMs under PE conditions (Figure 4F). Overall, these findings highlight Lgr6 as a critical regulator of PE‐induced metabolic reprogramming in NRCMs.

### 2.5 Lgr6 Regulates PO‐Induced Cardiac Metabolic Reprogramming in a PPARα‐Dependent Manner

**Figure 5:**
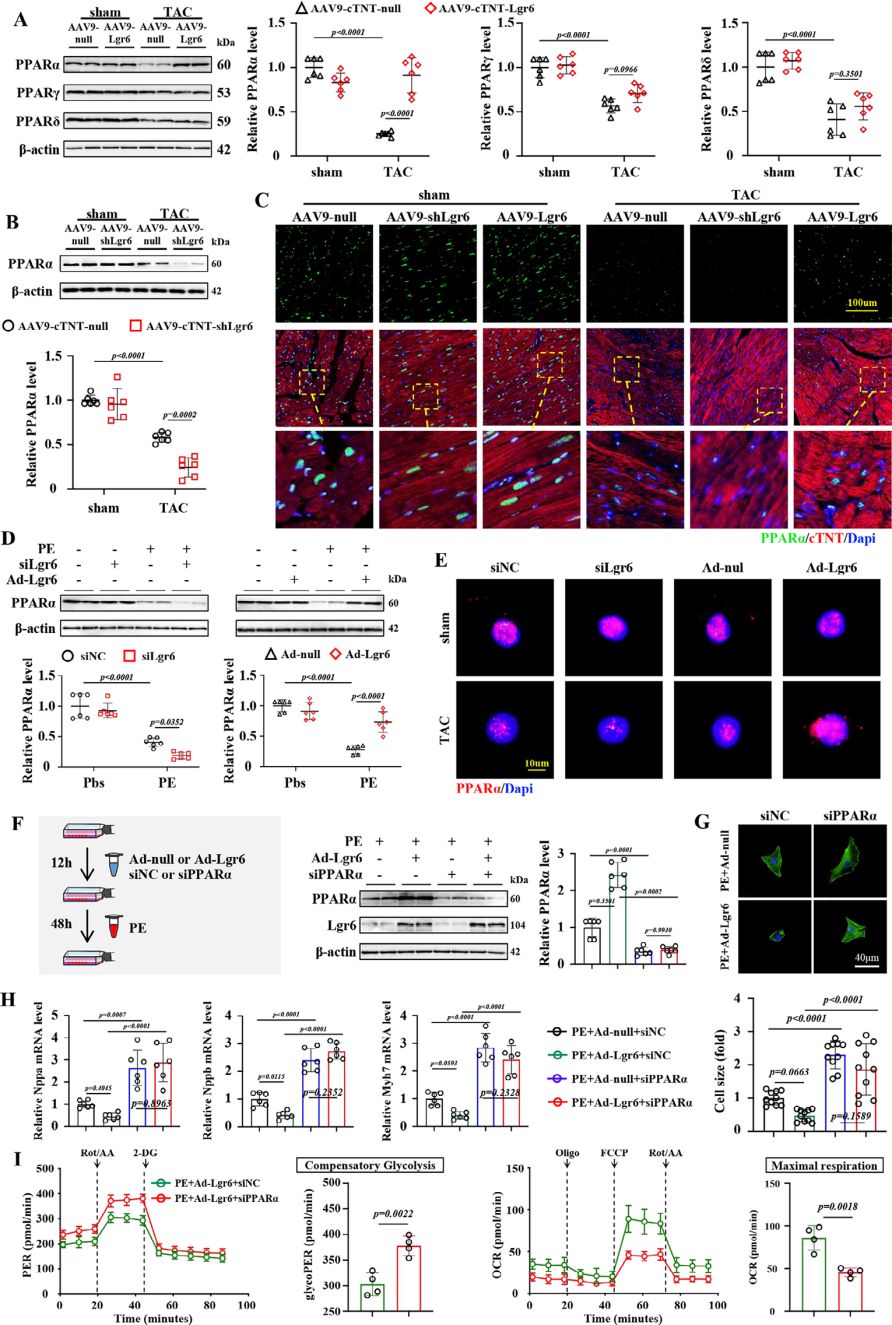
Lgr6 regulates PO‐induced cardiac metabolic reprogramming in a PPARα‐dependent manner. A) Representative immunoblots and corresponding quantification showing cardiac PPARα, PPARγ, and PPARδ (n = 6). B) Representative immunoblots and corresponding quantification showing cardiac PPARα (n = 6). C) Representative immunofluorescence staining images for cTNT and PPARα (n = 6). D) Representative immunoblots and corresponding quantification showing PPARα in PE‐treated NRCMs (n = 6). E) Representative immunofluorescence staining images for PPARα in NRCMs (n = 6). F) Schematic diagram depicting the experimental strategy for siPPARα treatment. Representative immunoblots and corresponding quantification showing PPARα (n = 6). G) Representative phalloidin staining images and corresponding quantification showing cell size of NRCMs (n = 10). H) Real‐time quantitative reverse transcription polymerase chain reaction analysis comparing Nppa, Nppb and Myh7 mRNA expression (n = 6). I) Glycolytic proton efflux rate and mitochondrial respiration profiles of NRCMs transfected with siNC or PPARα under PE and Lgr6 overexpression conditions using the Seahorse analyzer (n = 4). The data are shown as the mean±SD. A, B and D were analyzed by 2‐way ANOVA and Tukey's post hoc test. F, G and H were analyzed by 1‐way ANOVA and Tukey's post hoc test. I was assessed by two‐tailed unpaired Student's t‐test.

In our GSEA analysis, we observed a significant enrichment of the KEGG PPAR signaling pathway in hearts treated with Lgr6 overexpression (Figure S12A, Supporting Information). Specifically, Lgr6 overexpression upregulated several key genes involved in the PPAR signaling pathway in PO hearts (Figure S12B, Supporting Information). This pathway is known to regulate cardiac hypertrophy through cardiac metabolic reprogramming.^[^ ^20^ ^]^ We subsequently evaluated the expression levels of PPARα, PPARγ, and PPARδ in mouse hearts (**Figure** **5**A). PO mice showed reduced levels of cardiac PPARα, PPARγ, and PPARδ compared to the sham group. Notably, Lgr6 overexpression significantly upregulated PPARα expression, but not PPARγ or PPARδ, compared to AAV9‐null (Figure 5A). AAV9‐shLgr6 further reduced cardiac PPARα expression in PO mice (Figure 5B). Immunofluorescence results further showed that Lgr6 knockdown reduced while Lgr6 overexpression increased cardiomyocyte PPARα expression in PO mice (Figure 5C). Consistently, Lgr6 overexpression increased, while Lgr6 knockdown suppressed, PPARα expression in NRCMs under PE conditions as shown by blots and immunofluorescence (Figure 5D,E). These findings suggest that Lgr6 may regulate PO‐induced cardiometabolic reprogramming by modulating PPARα expression.

To investigate the role of PPARα in Lgr6‐regulated metabolic reprogramming of cardiomyocytes, we first utilized small interfering RNA (siPPARα) to inhibit PPARα expression (Figure 5F). Compared to the control (siNC), siPPARα significantly aggravated hypertrophy in NRCMs under Lgr6‐overexpressing and PE conditions, as evidenced by increased cell size, Nppa, Nppb, and Myh7 expression (Figure 5G‐H). Additionally, siPPARα significantly enhanced glycolysis and inhibited mitochondrial respiration in NRCMs under Lgr6‐overexpressing and PE conditions, indicating metabolic reprogramming (Figure 5I). Furthermore, we used adenovirus‐transfected NRCMs to overexpress PPARα (Ad‐PPARα) and found that PPARα overexpression inhibited hypertrophy in NRCMs under Lgr6 deficiency and PE conditions (Figure S13A–C, Supporting Information). These findings suggest that Lgr6 may regulate PE‐induced cardiomyocyte hypertrophy and metabolic reprogramming in a PPARα‐dependent manner.

We then transfected AAV9‐cTnT‐PPARα and AAV9‐cTnT‐shLgr6 into the hearts of mice to further investigate the role of PPARα in Lgr6‐regulated cardiac hypertrophy (Figure S14A, Supporting Information). Compared to AAV9‐null, AAV9‐PPARα treatment significantly increased cardiac PPARα expression four weeks post‐injection (Figure S14B, Supporting Information). The mice subsequently underwent TAC surgery. Compared to AAV9‐null, AAV9‐PPARα treatment attenuated pressure overload‐induced cardiac dysfunction, hypertrophy, and fibrosis in mice with Lgr6 deficiency (Figure S14C–F, Supporting Information).

### 2.6 Lgr6 Deubiquitinates and Stabilizes PPARα by Upregulating USP4 Expression in PO Mice

**Figure 6:**
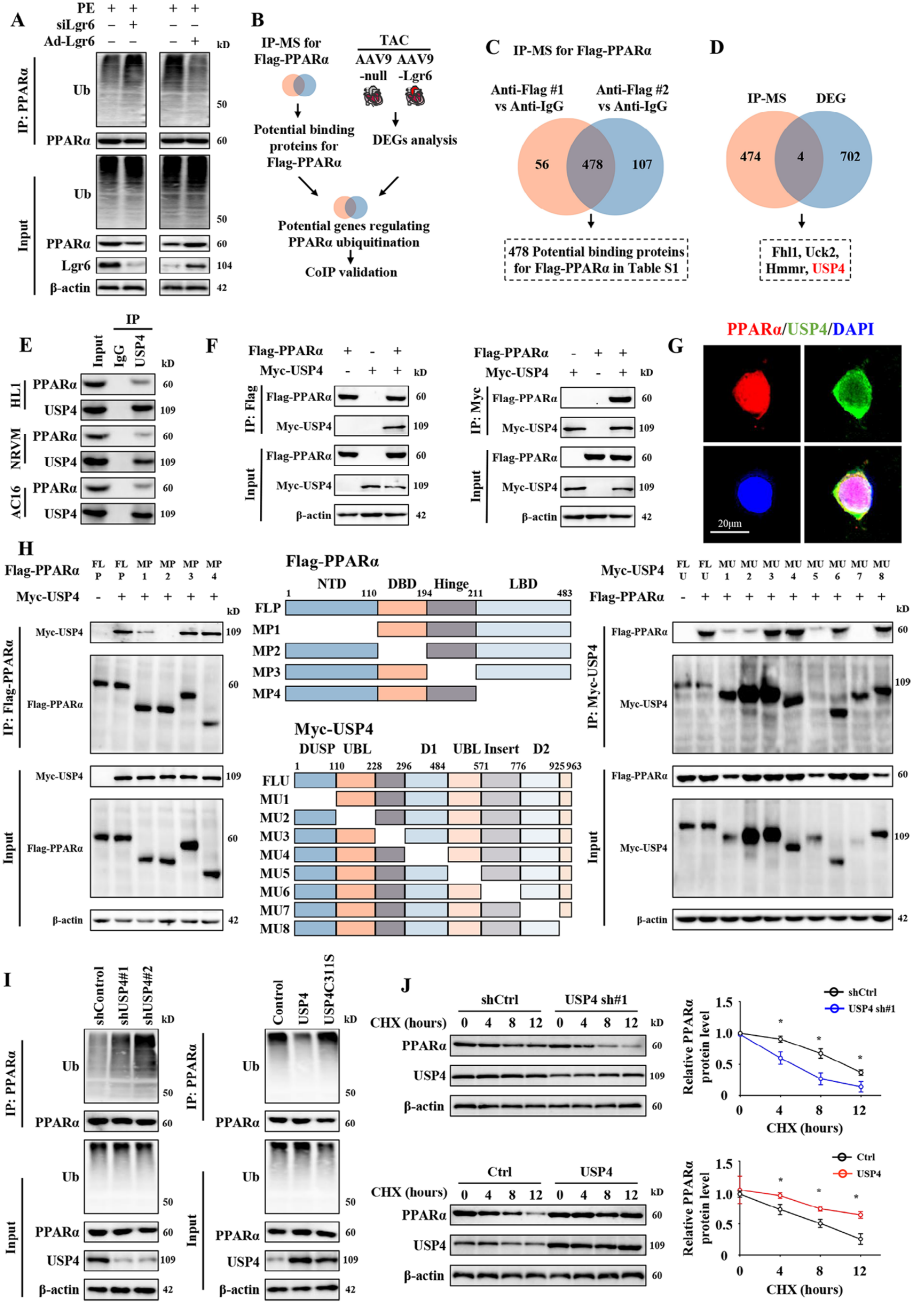
Lgr6 treatment deubiquitinates and stabilizes PPARα by upregulating USP4 expression in PO mice. A) Representative immunoblots showing ubiquitination of PPARα in PE‐treated NRCMs with Lgr6 deficiency or overexpression (n = 6). B) Diagram of workflow for the selection of potential genes regulating PPARα ubiquitination from the IP‐MS analysis and differently expressed genes identified in RNAseq analysis. C) Flag‐PPARα was overexpressed in HEK293T cells. IP‐MS analysis for Flag‐PPARα was performed to find proteins binding to PPARα (n = 2). D) Four identical target genes were selected after comparing between proteins from IP‐MS analysis and differently expressed genes identified in RNAseq analysis. E) Representative immunoblots showing the binding of PPARα and USP4 in different cardiomyocytes (n = 6). F) HEK293T cells were transfected for 24 hours with plasmids encoding either Flag‐PPARα or Myc‐USP4 alone or in combination. Cell lysates were immunoprecipitated with Flag or Myc antibodies, and immunoblotting was performed using Flag or Myc antibodies (n = 6). G) Triple immunofluorescence staining for USP4 (green), PPARα (red), and nuclei (DAPI, blue) was performed in NRCMs (n = 6). H) Schematic diagram showing the constructs expressing full‐length PPARα and its truncated fragments (Top panel), or the structure of USP4 and deletion constructs used (bottom panel). Flag‐tagged full‐length or truncated PPARα were co‐expressed with Myc‐tagged full‐length or truncated USP4 in HEK293T cells. Cell lysates were immunoprecipitated with Flag or Myc antibodies, and immunoblotting was performed using Flag or Myc antibodies (n = 6). I) Ubiquitination assay of PPARα in HEK293T cells transfected with shUSP4#1/2 or HEK293T cells with wild‐type USP4 or catalytically inactive mutant USP4C311S (n = 6). J) HEK293T were infected with shCtrl, shUSP4#1, Ctrl, or USP4 plasmid, and then treated with CHX (10 µmol L^−1^) for the indicated time periods. Representative immunoblots and corresponding quantification showing PPARα level (n = 6). Significance was assessed by 2‐way ANOVA and Tukey's post hoc test. The data are shown as the mean±SD.

Given that Lgr6 regulates PO‐induced cardiac metabolic reprogramming in a PPARα‐dependent manner, we explored the mechanism by which Lgr6 regulates PPARα expression. RNAseq data indicated that Lgr6 overexpression did not affect PPARα mRNA expression, suggesting that Lgr6 may regulate the post‐translational modification of PPARα (Figure S15A, Supporting Information). We first assessed the ubiquitination of PPARα, one of the most common protein modifications. Immunoblotting results showed that Lgr6 overexpression inhibited, while Lgr6 deficiency promoted, the ubiquitination of PPARα in PO‐induced mouse hearts (Figure S15B, Supporting Information). Consistently, Lgr6 deficiency increased, while Lgr6 overexpression reduced, the ubiquitination of PPARα in NRCMs under PE conditions (**Figure** **6**A). These results suggest that Lgr6 may promote metabolic reprogramming by regulating PPARα ubiquitination.

To explore the mechanism by which Lgr6 regulates PPARα ubiquitination, we designed a strategy to identify potential targets involved in this process (Figure 6B). We first transfected Flag‐PPARα plasmid into HEK‐293T cells. Using Anti‐Flag and Anti‐IgG magnetic beads for immunoprecipitation combined with mass spectrometry (IP‐MS), we identified 478 proteins that may bind to PPARα (Figure 6C; Table S1). We then compared these PPARα‐binding proteins with the DEGs obtained from RNAseq and identified four potential targets: FHL1, UCK2, HMMR, and USP4 (Figure 6D). Notably, USP4 has been previously reported to promote the stability of multiple proteins through deubiquitination.^[^ ^21^ ^]^ Lgr6 deficiency suppressed, whereas Lgr6 overexpression enhanced the expression of USP4 in both PO hearts and NRCMs under PE stimulation (Figure S16A,B, Supporting Information). We speculate that Lgr6 may promote the deubiquitination and stabilization of PPARα by upregulating USP4 expression.

We first found that USP4 can bind to PPARα in HL1 mouse cardiomyocytes, NRCMs, and AC16 human cardiomyocytes (Figure 6E). We then verified the specific interaction between USP4 and PPARα in HEK‐293T cells exogenously expressing Myc‐USP4 and Flag‐PPARα (Figure 6F). Moreover, USP4 and PPARα were primarily colocalized in the cytoplasm of NRCMs as shown by immunofluorescence analysis (Figure 6G). To investigate the specific regions of interaction between USP4 and PPARα, we constructed truncated mutant fragments of both proteins to identify the binding sites (Figure 6H). Transfection experiments in HEK‐293T cells showed that deletion of amino acids 776–925 (D2) of USP4 impaired its ability to bind to PPARα. For PPARα, deletion of amino acids 110–194 (DBD) abolished its binding to USP4.

As a deubiquitinating enzyme, USP4 may promote the deubiquitination and stabilization of PPARα. To test this hypothesis, we first knocked down USP4 expression in HEK‐293T cells using two shRNAs and observed a decrease in PPARα protein levels (Figure 6I). Ectopic expression of wild‐type USP4, but not the catalytically inactive mutant USP4C311S, increased PPARα protein levels (Figure 6I). We then found that the proteasome inhibitor MG132, but not the autophagosome pathway inhibitor chloroquine, reversed the reduction in PPARα protein levels caused by USP4 knockdown (Figure S15C, Supporting Information). To further examine the effect of USP4 on PPARα protein stability, we used cycloheximide to block protein synthesis (Figure 6J). PPARα showed rapid degradation in USP4 knockdown cells, while it exhibited a significantly longer half‐life in USP4‐overexpressing cells (Figure 6J). Additionally, both the DBD fragment deletion of PPARα and the D2 fragment deletion of USP4 effectively abolished the deubiquitination effect of USP4 on PPARα (Figure S15D,E, Supporting Information). These results indicate that USP4 strictly controls the stability of PPARα protein.

To determine which type of PPARα ubiquitination is affected by USP4, we co‐transfected Myc‐USP4, Flag‐PPARα, and seven lysine‐specific ubiquitin mutants (K6, K11, K27, K29, K33, K48, and K63) into HEK‐293T cells. USP4 specifically cleaved the K48‐linked polyubiquitinated chains of the PPARα protein (Figure S15F, Supporting Information). To identify the PPARα lysine sites targeted by USP4, we predicted potential ubiquitination sites in the DBD fragment of PPARα using an online database (https://sumo.biocuckoo.cn) (Figure S15G, Supporting Information). The results indicated that 11 sites in the DBD, including K123, K133, K138, K144, K147, K148, K152, K160, K181, K185, and K182, may be modified by ubiquitination. We constructed K‐R mutations at these 11 sites and found that USP4 did not deubiquitinate PPARα‐K152R (Figure S15H, Supporting Information). These results suggest that USP4 enhances the stability of PPARα by selectively removing K48‐linked polyubiquitinated chains from the K152 site of the PPARα protein.

We then knocked down USP4 expression in NRCMs using siUSP4 and found that USP4 deficiency promoted Anp expression and inhibited PPARα expression (Figure S16C,D, Supporting Information). Conversely, USP4 overexpression inhibited Anp expression and promoted PPARα expression in NRCMs under Lgr6 deficiency and PE conditions (Figure S16E,F, Supporting Information). We subsequently constructed an AAV9‐cTNT‐USP4 virus to specifically overexpress USP4 in the cardiomyocytes (Figure S17A,B, Supporting Information). USP4 overexpression effectively alleviated PO‐induced cardiac dysfunction, hypertrophy, and fibrosis in mice with Lgr6 deficiency (Figure S17C–F, Supporting Information). Additionally, USP4 overexpression suppressed Anp expression and restored PPARα expression in the hearts of mice with Lgr6 deficiency (Figure S17G, Supporting Information). These results suggest that Lgr6 regulates PPARα expression and cardiac hypertrophy in a USP4‐dependent manner in hypertrophic cardiomyocytes.

### 2.7 Lgr6 Promotes USP4 Expression Through cGMP/PKG/CREB1 Signaling Pathway in PO Mice

**Figure 7:**
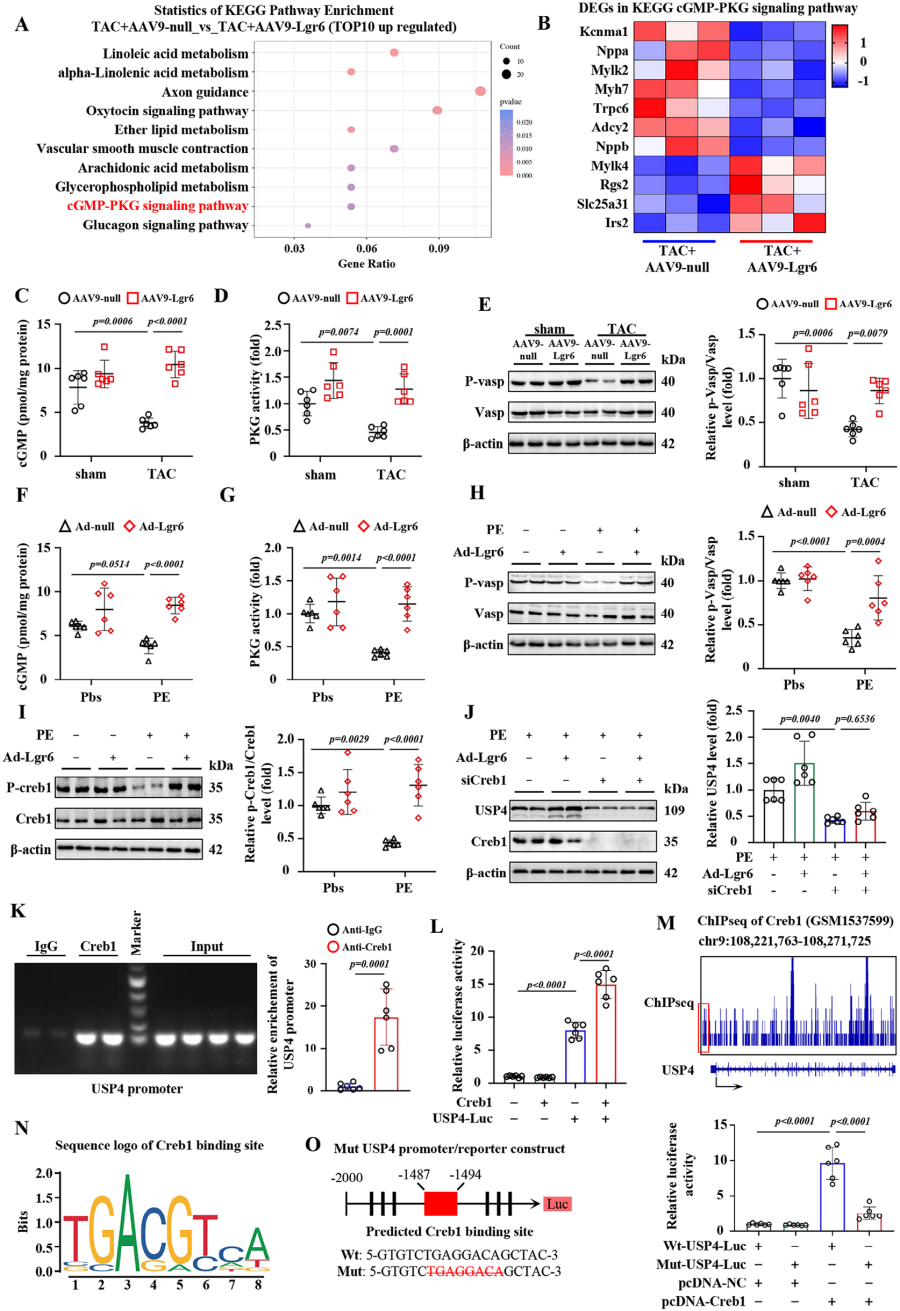
Lgr6 promotes USP4 expression through cGMP/PKG/CREB1 signaling pathway in PO mice. A) Kyoto Encyclopedia of Genes and Genomes (KEGG) functional enrichment analysis of upregulated genes in TAC+Lgr6 hearts. B) Heatmaps showing the cardiac expression of the differentially expressed genes resolved in KEGG cGMP‐PKG signaling pathway (n = 3). C) Cardiac cGMP expression level (n = 6). D) Cardiac PKG activity (n = 6). E) Representative immunoblots and corresponding quantification showing the phosphorylation level of cardiac VASP (n = 6). F) cGMP expression level in NRCMs (n = 6). G) PKG activity in NRCMs (n = 6). H) Representative immunoblots and corresponding quantification showing the phosphorylation level of VASP in NRCMs (n = 6). I) Representative immunoblots and corresponding quantification showing the phosphorylation level of CREB1 in NRCMs (n = 6). J) Representative immunoblots and corresponding quantification showing USP4 in NRCMs (n = 6). K) ChIP‐qPCR assay in NRCMs for CREB1 or IgG occupancy at USP4 promoter fragments (n = 6). L) Luciferase activation driven by USP4 promoter after normalization to Renilla luciferase in HEK293T cells (n = 6). M) IGV tracks showing CREB1 ChIP‐Seq signals at USP4 gene locus. N) Consensus DNA‐binding motifs of CREB1 according to JASPAR database. O) Schematic diagram of the construction of wild type and mutant luciferase reporter plasmids of USP4 promoter. Luciferase activation driven by the wild type or mutant USP4 promoter after normalization to Renilla luciferase in HEK293T cells (n = 6). The data are shown as the mean±SD. C, D, E, F, G, H and I were analyzed by 2‐way ANOVA and Tukey's post hoc test. J, L and Q were analyzed by 1‐way ANOVA and Tukey's post hoc test. K was assessed by two‐tailed unpaired Student's t‐test.

KEGG enrichment analysis of upregulated DEGs revealed significant enrichment of the cGMP‐PKG signaling pathway (**Figure** **7**A‐B). Cyclic guanosine monophosphate (cGMP) is one of the main second messengers critically involved in cardiac electrophysiology, hypertrophy, and contractility regulation.^[^ ^22^ ^]^ Activation of the cGMP‐PKG signaling pathway helps to alleviate PO‐induced cardiac hypertrophy.^[^ ^22^ ^]^ Lgr6 overexpression effectively increased the expression level of cGMP and the activity of PKG in the hearts of PO mice compared to AAV9‐null (Figure 7C,D). Lgr6 overexpression also promoted the phosphorylation level of vasodilator‐stimulated phosphoprotein (p‐VASP), an indicator of cGMP/PKG activation, in the hearts of PO mice (Figure 7E). In vitro, Lgr6 overexpression enhanced the expression of cGMP, the activity of PKG, and the phosphorylation level of VASP in NRCMs under PE conditions (Figure 7F–H). These results suggest that Lgr6 activates the cGMP‐PKG signaling pathway both in vivo and in vitro. Treatment with the PKG antagonist KT5823 effectively inhibited the expression of p‐VASP and USP4 in NRCMs under PE and Lgr6 overexpression conditions, suggesting that Lgr6 regulates USP4 expression in a PKG‐dependent manner in cardiomyocytes of PO mice (Figure S18, Supporting Information).

As one of the classic downstream signals of PKG, cAMP response element binding protein 1 (CREB1) has been reported to regulate PO‐induced cardiac hypertrophy.^[^ ^23^ ^]^ In our study, Lgr6 overexpression effectively promoted the phosphorylation level of CREB1 in NRCMs under PE conditions (Figure 7I). Treatment with siCREB1 inhibited the expression of USP4 in NRCMs under Lgr6 overexpression and PE conditions, suggesting that Lgr6 may promote USP4 expression in cardiomyocytes by regulating CREB1 (Figure 7J). Chromatin immunoprecipitation (ChIP) results indicated that CREB1 could bind to the USP4 promoter (Figure 7K). The dual‐luciferase reporter assay demonstrated that CREB1 overexpression effectively promoted USP4 transcription (Figure 7L). The ChIP‐seq online database of CREB1 (GSM1537599) suggested that CREB1 may bind to the 1487–1494 fragment upstream of the USP4 promoter (Figure 7M,N). We designed a mutant USP4 promoter reporter gene and found that the mutant USP4 significantly reduced CREB1 binding to the USP4 promoter compared to the wild‐type USP4 (Figure 7O). These results suggest that Lgr6 regulates USP4 expression through the cGMP/PKG/CREB1 signaling pathway in PO mice.

### 2.8 Maresin1 Protects Mice from PO‐Induced Cardiac Hypertrophy via Activating Lgr6

**Figure 8:**
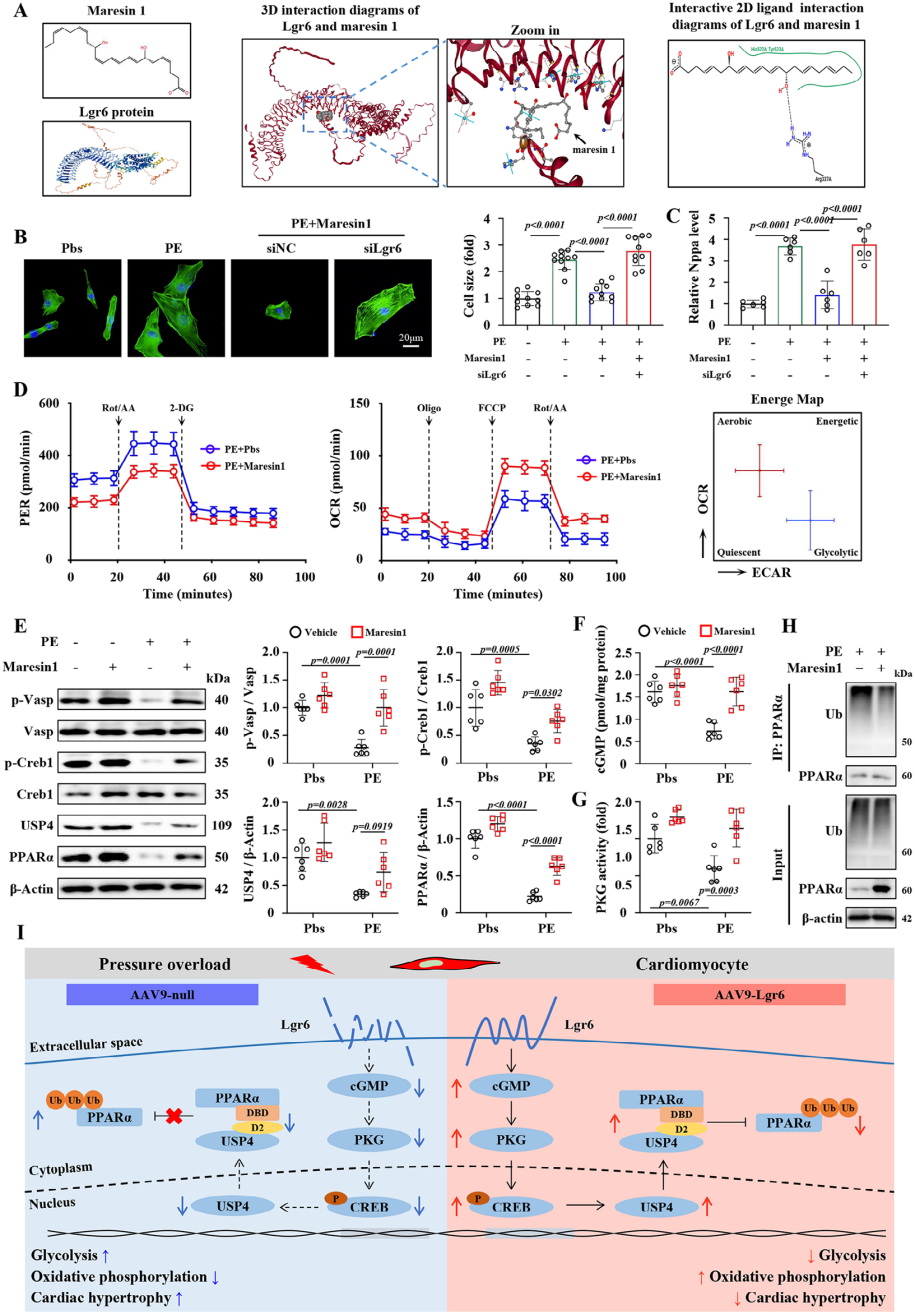
Maresin1 protects cardiomyocytes from PE‐induced hypertrophy via activating Lgr6. A) Schematic diagram showing the binding of Lgr6 and maresin1. B) Representative phalloidin staining images and corresponding quantification showing cell size of NRCMs (n = 6). C) Real‐time quantitative reverse transcription polymerase chain reaction analysis comparing Nppa mRNA expression (n = 6). D) Glycolytic proton efflux rate and mitochondrial respiration profiles of NRCMs treated with PBS or maresin1 under PE conditions using the Seahorse analyzer (n = 6). E) Representative immunoblots and corresponding quantification showing the level of phosphorylated VASP, CREB1, USP4 and PPARα in NRCMs (n = 6). F) cGMP expression level in NRCMs (n = 6). G) PKG activity in NRCMs (n = 6). H) Ubiquitination assay of PPARα in NRCMs (n = 6). I) Proposed mechanisms. Lgr6 overexpression ameliorates cardiac hypertrophy by regulating metabolic reprogramming through USP4‐PPARα pathway. The data are shown as the mean±SD. E and F were analyzed by 2‐way ANOVA and Tukey's post hoc test. B was analyzed by 1‐way ANOVA and Tukey's post hoc test.

Having found that Lgr6 can regulate PO‐induced cardiac hypertrophy and metabolic reprogramming through the USP4/PPARα signaling pathway, we explored whether activation of Lgr6 using Maresin1, a selective agonist of Lgr6, could play a protective role in cardiac hypertrophy and offer clinical therapeutic potential (**Figure** **8**A). We first evaluated the role of Maresin1 in PE‐induced hypertrophy and metabolic remodeling of NRCMs in vitro. Maresin1 treatment effectively alleviated PE‐induced cell size enlargement and Nppa expression in NRCMs, which were inhibited by Lgr6 deficiency (Figure 8B,C). Furthermore, Maresin1 treatment inhibited glycolysis and promoted mitochondrial respiration in NRCMs under PE conditions, indicating metabolic reprogramming (Figure 8D). Mechanistically, Maresin1 activated the cGMP/PKG/CREB1/USP4 pathway and promoted the deubiquitination and stabilization of PPARα in NRCMs with PE condition (Figure 8E–H). In vitro experiments suggested that the Maresin1/Lgr6 axis inhibits PE‐induced cardiomyocyte hypertrophy and metabolic reprogramming.

We then further explored the therapeutic role of Maresin1 in PO‐induced cardiac hypertrophy in vivo. We first performed TAC surgery on mice to successfully induce cardiac hypertrophy. Maresin1 (2 µg kg^−1^ day^−1^) and vehicle were injected into the peritoneal cavity of mice 4 weeks after TAC surgery (Figure S19A, Supporting Information). Compared to the vehicle, four weeks of Maresin1 treatment effectively alleviated PO‐induced cardiac dysfunction (Figure S19B, Supporting Information). Additionally, after 8 weeks of TAC, Maresin1 alleviated PO‐induced cardiac hypertrophy and fibrosis, and promoted the deubiquitination and stabilization of PPARα through the PKG/CREB1/USP4 pathway (Figure S19C–H, Supporting Information). Taken together, Maresin1 alleviated PO‐induced cardiac dysfunction and hypertrophy, highlighting its potential for treating cardiac hypertrophy and heart failure.

## 3 Discussion

In this study, we discovered that cardiomyocyte Lgr6 expression decreased in PO mouse hearts. Lgr6 deficiency worsened, while Lgr6 overexpression alleviated, PO‐induced cardiac hypertrophy, dysfunction, and metabolic reprogramming. Mechanistically, Lgr6 enhances the deubiquitination and stabilization of PPARα by regulating the PKG/CREB1/USP4 signaling pathway in PO mice. Overexpressing PPARα and USP4 mitigated the exacerbation of PO‐induced cardiac hypertrophy and dysfunction caused by Lgr6 deficiency. Additionally, Maresin1 regulates PO‐induced cardiac hypertrophy and metabolic reprogramming in a Lgr6‐dependent manner. In summary, Lgr6 plays an important role on PO‐induced cardiac hypertrophy and may serve as a potential therapeutic target (Figure 8I).

Myocardial energy metabolism disorder is a key pathological basis of cardiac hypertrophy.^[^ ^2^ ^]^ The strategy of targeting myocardial energy metabolism to improve heart failure prognosis has gained increasing attention. Inhibiting cardiomyocytes glycolysis or promoting oxidative phosphorylation can effectively alleviate pathological cardiac hypertrophy.^[^ ^5^ ^]^ Our study is the first to report that cardiomyocyte Lgr6 inhibits PO‐induced glycolysis and promotes oxidative phosphorylation. Lgr6‐mediated metabolic reprogramming may be the mechanism by which Lgr6 overexpression attenuates PO‐induced cardiac hypertrophy and dysfunction. PPARα is a nuclear receptor predominantly found in tissues with high metabolic activity, such as the liver and heart.^[^ ^20^ ^]^ Upon activation, PPARα regulates the transcription of genes related to energy metabolism and is involved in the development of cardiovascular diseases.^[^ ^20^ ^]^ Previous studies have shown that the loss of PPARα in cardiomyocytes exacerbates PO‐induced cardiac hypertrophy and fibrosis.^[^ ^24^ ^]^ Conversely, PPARα overexpression or activation promotes cardiac fatty acid oxidation, improves myocardial energy metabolism, and alleviates PO‐induced cardiac dysfunction and remodeling.^[^ ^25^ ^]^ In our study, Lgr6 alleviated PO‐induced cardiac hypertrophy by upregulating PPARα protein levels. PPARα overexpression significantly alleviated PO‐induced cardiac hypertrophy and dysfunction in mice with Lgr6 deficiency. Our study further underscores the role and mechanism of PPARα in pathological cardiac hypertrophy. In the future, strategies to upregulate or activate PPARα for the treatment of cardiac hypertrophy may have significant clinical translation potential.

In this study, we found that Lgr6 overexpression promoted the deubiquitination and stabilization of PPARα in the cardiomyocytes of PO mice. Furthermore, Lgr6 upregulated PPARα protein levels in a USP4‐dependent manner, as revealed by IP‐MS and RNAseq analysis. Deubiquitinases have been reported to bind to PPARs and regulate disease progression. For instance, USP22 promotes liver cancer progression by directly binding and deubiquitinating PPARγ, while USP28 regulates myocardial mitochondrial homeostasis in diabetic mice by directly binding and deubiquitinating PPARα, thereby alleviating cardiac dysfunction.^[^ ^26^ ^]^ Here, we identified another deubiquitinase, USP4, that can bind and stabilize PPARα. Cardiomyocyte‐specific overexpression of USP4 effectively alleviated PO‐induced cardiac hypertrophy and dysfunction in mice with Lgr6 deficiency. These findings suggest that deubiquitinases play a crucial role in regulating energy metabolism.

Additionally, we found that Lgr6 promoted USP4 expression by activating the cGMP/PKG/CREB1 signaling pathway. As one of the classic downstream signaling pathways of GPCR, the cGMP/PKG signaling pathway plays an important role in the occurrence and progression of pathological cardiac hypertrophy. Moreover, we found that Maresin1 exerts its effects by activating Lgr6. Knockdown of Lgr6 effectively abolished the protective effect of Maresin1 on cardiac hypertrophy. Our previous studies have shown that Lgr6 overexpression can effectively alleviate diabetic cardiomyopathy and myocardial ischemia‐reperfusion injury.^[^ ^16^, ^19^ ^]^ This study further suggests the important role of Lgr6 in cardiac hypertrophy, indicating that Lgr6 may be an important target for regulating cardiovascular diseases. In conclusion, these results comprehensively illustrate the role and mechanism of cardiomyocyte Lgr6 in the development of cardiac hypertrophy.

There are still some limitations in this study. First, genetic mice with cardiomyocyte‐specific knockout of Lgr6 may be a better choice. Second, only male mice were included in our study. Future exploration of female mice may further reveal the role of Lgr6 in cardiac hypertrophy. In addition, while this study primarily focuses on the role of the USP4/PPARα signaling pathway in Lgr6‐mediated regulation of pathological cardiac hypertrophy, we acknowledge that other potential mechanisms may exist and warrant further investigation in the future.

## 4 Experimental Section

### Animals and Treatments

All experimental procedures were approved by the Animal Care and Use Committee of Renmin Hospital of Wuhan University (approval No. 20 181 215) and were conducted in accordance with the Guidelines for the Care and Use of Laboratory Animals published by the US National Institutes of Health. Wild‐type C57BL/6 mice were purchased from GemPharmatech Co., Ltd. (Nanjing, China). All animals were housed at a constant room temperature on a 12:12 h light‒dark cycle at the Animal Center of Wuhan University Renmin Hospital and were provided a standard rodent diet and water. To avoid the effects of oestrogen and the menstrual cycle, only male animals were used in the study. Transverse aortic constriction (TAC) was induced to construct a mouse model of cardiac hypertrophy. TAC or sham surgery was performed as described in a previous study.^[^ ^17^ ^]^ Male mice aged 10–12 weeks were anaesthetized via intraperitoneal injections of 3% pentobarbital sodium (60 mg kg^−1^, once) before TAC or sham surgery, and the body temperature was maintained at 37±1 °C with a warming pad. This following five in vivo experiments were performed in this study. At the end of the study, the mice were euthanized via cervical dislocation under deep anaesthesia with 3% pentobarbital sodium or 2.5% isoflurane.

1. To investigate the role of Lgr6 deficiency in cardiac hypertrophy, C57 mice received a single intravenous injection of AAV9‐cTnT‐shLgr6 at a dose of 1×10^11^ viral genomes per mouse 4 weeks prior to TAC surgery. AAV9‐cTnT‐null was used as a control. The mice were then divided into the following four groups: sham+AAV9‐cTnT‐null, sham+AAV9‐cTnT‐shLgr6, TAC+AAV9‐cTnT‐null, and TAC+AAV9‐cTnT‐shLgr6 (n = 10).
2. To specifically overexpress Lgr6 in cardiomyocytes, C57 mice received a single intravenous injection of AAV9‐cTnT‐Lgr6 at a dose of 1×10^11^ viral genomes per mouse 4 weeks prior to TAC surgery. AAV9‐cTnT‐null was used as a control. The mice were then divided into the following four groups: sham+AAV9‐cTnT‐null, sham+AAV9‐cTnT‐Lgr6, TAC+AAV9‐cTnT‐null, and TAC+AAV9‐cTnT‐Lgr6 (n = 10).
3. To specifically overexpress PPARα in cardiomyocytes, C57 mice with Lgr6 deficiency received single intravenous injection of AAV9‐cTnT‐PPARα at a dose of 1×10^11^ viral genomes per mouse 4 weeks prior to TAC surgery. AAV9‐null was used as a control. The mice were then divided into the following four groups: TAC+AAV9‐cTnT‐null, TAC+AAV9‐cTnT‐PPARα, TAC+AAV9‐cTnT‐shLgr6, and TAC+AAV9‐cTnT‐shLgr6+AAV9‐cTnT‐PPARα (n = 10).
4. To specifically overexpress USP4 in cardiomyocytes, C57 mice received a single intravenous injection of AAV9‐cTnT‐USP4 at a dose of 1×10^11^ viral genomes per mouse 4 weeks prior to TAC surgery. AAV9‐cTnT‐null was used as a control. The mice were then divided into the following four groups: TAC+AAV9‐cTnT‐null, TAC+AAV9‐cTnT‐USP4, TAC+AAV9‐cTnT‐shLgr6, and TAC+AAV9‐cTnT‐shLgr6+AAV9‐cTnT‐USP4 (n = 10).
5. To investigate the therapeutic effect of Maresin1 on PO‐induced cardiac hypertrophy, C57 mice were intraperitoneally injected with Maresin1 (2 µg/mouse/day, 4 weeks) four weeks after TAC surgery.^[^ ^16a^ ^]^

### Human Heart Tissue Collection

All experiments involving human heart samples conformed to the principles outlined in the Declaration of Helsinki (Version. 2020) and were approved by the Renmin Hospital of Wuhan University Review Board. Patients with end‐stage heart failure attributed to dilated cardiomyopathy were included. Left ventricular (LV) tissue was obtained after heart transplantation, and unmatched LV tissue from healthy donors was also collected for molecular analysis in this experiment as described previously.^[^ ^18^ ^]^ Informed consent was signed by all the donors and their families.

### Statistical Analysis

All the data are presented in scatter dot plots or line charts as means ± SDs, and p < 0.05 was considered significant. For comparisons between two groups, a two‐tailed unpaired t test was used as indicated. For more than two groups, one‐way ANOVA followed by Bonferroni's multiple comparisons test was used, and for experiments with a second variable, two‐way ANOVA followed by Bonferroni's multiple comparisons test was performed. Statistical analysis was performed with GraphPad Prism 8.0 (GraphPad Software, San Diego, CA, USA).

The supplementary methods include details about the reagents used, histological analysis, echocardiography, cell and treatment, isolation of adult mouse cardiomyocytes, adenovirus infection, siRNA transfection, immunofluorescence staining, western blotting, quantitative real‐time PCR, IP‐MS, RNA sequencing (RNA‐seq) analysis, chromatin immunoprecipitation (ChIP)‐seq datasets, the ChIP assay, the luciferase reporter assay, and CoIP.^[^ ^16^, ^17^, ^18^, ^19^ ^]^

## Conflict of Interest

The authors declare no conflict of interest.

## Author Contributions

M.Z., J.L., S.P., These authors contributed equally to this work. MZ, JW, MW, and YX were responsible for the experimental design and wrote the manuscript. JL contributed to the western blots and qPCR. ZZ contributed to the animal models. SL contributed to the graphical abstract. SP contributed to the acquisition and analysis of the data. MZ contributed to the cell culture and in vitro experiments. YX reviewed this manuscript.

## Supporting information



Supporting Information

## Data Availability

The data that support the findings of this study are available on request from the corresponding author. The data are not publicly available due to privacy or ethical restrictions.
