# Supplementary material for: Leucine‐Rich Repeat‐Containing G Protein‐Coupled Receptor 6 Ameliorates Pressure Overload‐Induced Cardiac Hypertrophy by Regulating Cardiomyocyte Metabolic Reprogramming
Source: Adv Sci (Weinh). 2025 Apr 11;12(23):2417597. doi: 10.1002/advs.202417597 (PMC12199328; doi:10.1002/advs.202417597)
Supplement: Supplementary file 1 — Supporting Information [file ADVS-12-2417597-s001.docx]

**Leucine-rich repeat-containing G protein-coupled receptor 6 ameliorates pressure overload-induced cardiac hypertrophy by regulating cardiomyocyte metabolic reprogramming**

**Zhao et al.**

**Address correspondence to:**

Dr. Menglong Wang, Department of Cardiology, Renmin Hospital of Wuhan University, 238 Jiefang Road, Wuhan 430060, China.

E-mail address: whuwangmenglong@163.com

**This file includes:**

**Supplemental Methods**

**Supplemental Figure 1-19**

**Supplemental Table 1-3**

1. **Supplemental Methods**

**1.1 Reagents**

Hematoxylin and Eosin Staining Kit (#C0105M), Masson Staining Kit (#C0189S), and Lipo6000™ transfection reagent (#C0526) were purchased from Beyotime (Shanghai, China). Pierce™ BCA protein assay kit (#23225), Alexa Fluor™ 488 conjugated Wheat Germ Agglutinin (WGA, #W11261) and SlowFade™ gold antifade reagent with DAPI (#S36939) were purchased from Invitrogen (Carlsbad, CA, USA). Sonication ChIP Kit (#RK20258), HRP-conjugated Goat anti-Rabbit IgG (#AS014) and HRP-conjugated Goat anti-Mouse IgG (AS003) were purchased from Abclonal (Shanghai, China). AAV9 encoding the mouse shLgr6 gene under the cTnT promoter (AAV9-cTnT-shLGR6) was purchased from Shanghai Genechem (Shanghai, China). AAV9-cTnT-LGR6, AAV9-cTnT-USP4 and AAV9-cTnT-PPARα were constructed by DesignGene Biotechnology (Shanghai, China). Small interfering RNA against LGR6, PPARα, USP4, and Creb1 were all obtained from Sangon Biotechnology (Shanghai, China). All plasmids were purchased from DesignGene Biotechnology (Shanghai, China). All the information of primary antibodies (name, company, cat no., molecular weight) was shown in Table S2.

**1.2 Cardiac Hypertrophy Assessment**

At 4 weeks after TAC or sham operation, the ratio of heart weight to body weight (HW/BW) of mice was detected. Mouse cardiomyocyte area was assessed by HE or wheat germ agglutinin (WGA) staining^18^. The expression levels of atrial natriuretic peptide (Anp), brain natriuretic peptide (Bnp) and beta major histocompatibility complex (Myh7) were detected by qPCR.

**1.3 Cardiac Fibrosis Assessment**

At 4 weeks after TAC or sham surgery, cardiac fibrosis was evaluated by masson staining^18^. The protein expressions of collagen 1 (Col1), and α-smooth muscle actin (α-Sma) was detected by western blots.

**1.4 Echocardiography**

Echocardiography was evaluated 4 weeks after TAC or sham surgery^20^. Systolic cardiac function in anesthetized mice was measured noninvasively by transthoracic echocardiography 15 minutes before sacrifice as described. Mice were anesthetized with isoflurane using 1.5% isoflurane, and the ventricular septum, chamber dimensions, systolic function, and ejection fraction were recorded by a MyLab 30 CV ultrasound system (Esaote S.P. A, Genoa, Italy).

**1.5 Cells and treatments**

HL1 and AC16 cells were purchased from Procell Life Science&Technology Co.,Ltd. (Wuhan, China). Primary neonatal rat cardiomyocytes (NRCMs) were isolated from 1-2-day-old Sprague-Dawley rats following the method described^19^. The cells were cultured in Dulbecco's modified Eagle's medium (DMEM, Gibco, USA) containing 10% foetal bovine serum (FBS, Cellmax, China; SA301.02), 100 U/ml penicillin and 100 μg/mL streptomycin (PS, Gibco, USA). The cells were incubated at 37 °C with 5% CO_2_. Subsequently, the cardiomyocytes were transitioned to a serum-free culture medium for 12 hours prior to stimulation with 50μM phenylephrine (PE) (PHR1017, Sigma) or an equivalent volume of phosphate-buffered saline (PBS) for 24 hours. In the case of cardiomyocytes treated with a PKG inhibitor, 1μM KT5823 (TargetMol, USA; T15670) was co-administered with PE stimulation. In the case of cardiomyocytes treated with maresin1, 100 nM maresin1 (TargetMol, USA; T38047) was administered with PE stimulation. Mechanical stretching experiments were also employed to simulate pressure overload in vitro. NRCMs were subjected to either cyclic stretching (10% elongation at 1 Hz) for 24 hours or static conditions (FX500T/C flexcell system).

**1.6 Isolation of adult mice cardiomyocytes**

The adult mouse cardiomyocytes were isolated as previously described^17^.

**1.7 Adenovirus infection and siRNA transfection**

Small interfering RNA were transfected to cells using Lipo-6000 reagent (Beyotime, China) according to the manufacturer’s instructions, and scramble RNA (siNC) was used as the negative control^17^. Total protein was extracted to determine the effectiveness of siRNA transfection by western blot. To overexpress Lgr6, PPARA, and USP4, NRCMs were infected with adenovirus (DesignGene Biotechnology, China) at a multiplicity of infection of 50 for 12 hours and Ad-null was used as a control. Target protein was detected by western blot to examine the overexpression efficiency of adenovirus. Lipofectamine™ 3000 Transfection Reagent (ThermoFisher Scientific, L3000008) was used for transfection of plasmids into HEK293T cells.

**1.8 Immunofluorescence staining**

Immunofluorescence staining was performed on heart and cell samples^21^. For immunofluorescence staining of cardiac slices, paraffin-embedded sections underwent deparaffinization, rehydration and antigen retrieval with citric acid buffer (pH = 6.0). For immunofluorescence staining of cell coverslips, cells were fixed with 4% paraformaldehyde for 15 min and permeabilized in 1% Triton X-100 for 5 min at room temperature. After blocking the non-specific binding with 10% goat serum, cardiac slices or cell coverslips were incubated with the primary antibodies at 4 °C overnight, and stained with the goat anti-rabbit IgG Alexa Fluor 568 secondary antibodies (1:200 dilution) at 37 °C for an additional 1 h. The nuclei were visualized with SlowFade™ gold antifade reagent with DAPI, and immunofluorescent images were obtained by a DP74 fluorescence microscope (OLYMPUS, Tokyo, Japan).

**1.9 Western blot**

Total proteins were extracted using RIPA lysis buffer containing a protease inhibitor cocktail and phosphatase inhibitors, and the concentration was quantified using a commercial kit. An equal number of proteins were separated by sodium dodecyl sulfate-polyacrylamide gel electrophoresis, transferred to polyvinylidene fluoride membranes, blocked with 5% skim milk and incubated with the primary antibodies (Table S2) at 4 °C overnight. On the next day, the membranes were probed with HRP-conjugated secondary antibodies at room temperature for 1 h and visualized using ChemiDoc™ XRS+ System (Bio-Rad Laboratories, Inc.) with the electrochemiluminescence reagent. Protein bands were analyzed using Image Lab Software (version 6.0, Bio-Rad Laboratories, Inc.) and normalized to internal controls.

**1.10 Quantitative real-time PCR**

PCR was performed as previously reported. Primers for target genes are listed in Table S3.

**1.11 RNA-seq analysis**

Sequencing service was provided by BioyiBiotechnology Co.,Ltd.Wuhan, China^19^. For heatmaps, leading edge gene sets from selected enriched pathways were extracted, annotated with their respective TPM values, which were then converted into raw Z-scores and imported into GraphPad Prism 9.0 for heatmap generation.

**1.12 Chromatin immunoprecipitation sequencing (chip-seq) datasets**

ChIP-seq datasets of CREB1 (GSM1537599) was searched from GEO Datasets (https://www.ncbi.nlm.nih.gov/gds/).

**1.13 ChIP assays**

Nuclear protein fractions of NRCMs were first separated by a commercial kit^17^. Then, the nuclear protein was used for CHIP assay with a commercial kit as suggested. Chromatin was sheared into 200-500bp DNA fragments by ultrasound with Biorupter (Diagenode, UCD-200), and it was pulled down with the CREB1 antibodies. The DNA-protein complexes were then eluted from the beads with elution buffer at room temperature. After reverse cross-linking and proteinase K digestion, the complexes were purified by DNA purification kit. Finally, qPCR was used to detect these purified DNA fragments and the primers of USP4 were used for qPCR assay.

**1.14 Luciferase reporter assay**

Transfection for dual-luciferase reporter plasmids was performed as described above^17^. Luciferase activity was measured using the Dual-Luciferase Reporter Assay System (Promega, E1910) according to the manufacturer’s instructions.

**1.15 Coimmunoprecipitation (CoIP)**

The CoIP test was performed according to manufacturer's protocol (Protein A/G Magnetic Beads, Biolinkedin)^18^. Briefly, the cells were lysed in octyl-d-glucoside (ODG, 2%) buffer. The protein concentration was diluted into 1 μg/μl for IP. The lysates were incubated with 2 μl primary antibodies overnight, then added 30 μl protein A + G magnetic beads for 4-6 hours. A magnetic rack was used to discard the supernatant and the beads were washed 3 times with RIPA buffer. A 30 μl 1×loading buffer was added and boiled for 10 min, then the supernatant was collected and analyzed by western blotting.

**1.16 cGMP and PKG activity assays**

The cardiac tissues and NRCMs were first collected. PKG activity and cGMP levvel were then examined according to manufacturer's protocol.

**1.17 Measurement of cellular oxygen consumption rate and glycolytic proton efflux rate**

Mitochondrial respiratory capacity was determined by measuring the oxygen consumption rate (OCR) in real time using a Seahorse XFe 24 extracellular flux analyzer (Seahorse Biosciences, Agilent Technologies, USA). Briefly, NRCMs were seeded in an assay microplate at 10^5 cells/well with 10% DMEM. After specific stimulation, the culture medium was replaced with Seahorse XF DMEM buffer (containing 10 mM glucose, 2 mM glutamine, and 1 mM pyruvate) and the plate was incubated at 37 ℃ in a CO2-free incubator for 1 h. The plate was then sequentially injected with the following compounds: oligomycin (ATP synthase inhibitor, 1.5 mM); FCCP (mitochondrial uncoupler, 2 mM); Rot/AA (complex III and complex I inhibitors, 0.5 mM; #103015-100, XF Cell Mito Stress Test Kit, Agilent Technologies, USA). Respiratory parameters including basal respiration, ATP production-coupled respiration, maximal respiration, and spare respiratory capacity were quantified in realtime by calculating the average respiratory rate and subtracting the rates before and after compound injection.

For the glycoPER assay, Rot/AA (1.5 mM) and the glycolysis inhibitor 2-deoxyglucose (2-DG, 100 mM) were sequentially added to the wells from the reagent ports at the corresponding time points. The extracellular acidification rate (ECAR) was measured in units of mpH/min. Glycolytic rate parameters were calculated according to the manufacturer's protocol for the Glycolytic Rate Assay. Basal glycolysis was determined as the glyco-PER value before the injection of rotenone/antimycin A. Basal proton efflux rate (PER) was calculated as the PER value before the injection of rotenone/antimycin A. The percentage of glycolysis-derived PER was calculated as the ratio of basal glycolysis to basal PER, multiplied by 100. Compensatory glycolysis was assessed as the glyco-PER induced by rotenone/antimycin A. Post-2-DG acidification was measured as the PER value after the injection of 2-DG.

**1.18 Transmission electron microscopy analysis**

Transmission electron microscopy (TEM) was used to observe changes in the morphology and structure of mitochondria. After euthanasia, hearts were harvested from indicated mice. 1.0 mm^3^ heart pieces cut from the left ventricular wall were fixed in 1.25 % glutaraldehyde and 0.1 M sodium cacodylate buffer at 4°C overnight. Heart samples were then washed 3 times in 0.1M sodium cacodylate, 10 minutes each time. Heart samples were cut into 90 nm thick sections after dehydration and embedding. Finally, a Hitachi HT7800 transmission electron microscope (Hitachi, Japan) was used to capture images of the mitochondria.

**1.19 Determination of lactate, NADH/NAD⁺ ratio, ATP, free fatty acids and triglycerides level**

Lactate levels were evaluated in cardiac tissues and NRCMs by using the colorimetric lactate assay kit (#S0204S; Beyotime, Shanghai, China). NADH/NAD⁺ ratio was evaluated in cardiac tissues and NRCMs by using the assay kit (#S0175; Beyotime, Shanghai, China). Intracellular and cardiac ATP production were evaluated by using an ATP measurement kit (S0027, Beyotime, China). Intracellular and cardiac free fatty acids (FFA) and triglycerides (TG) accumulation were detected by using the assay kit (#S0215S, S0219S; Beyotime, Shanghai, China).

**1.20 Lipidomic analysis**

Heart ventricle tissues were collected from TAC+AAV9-null and TAC+AAV9-Lgr6 mice and rapidly frozen in liquid nitrogen. For sample preparation, 100 μg of tissue sample and 480 μL of extraction solution (MTBE:methanol = 5:1) were transferred into an EP tube. The samples were vortexed for 30 seconds, sonicated for 10 minutes on ice, and then incubated at -40℃ for 1 hour. Subsequently, the samples were centrifuged at 3000 rpm for 15 minutes at 4℃. Finally, 75 μL of the supernatant was transferred to a new glass vial for LC/MS analysis. The LC-MS/MS analyses were performed using an ultra-high-performance liquid chromatography (UHPLC) system (1290, Agilent Technologies) equipped with a Kinetex C18 column (2.1 × 100 mm, 1.7 μm, Phenomenex). The mobile phase consisted of two components: Mobile phase A (60% acetonitrile, 40% water, and 10 mmol/L ammonium formate) and Mobile phase B (10% acetonitrile, 90% isopropanol, with 50 mL of 10 mmol/L ammonium formate added to 1000 mL of Mobile phase B). The elution gradient was as follows: 0-1.0 min, 40% B; 1.0-12.0 min, 40%-100% B; 12.0-13.5 min, 100% B; 13.5-13.7 min, 100%-40% B; 13.7-18.0 min, 40% B. The autosampler temperature was maintained at 4℃, and the injection volume was 2 μL. The MS/MS spectra were acquired using a Q Exactive mass spectrometer controlled by Xcalibur software (version 4.0.27, Thermo Fisher Scientific). The raw data files were converted to mzXML format using the 'msconvert' program from ProteoWizard. Lipid identification was performed by spectral matching against the LipidBlast library.

1. **Supplemental Figure S1-19**

**Figure S1: Cardiomyocyte LGR6 was downregulated in TAC mice.**

1. Principal component analysis (PCA) and volcano plot of gene expression in hearts from mice with sham or TAC surgery (n=3).
2. GSEA analysis for GO G protein-coupled receptor activity.
3. Single cell dataset (GSE271946) of left ventricular tissues from sham and TAC mice was analyzed. LGR6 was found to be widely expressed in the heart, with the highest expression in cardiomyocytes.
4. The expression of LGR6 in cardiomyocytes was significantly reduced in TAC mice as shown by single cell dataset (GSE271946).

**
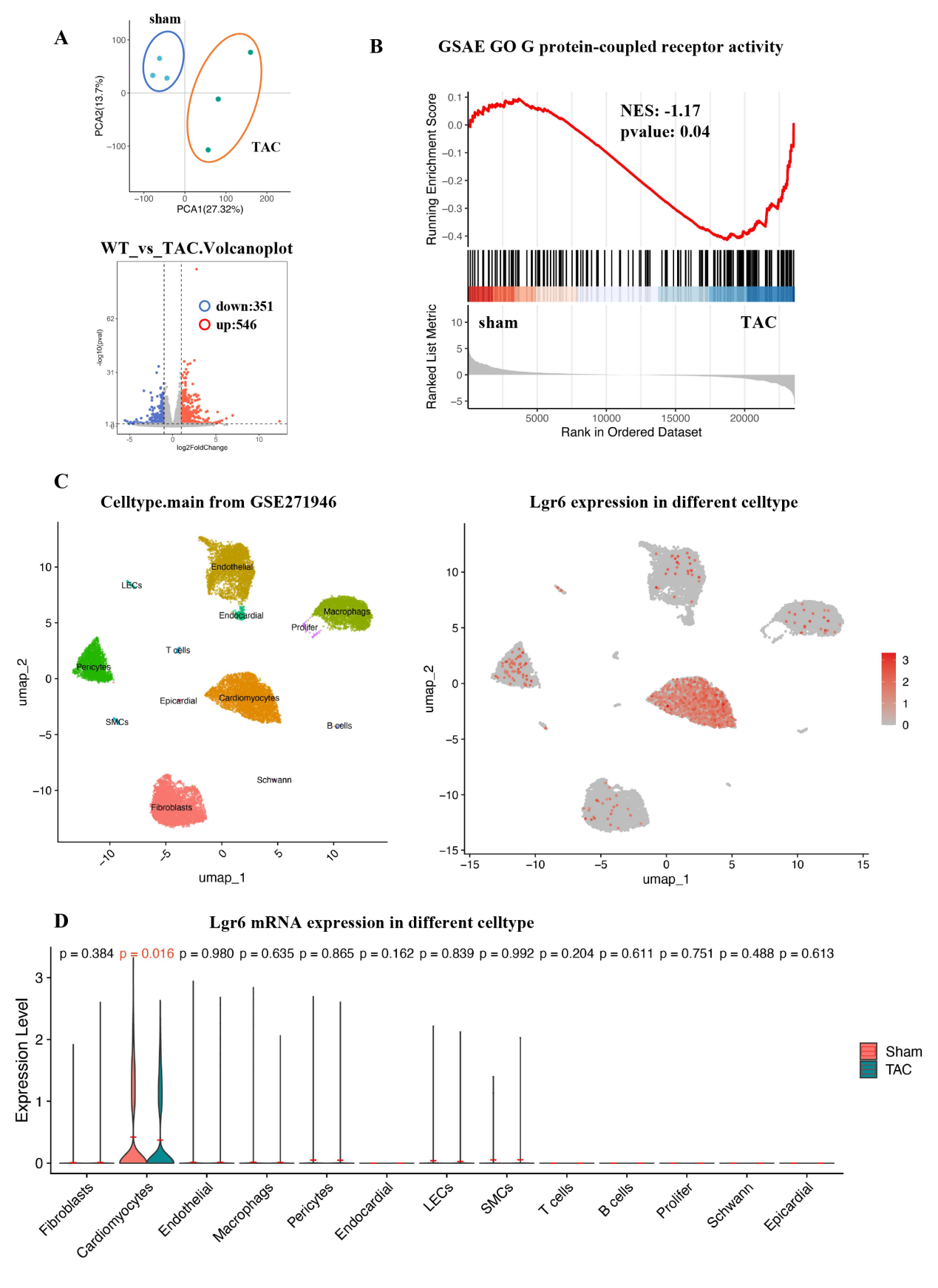
**

**Figure S2: Cyclic stretching inhibited the expression of Lgr6 in NRCMs**

(A) mRNA levels of Lgr6 and Nppa in NRCMs (n=6).

(B) Representative immunoblots and corresponding quantification showing Lgr6 and Anp in NRCMs (n=6).


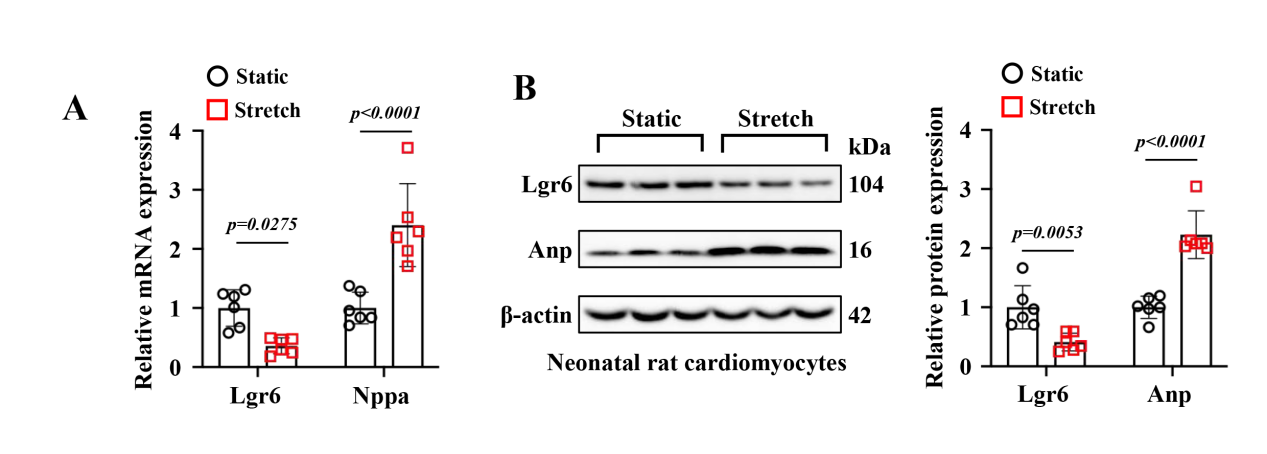


**Figure S3:**

1. Schematic diagram depicting the experimental strategy for AAV9 treatment.

(B) Representative immunoblotting images and corresponding quantification for cardiomyocyte Lgr6 expression four weeks after AAV9 injection (n=6).

(C) Representative immunoblotting images and corresponding quantification for fibroblast Lgr6 expression four weeks after AAV9 injection (n=6).

(D) Representative M-mode echocardiographic images of the left ventricle and corresponding parameters showing cardiac function four weeks after AAV9 injection (n=6).

(E) Representative images of H&E-stained sections four weeks after AAV9 injection (n=6).

(F) Heart weight (mg) and body weight (g) ratio (HW/BW) four weeks after AAV9 injection (n=6).

(G) Real-time quantitative reverse transcription polymerase chain reaction analysis comparing Nppa and Nppb mRNA expression four weeks after AAV9 injection (n=6).


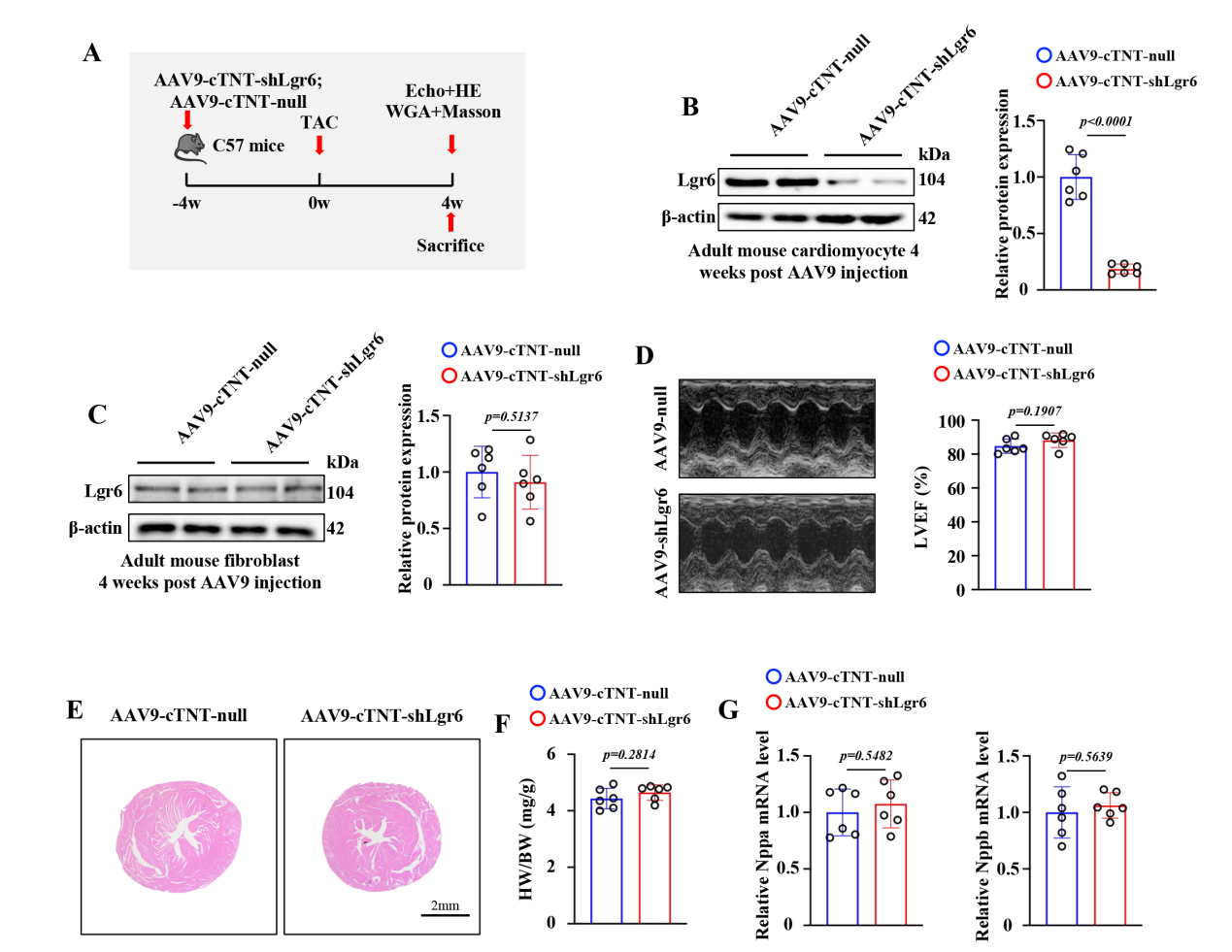


**Figure S4: Lgr6 deficiency accelerates PE-induced hypertrophy in NRCMs.**

1. Schematic diagram depicting the experimental strategy for siRNA treatment. Representative immunoblotting images and corresponding quantification for Lgr6 expression in NRCMs (n=6).
2. Representative phalloidin staining images and corresponding quantification showing cell size of NRCMs (n=10).
3. Real-time quantitative reverse transcription polymerase chain reaction analysis comparing Nppa, Nppb and Myh7 mRNA expression (n=6).
4. Representative immunoblots and corresponding quantification showing the expression of Anp and Bnp (n=6).


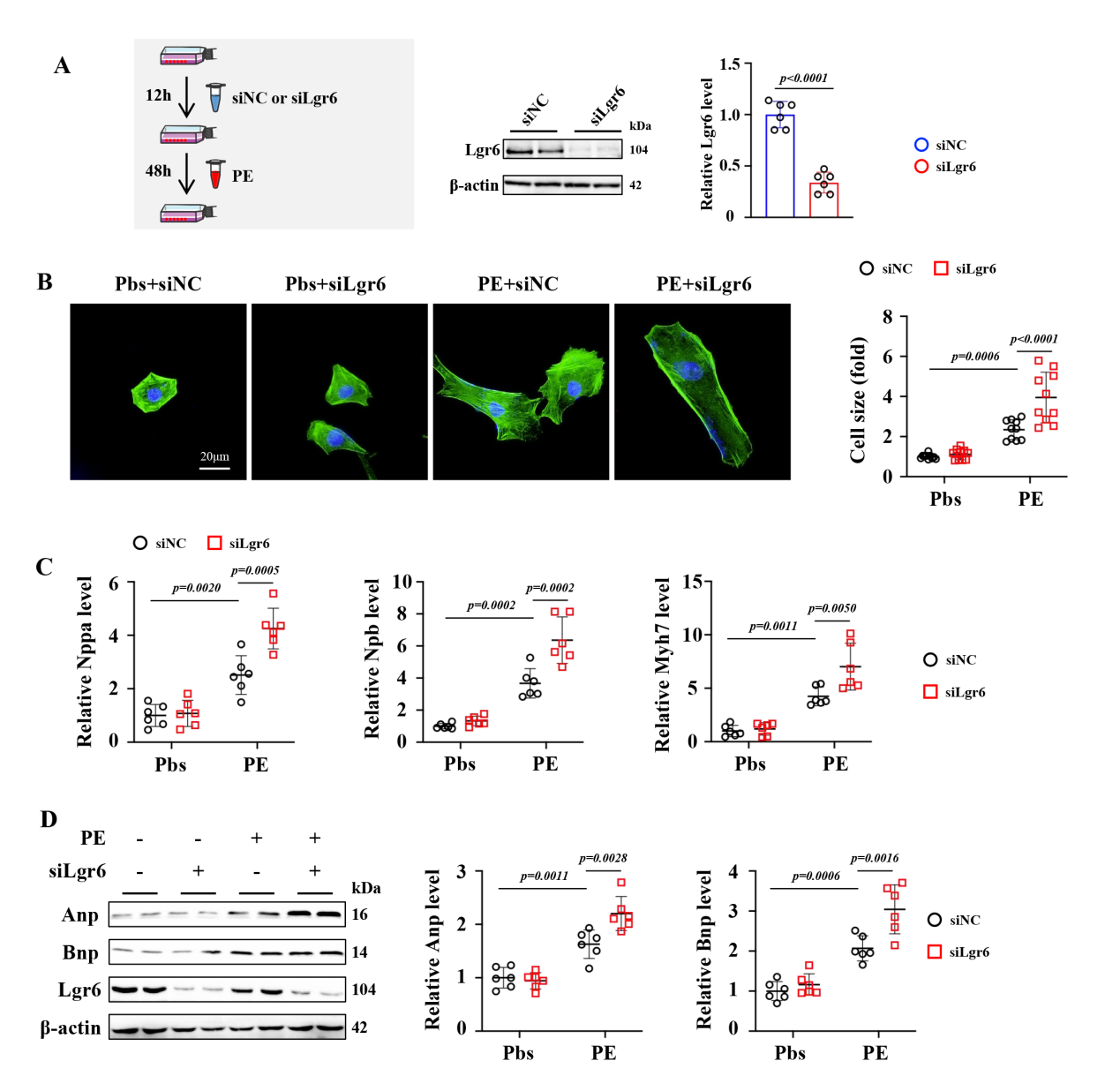


**Figure S5: Lgr6 deficiency accelerates cyclic stretching-induced hypertrophy in NRCMs.**

1. Schematic diagram depicting the experimental strategy for siRNA treatment.
2. Representative phalloidin staining images and corresponding quantification showing cell size of NRCMs (n=10).
3. Real-time quantitative reverse transcription polymerase chain reaction analysis comparing Nppa, Nppb and Myh7 mRNA expression (n=6).


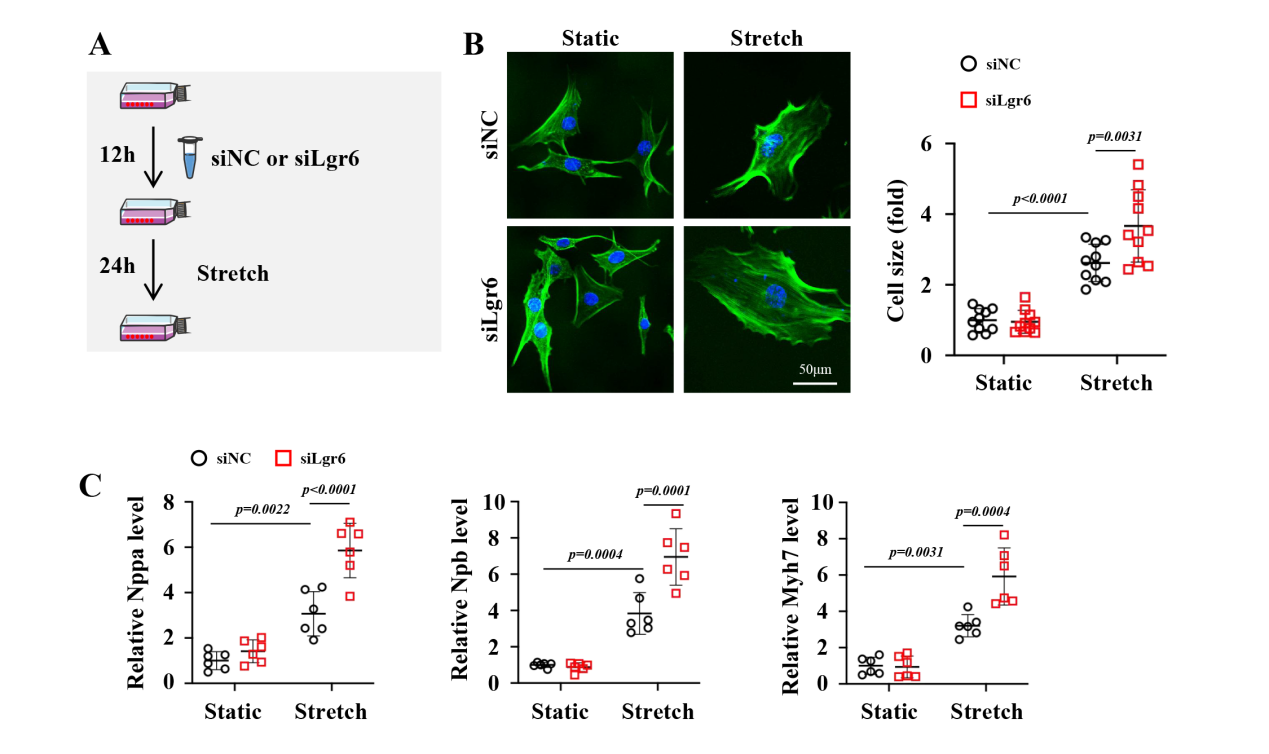


**Figure S6:**

1. Schematic diagram depicting the experimental strategy for AAV9 treatment.
2. Representative immunoblots and corresponding quantification showing the expression of cardiomyocyte Lgr6 (n=6).
3. Representative immunoblots and corresponding quantification showing the expression of fibroblast Lgr6 (n=6).


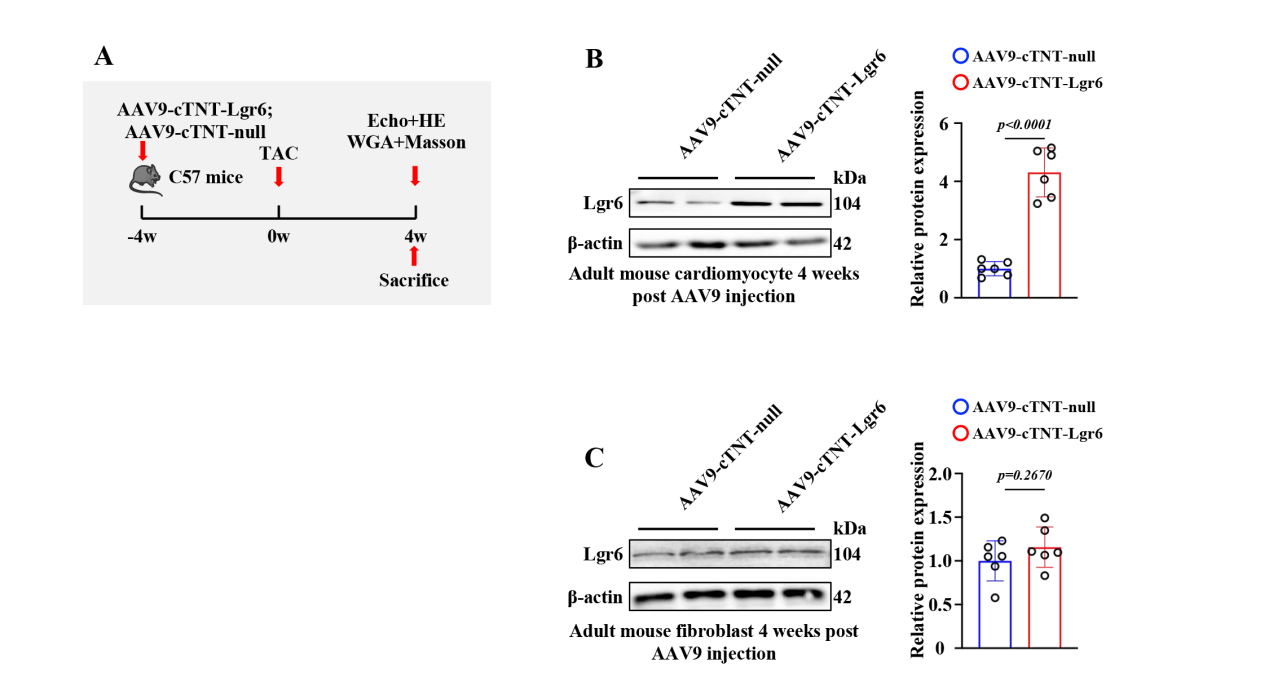


**Figure S7: Lgr6 overexpression inhibits PE-induced hypertrophy in NRCMs.**

1. Schematic diagram depicting the experimental strategy for adenovirus treatment. Representative immunoblotting images and corresponding quantification for Lgr6 expression in NRCMs (n=6).
2. Representative phalloidin staining images and corresponding quantification showing cell size of NRCMs (n=10).
3. Real-time quantitative reverse transcription polymerase chain reaction analysis comparing Nppa, Nppb and Myh7 mRNA expression (n=6).


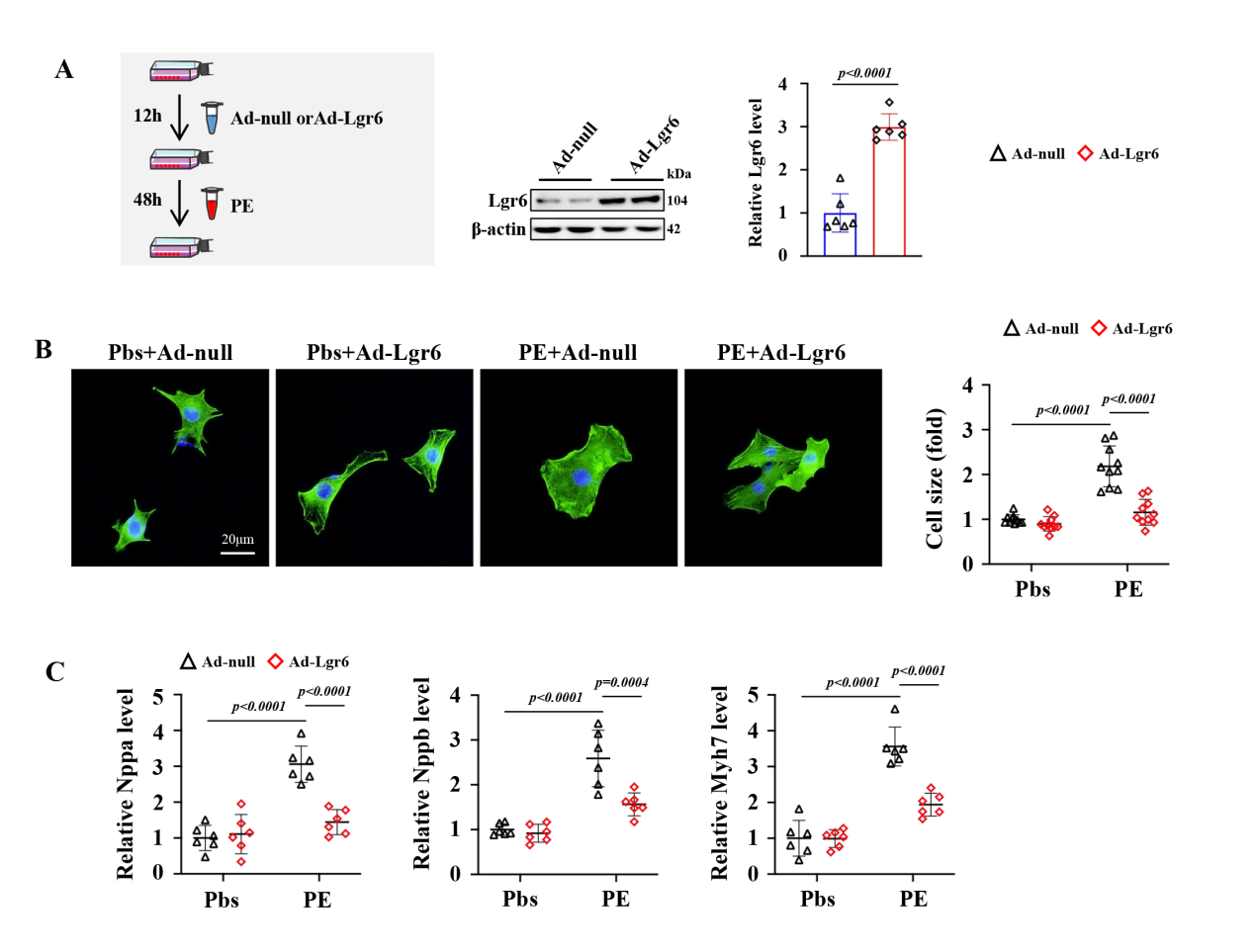


**Figure S8: Lgr6 overexpression inhibits cyclic stretching-induced hypertrophy in NRCMs.**

1. Schematic diagram depicting the experimental strategy for adenovirus treatment.
2. Representative phalloidin staining images and corresponding quantification showing cell size of NRCMs (n=10).
3. Real-time quantitative reverse transcription polymerase chain reaction analysis comparing Nppa, Nppb and Myh7 mRNA expression (n=6).

**
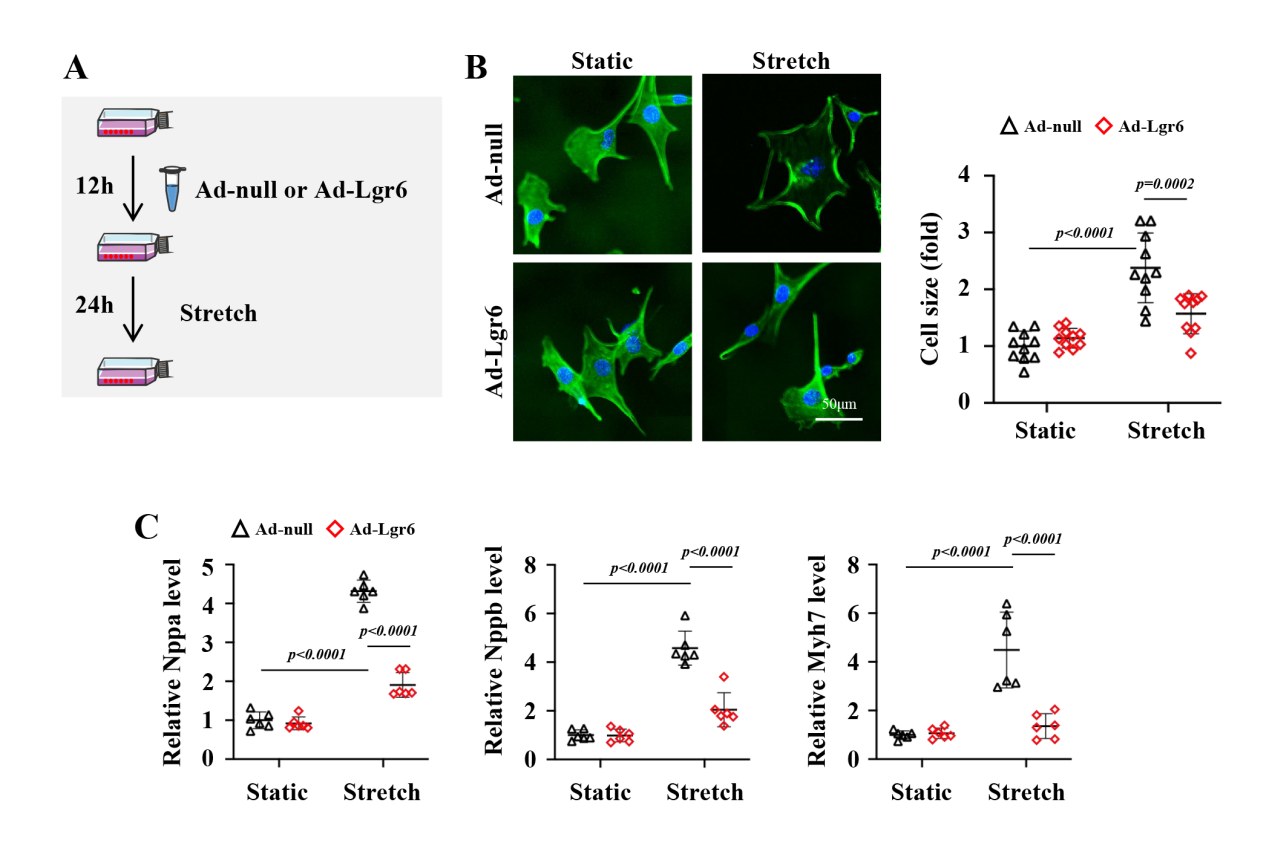
**

**Figure S9: RNAseq analysis of hearts from PO mice with AAV9-null or AAV9-Lgr6 treatment.**

1. Principal component analysis (PCA) and volcano plot of gene expression (n=3).
2. The number of the differentially expressed genes in different pathways.


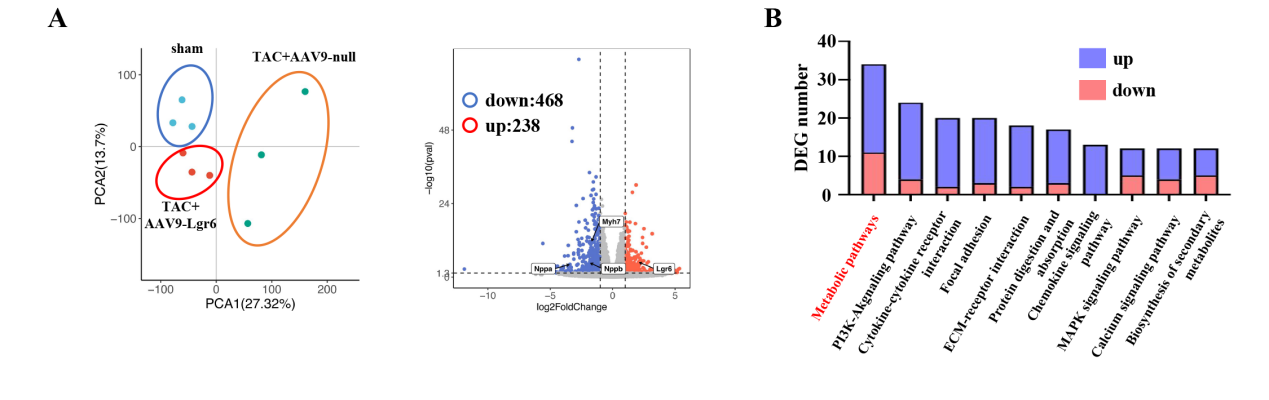


**Figure S10: Lgr6 restores the balance between glycolysis and fatty acid metabolism in PO mice**

1. Real-time quantitative reverse transcription polymerase chain reaction analysis comparing glycolysis-related genes and citrate cycle-related genes (n=6).
2. Cardiac lactate level (n=6).
3. Relative NADH/NAD⁺ ratio in cardiac tissues (n=6).
4. Cardiac ATP level (n=6).
5. Cardiac FFA level (n=6).
6. Cardiac TG level (n=6).
7. Analysis of liquid chromatography-mass spectrometry-based metabolomics on left ventricular tissue showing fatty acid metabolism (n=5).
8. Representative electron microscopy images show the damaged mitochondrial with loss of cristae in cardiomyocytes and quantification of the damaged mitochondria in hearts (n=6).

The data are shown as the mean±SD. All data were analyzed by 1-way ANOVA and Tukey’s post hoc test. & means p<0.05 vs sham+AA9-null group，# means p<0.05 vs TAC+AA9-null group.

**
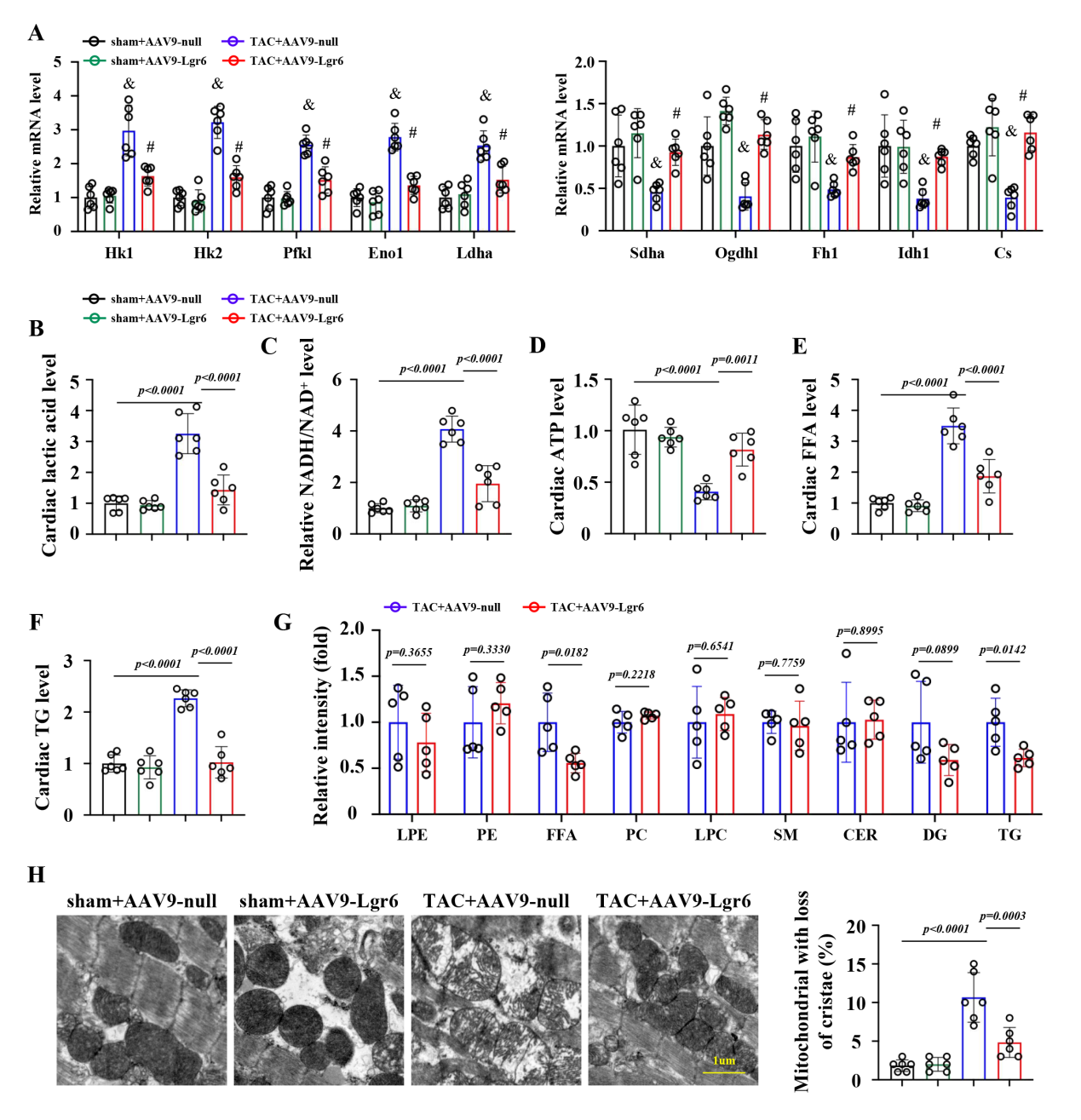
**

**Figure S11: Lgr6 restores the balance between glycolysis and fatty acid metabolism in PE-treated NRCMs**

1. Real-time quantitative reverse transcription polymerase chain reaction analysis comparing glycolysis-related genes and citrate cycle-related genes (n=6).
2. Lactate level in NRCMs (n=6).
3. Relative NADH/NAD⁺ ratio in NRCMs (n=6).
4. ATP level in NRCMs (n=6).
5. FFA level in NRCMs (n=6).
6. Representative electron microscopy images show the damaged mitochondrial with loss of cristae in cardiomyocytes and quantification of the damaged mitochondria in NRCMs (n=6).

The data are shown as the mean±SD. All data were analyzed by 1-way ANOVA and Tukey’s post hoc test. & means p<0.05 vs Pbs+Ad-null group，# means p<0.05 vs PE+Ad-null group.

**
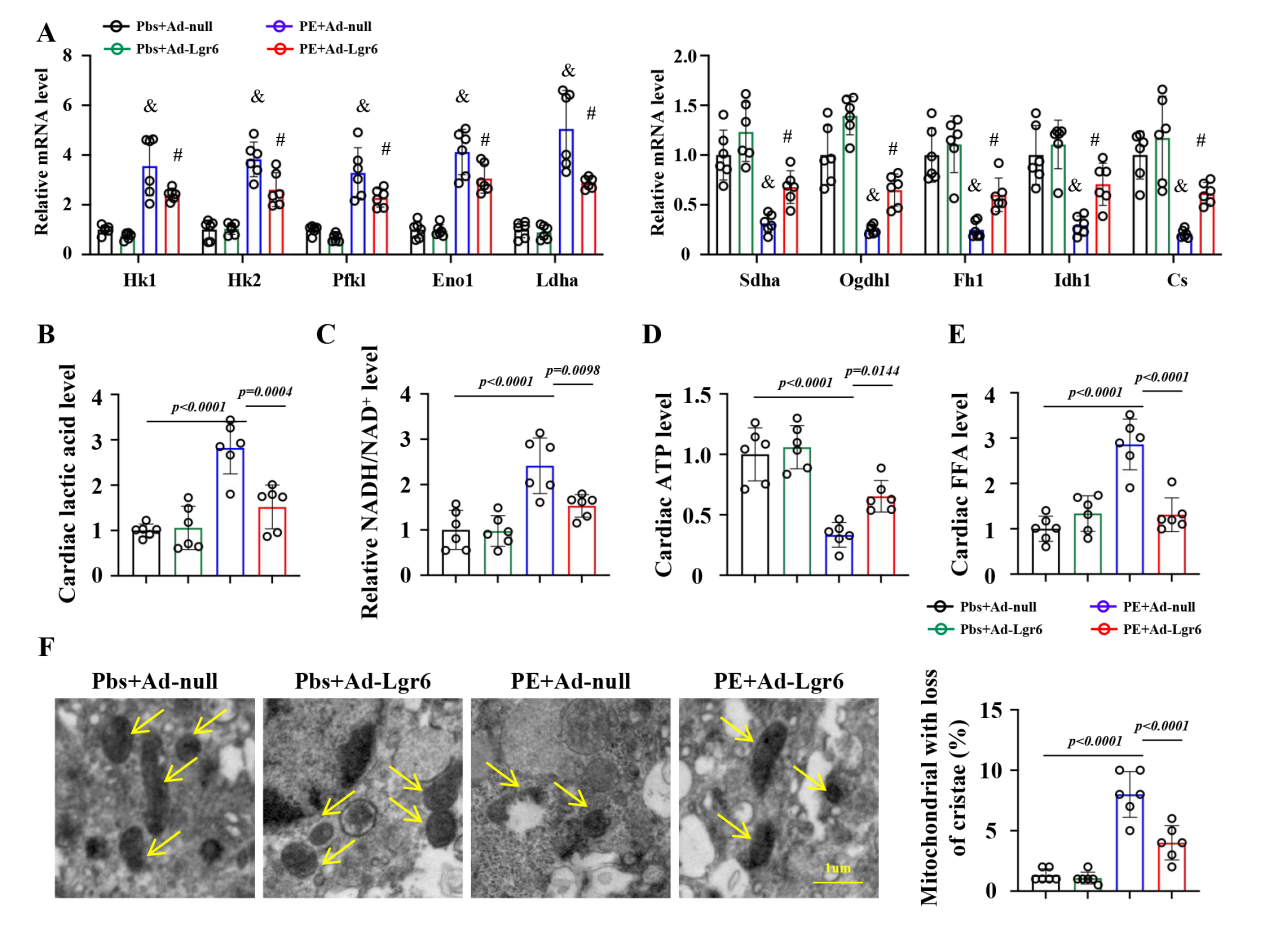
**

**Figure S12:**

1. GSEA analysis for KEGG PPAR signaling pathway.
2. Heatmaps showing genes resolved in PPAR signaling pathway (n=3).

**
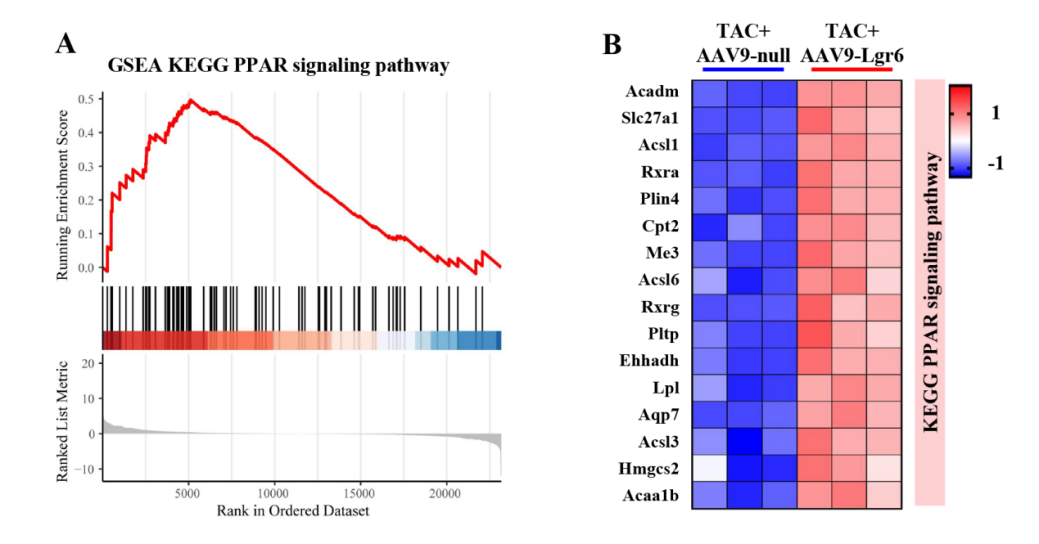
**

**Figure S13: PPARα overexpression ameliorates cardiomyocyte hypertrophy in NRCMs with LGR6 deficiency.**

1. Schematic diagram depicting the experimental strategy for siPPARα treatment. Representative immunoblots and corresponding quantification showing PPARα (n=6).
2. Representative phalloidin staining images and corresponding quantification showing cell size of NRCMs (n=10).
3. Real-time quantitative reverse transcription polymerase chain reaction analysis comparing Anp and Bnp mRNA expression (n=6).

The data are shown as the mean±SD. All data were analyzed by 1-way ANOVA and Tukey’s post hoc test.


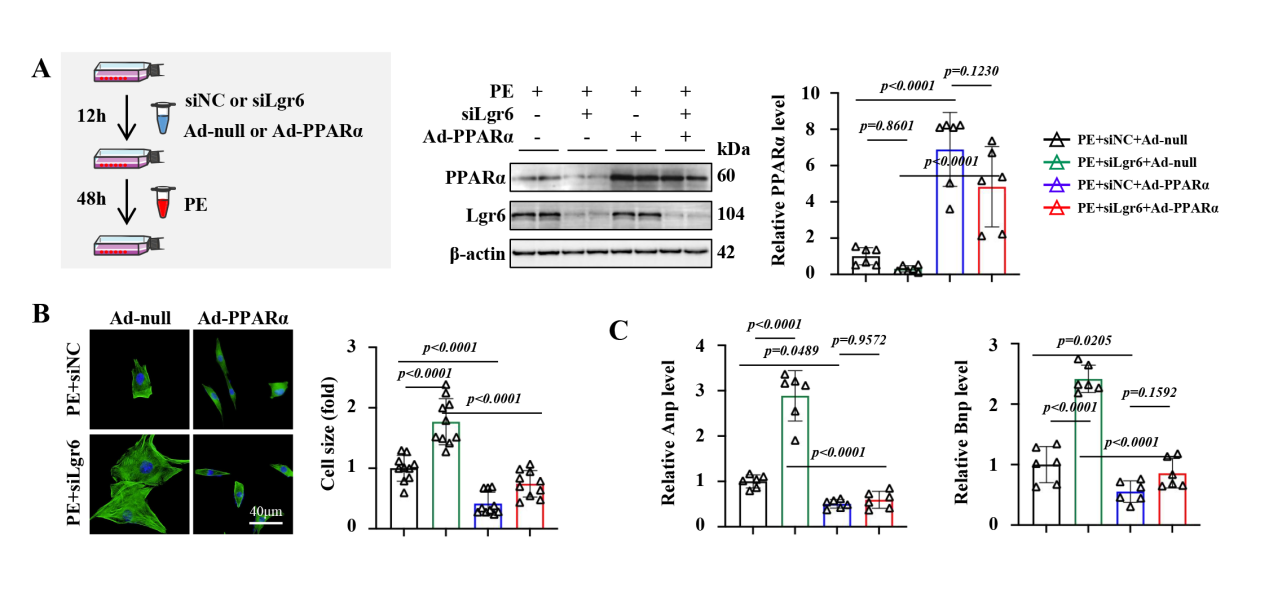


**Figure S14: PPARα overexpression ameliorates cardiac hypertrophy in PO mice with Lgr6 deficiency.**

1. Schematic diagram depicting the experimental strategy for AAV9-cTnT-PPARα and AAV9-cTnT-shLgr6 treatment.
2. Representative immunoblots showing PPARα and Lgr6 in adult mouse cardiomyocytes (n=6).
3. Representative M-mode echocardiographic images of the left ventricle and corresponding parameters showing cardiac function (n=10).
4. Representative images of H&E-stained sections (n=6).
5. Heart weight (mg) and body weight (g) ratio (HW/BW), heart weight (mg) and tibia length (mm) ratio (HW/TL) (n=10).
6. Representative images of WGA-stained sections, and quantification of the cardiomyocyte cross-sectional area based on WGA staining (n=6). Representative cardiac Masson trichrome staining in the interstitial and quantification of the LV collagen volume (n=6).

The data are shown as the mean±SD. All data were analyzed by 1-way ANOVA and Tukey’s post hoc test.


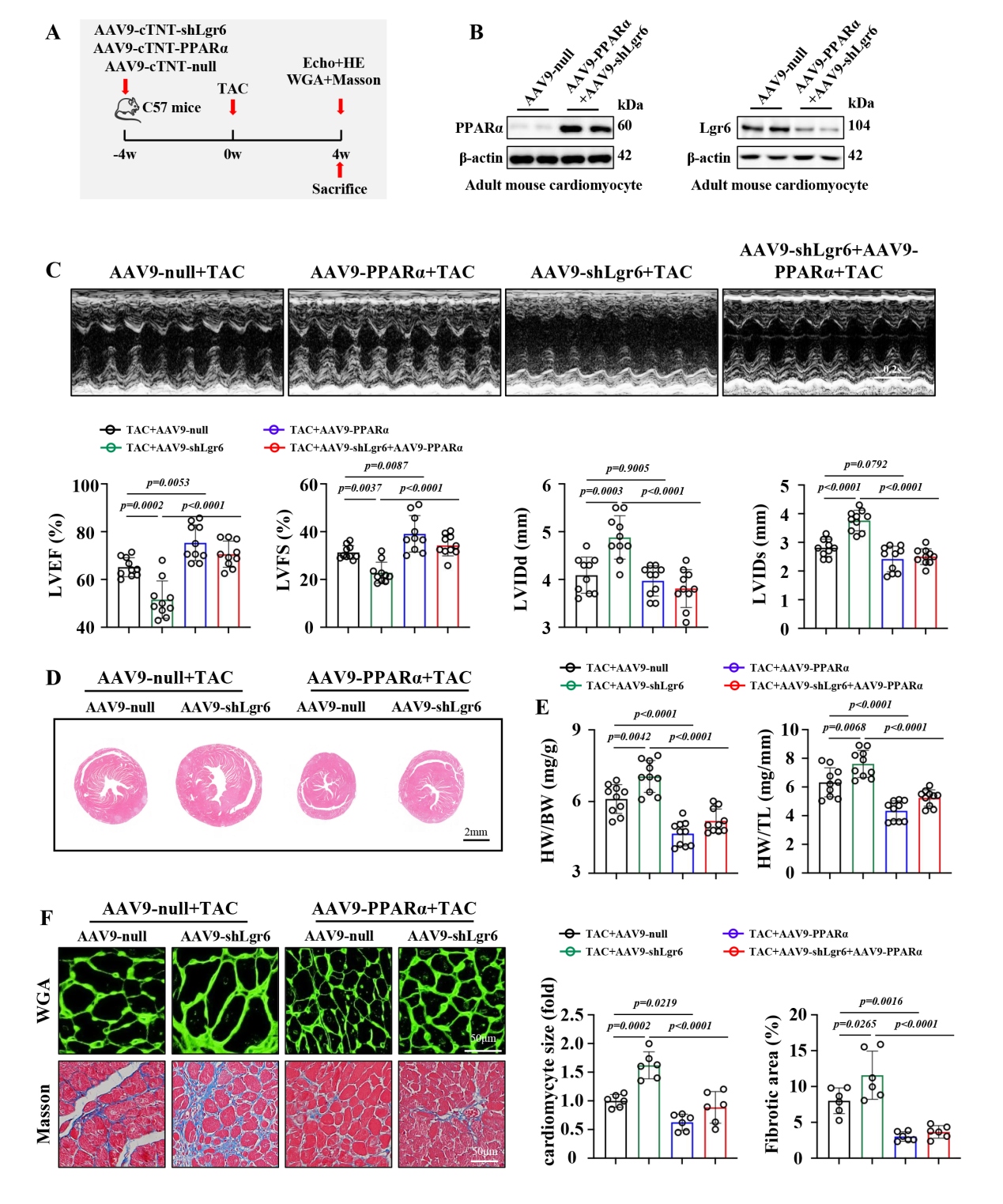


**Figure S15: Lgr6 deubiquitinates and stabilizes PPARα.**

1. Heat maps showing cardiac PPARα, PPARγ and PPARδ mRNA expression in RNAseq analysis (n=3).
2. Ubiquitination assay of PPARα in mouse hearts (n=6).
3. HEK293T cells transfected with two independent USP4 shRNA were treated with or without the proteasome inhibitor MG132 (20 μM, 8 hours) and autophagy inhibitor CQ (25 μM, 2 hours), and then USP4 and PPARα were analyzed (n=6).
4. Flag-tagged full-length or truncated PPARα were co-expressed with Myc-tagged full-length USP4 in HEK293T cells. Ubiquitination assay of Flag-PPARα was performed (n=6).
5. Flag-tagged full-length PPARα were co-expressed with Myc-tagged full-length or truncated USP4 in HEK293T cells. Ubiquitination assay of Flag-PPARα was performed (n=6).
6. HEK293T cells overexpressing USP4 were transfected with the indicated plasmid combinations to measure K6/K11/K27/K29/K33/K48/K63-linked ubiquitination of PPARα (n=6).
7. The ubiquitination sites in the DBD of PPARα were predicted by BDM-PUB and UBPRED.
8. HEK293T cells were transfected with the indicated plasmid combinations to measure the ubiquitination of PPARα (n=6).


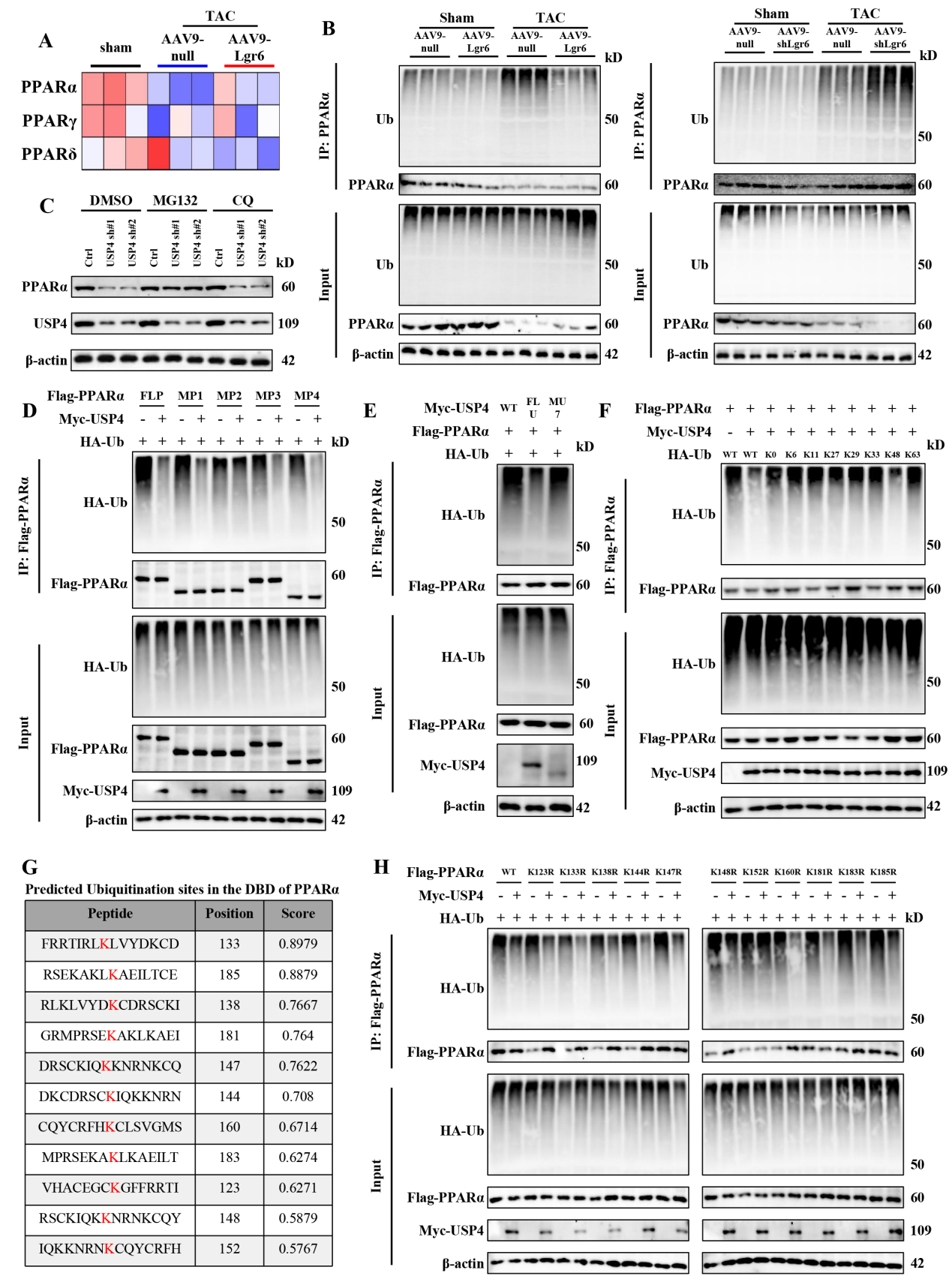


**Figure S16: Lgr6 regulates PE-induced hypertrophy in NRCMs in a USP4-dependent manner.**

1. Representative immunoblots and corresponding quantification showing the expression of USP4 (n=6).
2. Representative immunoblots and corresponding quantification showing the expression of USP4 (n=6).
3. Schematic diagram depicting the experimental strategy for siUSP4 treatment.
4. Representative immunoblots and corresponding quantification showing the expression of PPARα and Anp (n=6).
5. Schematic diagram depicting the experimental strategy for Ad-USP4 treatment.
6. Representative immunoblots and corresponding quantification showing the expression of PPARα and Anp (n=6).


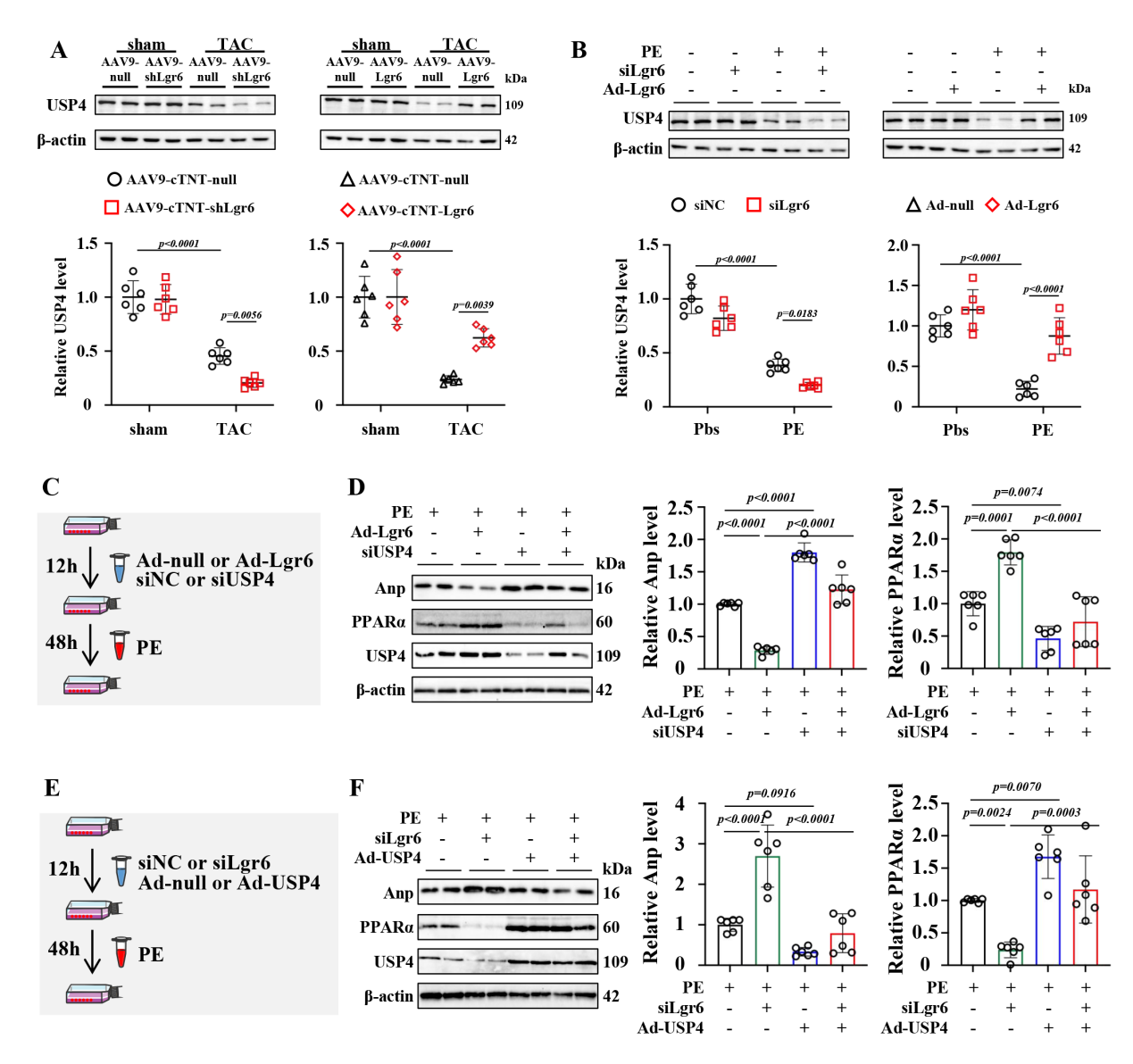


**Figure S17: USP4 overexpression ameliorates cardiac hypertrophy in PO mice with Lgr6 deficiency.**

1. Schematic diagram depicting the experimental strategy for AAV9-cTNT-USP4 treatment.
2. Representative immunoblots showing cardiac USP4 (n=6).
3. Representative M-mode echocardiographic images of the left ventricle and corresponding parameters showing cardiac function (n=10).
4. Representative images of H&E-stained sections (n=6).
5. Heart weight (mg) and body weight (g) ratio (HW/BW), heart weight (mg) and tibia length (mm) ratio (HW/TL) (n=10).
6. Representative images of WGA-stained sections, and quantification of the cardiomyocyte cross-sectional area based on WGA staining (n=6). Representative cardiac Masson trichrome staining in the interstitial and quantification of the LV collagen volume (n=6).
7. Representative immunoblots and corresponding quantification showing the expression of PPARα and Anp (n=6).

The data are shown as the mean±SD. All data were analyzed by 1-way ANOVA and Tukey’s post hoc test.


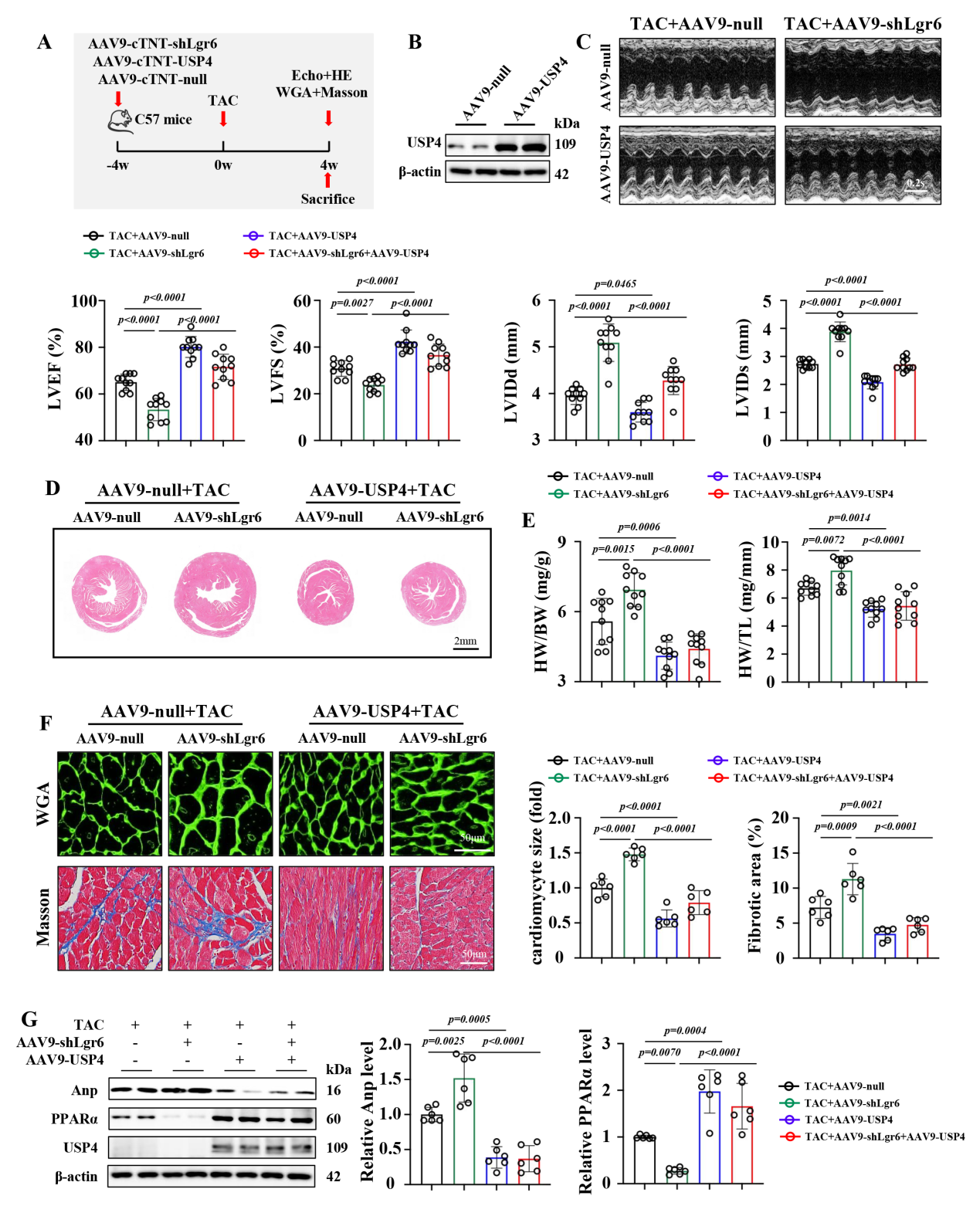


**Figure S18:** Treatment with the PKG antagonist KT5823 effectively inhibited the expression of p-Vasp and USP4 in NRCMs under PE and Lgr6 overexpression conditions (n=6). The data are shown as the mean±SD. All data were analyzed by 1-way ANOVA and Tukey’s post hoc test.


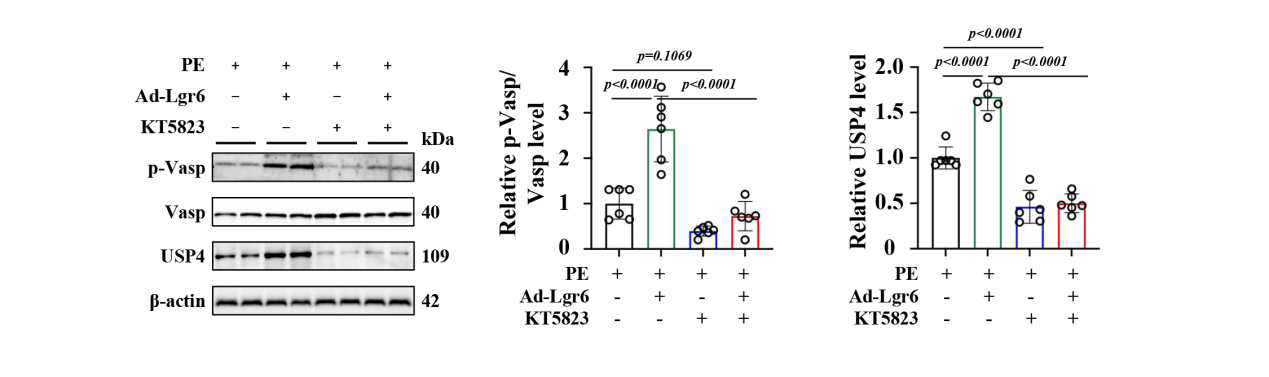


**Figure S19: Maresin1 protects mice from PO-induced cardiac hypertrophy.**

1. Schematic diagram depicting the experimental strategy for maresin1 treatment.
2. Representative M-mode echocardiographic images of the left ventricle and corresponding parameters showing cardiac function (n=6).
3. Representative images of H&E-stained sections (n=6).
4. Heart weight (mg) and body weight (g) ratio (HW/BW), heart weight (mg) and tibia length (mm) ratio (HW/TL) (n=10).
5. Representative images of WGA-stained sections, and quantification of the cardiomyocyte cross-sectional area based on WGA staining (n=6).
6. Representative cardiac Masson trichrome staining in the interstitial and quantification of the LV collagen volume (n=6).
7. Representative immunoblots and corresponding quantification showing the expression of p-Vasp, p-Creb1, USP4 and PPARα (n=6).
8. Ubiquitination assay of PPARα in mouse hearts (n=6).

The data are shown as the mean±SD. All data were analyzed by 2-way ANOVA and Tukey’s post hoc test. * means p<0.05 TAC+vehicle vs sham+vehicle. # means p<0.05 TAC+Maresin1 vs TAC+vehicle.


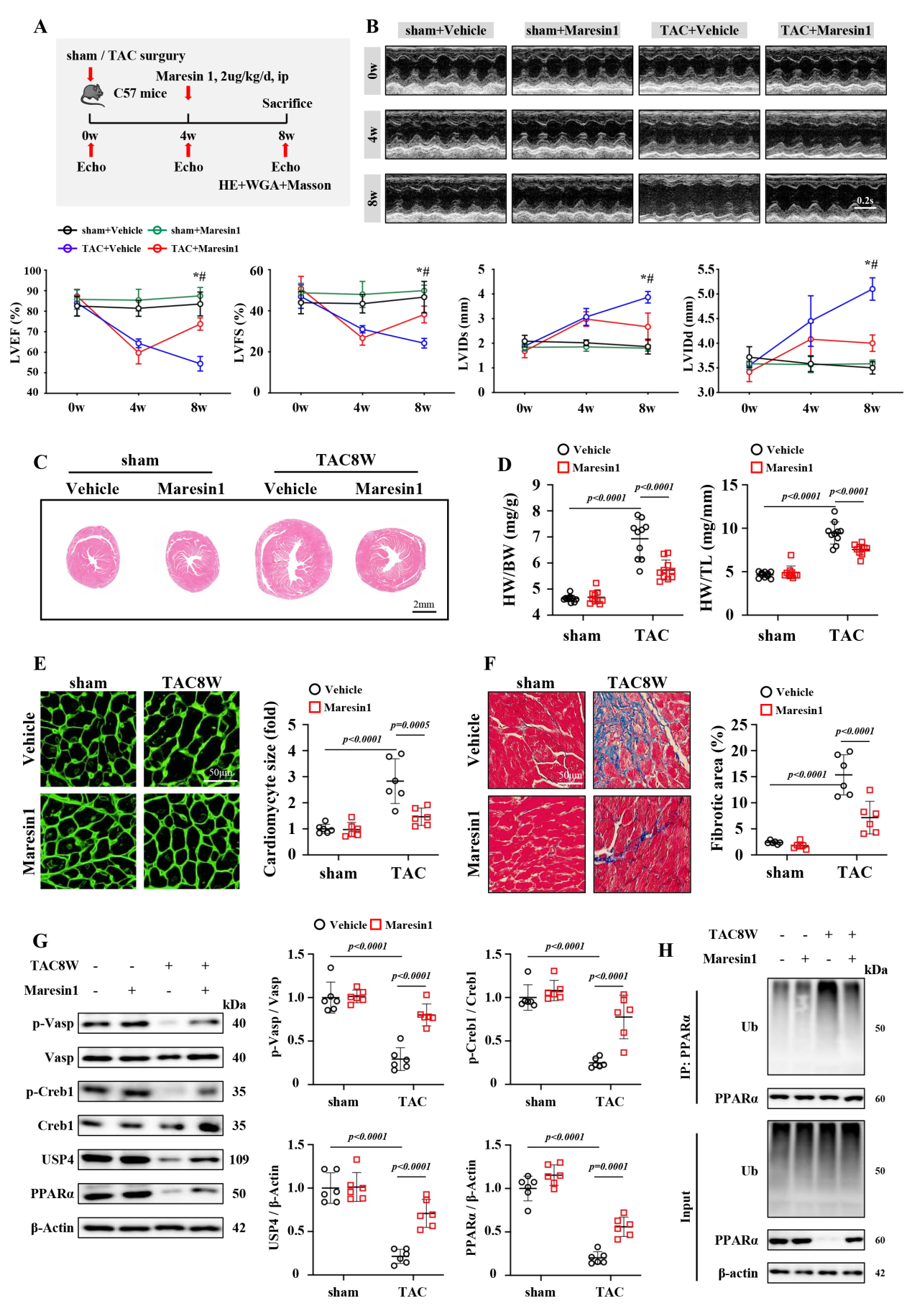


1. **Supplemental Table 1-3**

**Table S1: 478 Potential binding proteins for Flag-PPARα.**

| **Gene names** | **Peptides** | | | **Unique peptides** | | | **Sequence coverage [%]** | | | **Mol. weight [kDa]** |
| --- | --- | --- | --- | --- | --- | --- | --- | --- | --- | --- |
|  | **Flag-PPARa#1** | **Flag-PPARa#2** | **Ig G** | **Flag-PPARa#1** | **Flag-PPARa#2** | **Ig G** | **Flag-PPARa#1** | **Flag-PPARa#2** | **Ig G** |  |
| BCLAF1 | 64 | 67 | 49 | 7 | 7 | 6 | 53.8 | 56.3 | 42.4 | 106.12 |
| PPARA | 22 | 23 | 1 | 16 | 16 | 1 | 55.8 | 55.8 | 5.1 | 52.225 |
| POLDIP3 | 24 | 25 | 21 | 13 | 13 | 12 | 60.6 | 61.8 | 57 | 46.089 |
| HNRNPU | 32 | 30 | 20 | 12 | 10 | 8 | 45.7 | 44.7 | 30.1 | 90.583 |
| RPS2 | 15 | 15 | 12 | 10 | 10 | 7 | 53.9 | 53.9 | 44.7 | 31.324 |
| EIF4A3 | 28 | 28 | 21 | 23 | 23 | 18 | 75.4 | 75.4 | 50.6 | 46.871 |
| NCL | 24 | 24 | 14 | 24 | 24 | 14 | 32.5 | 32.5 | 22.5 | 74.739 |
| GAPDH | 16 | 17 | 11 | 16 | 17 | 11 | 77 | 79.4 | 41.8 | 36.053 |
| RPL3 | 20 | 20 | 11 | 20 | 20 | 11 | 52.9 | 52.9 | 31.5 | 46.108 |
| SLC25A5 | 13 | 14 | 8 | 6 | 7 | 3 | 40.6 | 40.6 | 26.8 | 32.852 |
| RPS9 | 10 | 11 | 9 | 10 | 11 | 9 | 40.2 | 36.6 | 35.6 | 22.591 |
| RPLP1 | 4 | 4 | 1 | 3 | 3 | 0 | 80.5 | 80.5 | 14.2 | 11.399 |
| RPS8 | 10 | 10 | 6 | 10 | 10 | 6 | 50 | 54.8 | 33.2 | 24.205 |
| RPL4 | 16 | 17 | 8 | 16 | 17 | 8 | 38.8 | 39.5 | 20 | 48.995 |
| RPL31 | 6 | 5 | 6 | 6 | 5 | 6 | 44.8 | 39.2 | 44.8 | 14.495 |
| RPL7A | 16 | 16 | 6 | 16 | 16 | 6 | 53.4 | 53.4 | 18.8 | 29.995 |
| RPLP0 | 11 | 11 | 9 | 11 | 11 | 9 | 47.6 | 47.6 | 40.7 | 34.273 |
| RPS16 | 13 | 14 | 9 | 13 | 14 | 9 | 65.1 | 70.5 | 51.4 | 16.445 |
| RPL8 | 10 | 11 | 5 | 10 | 11 | 5 | 52.9 | 53.3 | 19.5 | 28.024 |
| RPL18 | 6 | 6 | 4 | 6 | 6 | 4 | 39.6 | 39.6 | 26.2 | 18.756 |
| RPL7 | 12 | 12 | 6 | 12 | 12 | 6 | 49.2 | 49.2 | 28.6 | 29.225 |
| RPLP2 | 8 | 7 | 5 | 8 | 7 | 5 | 80.9 | 77.4 | 69.6 | 11.665 |
| RPL23A | 8 | 8 | 6 | 8 | 8 | 6 | 43 | 43 | 41.1 | 17.692 |
| RPL10A | 10 | 10 | 3 | 10 | 10 | 3 | 44.7 | 44.7 | 17.1 | 24.831 |
| RPS4X | 13 | 13 | 12 | 13 | 13 | 12 | 58 | 54.7 | 52.3 | 27.259 |
| ACIN1 | 29 | 30 | 0 | 25 | 26 | 0 | 25 | 27.4 | 0 | 145.44 |
| RPS24 | 6 | 6 | 5 | 6 | 6 | 5 | 41.2 | 41.2 | 40.5 | 15.197 |
| RPL13 | 8 | 9 | 6 | 8 | 9 | 6 | 37.4 | 41.7 | 29.9 | 24.265 |
| RPS6 | 9 | 9 | 4 | 9 | 9 | 4 | 33.3 | 33.3 | 12 | 28.708 |
| HEL-S-62p | 8 | 8 | 8 | 8 | 8 | 8 | 5.4 | 5.4 | 5.4 | 187.15 |
| PRKDC | 88 | 88 | 2 | 88 | 88 | 2 | 26.9 | 26.7 | 0.5 | 469.08 |
| SRRM2 | 48 | 48 | 3 | 48 | 48 | 3 | 23.1 | 23.3 | 1.3 | 299.61 |
| RPL32 | 6 | 6 | 3 | 6 | 6 | 3 | 44.4 | 44.4 | 20.3 | 15.616 |
| RPL35 | 5 | 5 | 2 | 5 | 5 | 2 | 33.3 | 33.3 | 16.3 | 14.551 |
| IPO4 | 8 | 8 | 1 | 8 | 8 | 1 | 10.3 | 10.3 | 0.9 | 118.72 |
| L27a;RPL27A | 5 | 5 | 4 | 5 | 5 | 4 | 39.6 | 39.6 | 30.2 | 12.015 |
| PNN | 23 | 23 | 9 | 23 | 23 | 9 | 31.1 | 31.1 | 12.6 | 81.541 |
| RPL30 | 8 | 7 | 3 | 8 | 7 | 3 | 67 | 67 | 31.3 | 12.784 |
| RPL23 | 7 | 6 | 3 | 7 | 6 | 3 | 60.4 | 53.7 | 28.4 | 14.149 |
| ACTB | 17 | 18 | 11 | 8 | 9 | 5 | 67.7 | 68 | 39.7 | 41.736 |
| RPS14 | 5 | 6 | 3 | 4 | 5 | 2 | 36.4 | 37.7 | 22.5 | 16.273 |
| RPS11 | 10 | 11 | 4 | 10 | 11 | 4 | 51.3 | 53.8 | 18.4 | 18.431 |
| CAD | 20 | 21 | 2 | 20 | 21 | 2 | 12.2 | 13.8 | 0.9 | 236.02 |
| THOC2 | 36 | 38 | 7 | 36 | 38 | 7 | 30.2 | 32.4 | 4.4 | 182.77 |
| RPL18A | 8 | 8 | 4 | 8 | 8 | 4 | 50 | 50 | 26 | 18.079 |
| RPS23 | 6 | 6 | 6 | 6 | 6 | 6 | 48.3 | 48.3 | 48.3 | 15.807 |
| PRPF8 | 56 | 58 | 9 | 56 | 58 | 9 | 31.4 | 32.7 | 5 | 268.05 |
| LARP1 | 26 | 28 | 15 | 24 | 25 | 15 | 31.1 | 33 | 17.5 | 123.51 |
| PGAM5 | 12 | 14 | 7 | 12 | 14 | 7 | 45 | 50.5 | 24.2 | 32.004 |
| RPL12 | 5 | 6 | 3 | 5 | 6 | 3 | 43 | 54.5 | 28.5 | 17.818 |
| SNRNP200 | 52 | 55 | 11 | 52 | 55 | 11 | 31 | 34.8 | 6.4 | 244.5 |
| RPL27 | 7 | 7 | 5 | 7 | 7 | 5 | 51.6 | 51.6 | 46 | 14.257 |
| HBB | 3 | 3 | 2 | 3 | 3 | 2 | 21.8 | 21.8 | 12.9 | 15.998 |
| HBA1 | 5 | 5 | 3 | 5 | 5 | 3 | 44.4 | 44.4 | 36.9 | 20.146 |
| GNB2L1 | 16 | 17 | 8 | 16 | 17 | 8 | 66.9 | 73.2 | 36 | 35.076 |
| CDC5L | 17 | 18 | 1 | 17 | 18 | 1 | 28.3 | 30.7 | 1.3 | 89.225 |
| RPL13A | 5 | 6 | 5 | 5 | 6 | 5 | 25.1 | 30.5 | 26.1 | 23.662 |
| EIF4A1 | 21 | 22 | 10 | 9 | 10 | 3 | 58.6 | 63.8 | 27.8 | 46.153 |
| RPL19 | 6 | 6 | 4 | 6 | 6 | 4 | 26 | 26 | 20.2 | 20.819 |
| RBM39 | 16 | 19 | 6 | 16 | 19 | 6 | 41.3 | 45.8 | 12.5 | 59.379 |
| UBTF | 20 | 21 | 1 | 20 | 21 | 1 | 35.9 | 38.2 | 1.3 | 72.203 |
| PRPF19 | 13 | 16 | 5 | 13 | 16 | 5 | 42.1 | 55.2 | 12.9 | 55.18 |
| CHD1 | 44 | 44 | 11 | 37 | 37 | 9 | 28.8 | 29.5 | 6.8 | 207.43 |
| RPS20 | 3 | 3 | 3 | 3 | 3 | 3 | 22.1 | 22.1 | 22.1 | 15.312 |
| RPL24 | 6 | 6 | 4 | 6 | 6 | 4 | 47.1 | 47.1 | 28.9 | 14.369 |
| PRPF6 | 12 | 11 | 0 | 12 | 11 | 0 | 14 | 12.8 | 0 | 106.92 |
| DNAJA1 | 12 | 12 | 2 | 12 | 12 | 2 | 43.6 | 43.6 | 8.1 | 44.868 |
| U5-116KD | 29 | 28 | 18 | 28 | 27 | 17 | 37.7 | 40.6 | 24.4 | 108.21 |
| HNRNPL | 19 | 21 | 12 | 19 | 21 | 12 | 60.3 | 65.6 | 33 | 61.926 |
| RPL28 | 7 | 6 | 4 | 7 | 6 | 4 | 40.9 | 33.6 | 27 | 15.747 |
| YTHDF2 | 14 | 14 | 5 | 11 | 11 | 4 | 26.5 | 26.5 | 10.7 | 61.319 |
| RPL38 | 6 | 6 | 2 | 6 | 6 | 2 | 50 | 50 | 24.3 | 8.2178 |
| RPL36 | 5 | 5 | 3 | 5 | 5 | 3 | 30.5 | 30.5 | 28.6 | 12.254 |
| RPL35A | 5 | 5 | 1 | 5 | 5 | 1 | 40.4 | 40.4 | 9.6 | 10.645 |
| BUB3 | 10 | 12 | 8 | 10 | 12 | 8 | 43.6 | 59.5 | 31 | 36.954 |
| RPL11 | 9 | 9 | 4 | 9 | 9 | 4 | 59.9 | 59.9 | 31.1 | 19.024 |
| DDX50 | 14 | 18 | 3 | 14 | 18 | 3 | 26.7 | 34.2 | 5.6 | 72.157 |
| RPL21 | 5 | 5 | 3 | 5 | 5 | 3 | 31.9 | 31.9 | 25 | 18.565 |
| RPL36A | 5 | 5 | 4 | 5 | 5 | 4 | 25 | 25 | 23.2 | 13.228 |
| XPO1 | 26 | 28 | 0 | 26 | 28 | 0 | 32.9 | 36.1 | 0 | 123.38 |
| RPS15A | 5 | 5 | 5 | 5 | 5 | 5 | 46.2 | 46.2 | 46.2 | 14.839 |
| DDX21 | 20 | 22 | 7 | 20 | 22 | 7 | 33.5 | 36.5 | 9.1 | 87.343 |
| MYBBP1A | 27 | 28 | 3 | 27 | 28 | 3 | 24.3 | 24.9 | 3 | 148.85 |
| TMEM263 | 5 | 5 | 4 | 5 | 5 | 4 | 62.9 | 62.9 | 49.1 | 11.748 |
| DDX20 | 25 | 24 | 12 | 25 | 24 | 12 | 44.2 | 42.4 | 17.5 | 92.239 |
| PCBP1 | 11 | 10 | 4 | 8 | 7 | 2 | 50.3 | 43.3 | 12.9 | 37.497 |
| TOP1 | 23 | 22 | 6 | 23 | 22 | 6 | 34.1 | 32.8 | 7.8 | 90.725 |
| RPL34 | 3 | 3 | 3 | 3 | 3 | 3 | 22.2 | 22.2 | 22.2 | 13.293 |
| PPHLN1 | 13 | 14 | 6 | 3 | 3 | 0 | 33.8 | 37.1 | 12.7 | 44.682 |
| VIM | 22 | 23 | 7 | 19 | 20 | 4 | 53 | 57.1 | 14.4 | 53.651 |
| NIFK | 12 | 12 | 4 | 12 | 12 | 4 | 49.8 | 49.8 | 12.6 | 34.222 |
| ABCF2 | 19 | 20 | 3 | 19 | 20 | 3 | 35.2 | 37.4 | 5.3 | 71.289 |
| IRS4 | 20 | 21 | 2 | 20 | 21 | 2 | 24.6 | 27.1 | 1.8 | 133.68 |
| CNBP | 8 | 8 | 3 | 8 | 8 | 3 | 52.4 | 52.4 | 17.6 | 18.742 |
| RPS18 | 9 | 8 | 5 | 9 | 8 | 5 | 52.6 | 46.7 | 28.9 | 17.718 |
| SF3B3 | 22 | 21 | 11 | 22 | 21 | 11 | 25.5 | 24.2 | 11.4 | 135.58 |
| SLC25A6 | 12 | 13 | 8 | 3 | 3 | 1 | 45.6 | 46 | 28.5 | 32.866 |
| RPL22L1 | 3 | 4 | 2 | 3 | 4 | 2 | 39.7 | 45.5 | 29.8 | 14.478 |
| ATP5A1 | 15 | 15 | 4 | 15 | 15 | 4 | 35.3 | 35.1 | 7.2 | 59.75 |
| SRPK1 | 12 | 12 | 3 | 11 | 11 | 2 | 26.4 | 26.4 | 5 | 74.324 |
| CSE1L | 19 | 18 | 1 | 19 | 18 | 1 | 25.7 | 23.6 | 0.9 | 110.42 |
| SPATS2L | 13 | 12 | 5 | 13 | 12 | 5 | 32.4 | 30.3 | 10.8 | 61.728 |
| RNPS1 | 4 | 4 | 1 | 4 | 4 | 1 | 29.9 | 29.9 | 7.1 | 24.561 |
| RPL37A | 5 | 5 | 1 | 5 | 5 | 1 | 69.1 | 69.1 | 11.8 | 7.624 |
| SNRPD3 | 4 | 4 | 3 | 4 | 4 | 3 | 37.3 | 37.3 | 20.6 | 13.916 |
| SRSF6 | 8 | 8 | 4 | 7 | 8 | 3 | 29.2 | 30.5 | 17.3 | 26.306 |
| CSDE1 | 19 | 20 | 6 | 19 | 20 | 6 | 27.4 | 28.4 | 10 | 88.884 |
| RPL5 | 10 | 9 | 2 | 10 | 9 | 2 | 45.4 | 41.2 | 10.9 | 27.045 |
| ATP2A2 | 16 | 18 | 3 | 11 | 13 | 3 | 19.3 | 23.2 | 4.1 | 114.76 |
| U2SURP | 19 | 18 | 7 | 19 | 18 | 7 | 22 | 22.5 | 8.5 | 118.29 |
| CWC22 | 13 | 12 | 1 | 13 | 12 | 1 | 17 | 14.6 | 1.1 | 105.47 |
| PARP1 | 22 | 27 | 3 | 22 | 27 | 3 | 27.4 | 35.1 | 3.1 | 113.08 |
| TUFM | 14 | 15 | 0 | 14 | 15 | 0 | 40.2 | 44.2 | 0 | 49.874 |
| PABPN1 | 6 | 5 | 3 | 6 | 5 | 3 | 25.2 | 23.9 | 11.1 | 32.749 |
| EIF3A | 29 | 30 | 13 | 29 | 30 | 13 | 23.1 | 25.3 | 11.2 | 166.47 |
| HSP90AB1 | 20 | 21 | 3 | 13 | 13 | 1 | 33.6 | 35.5 | 5 | 83.263 |
| LRPPRC | 20 | 25 | 3 | 20 | 25 | 3 | 16.8 | 20.9 | 2.6 | 157.9 |
| TNPO1 | 16 | 18 | 1 | 16 | 18 | 1 | 24.6 | 29 | 2.2 | 102.35 |
| EEF2 | 24 | 25 | 10 | 23 | 24 | 9 | 32.9 | 36 | 12 | 95.337 |
| DDX18 | 11 | 11 | 1 | 11 | 11 | 1 | 26 | 26.4 | 2.2 | 61.594 |
| ZC3H14 | 15 | 15 | 2 | 4 | 4 | 0 | 25.8 | 25.8 | 3.5 | 82.875 |
| THOC3 | 11 | 11 | 4 | 11 | 11 | 4 | 40.2 | 40.2 | 14.8 | 38.771 |
| SF3B6 | 4 | 4 | 2 | 4 | 4 | 2 | 38.4 | 38.4 | 17.6 | 14.585 |
| XRCC6 | 20 | 19 | 11 | 20 | 19 | 11 | 43.2 | 39.7 | 21.2 | 69.842 |
| KIAA1429 | 21 | 24 | 9 | 21 | 24 | 9 | 14.8 | 17.5 | 6.8 | 202.02 |
| CHD2 | 30 | 32 | 4 | 23 | 25 | 2 | 18.5 | 20.7 | 2.7 | 211.34 |
| MCM5 | 17 | 21 | 4 | 17 | 21 | 4 | 27.9 | 33 | 7.5 | 82.236 |
| VDAC2 | 10 | 10 | 1 | 10 | 10 | 1 | 42.9 | 42.9 | 2.8 | 30.348 |
| EIF3B | 16 | 17 | 7 | 16 | 17 | 7 | 24.8 | 30.7 | 10.2 | 88.679 |
| SRP14 | 4 | 4 | 3 | 4 | 4 | 3 | 34.6 | 34.6 | 27.2 | 14.57 |
| RRP1B | 17 | 16 | 3 | 17 | 16 | 3 | 28.6 | 25.9 | 5.5 | 84.427 |
| SLC25A3 | 7 | 7 | 1 | 7 | 7 | 1 | 29.6 | 27.2 | 3.7 | 36.161 |
| OTUD4 | 25 | 28 | 6 | 25 | 28 | 6 | 29.6 | 33.4 | 6.7 | 124.04 |
| HNRNPH2 | 10 | 10 | 7 | 6 | 6 | 3 | 33.6 | 33.6 | 20.7 | 49.263 |
| RSL1D1 | 10 | 12 | 1 | 10 | 12 | 1 | 23.3 | 31.2 | 3 | 48.208 |
| MCM7 | 18 | 19 | 2 | 18 | 19 | 2 | 30.7 | 32.5 | 4.5 | 81.279 |
| RPN1 | 11 | 13 | 0 | 11 | 13 | 0 | 19.9 | 24.2 | 0 | 68.606 |
| AP2B1 | 16 | 14 | 3 | 16 | 14 | 3 | 19.4 | 16.9 | 3.2 | 104.55 |
| CCT3 | 18 | 19 | 0 | 18 | 19 | 0 | 43.1 | 45.2 | 0 | 57.945 |
| PPIG | 7 | 7 | 1 | 7 | 7 | 1 | 20.1 | 20.1 | 2.2 | 51.715 |
| BCAS2 | 9 | 11 | 1 | 9 | 11 | 1 | 64.9 | 70.2 | 7.1 | 26.131 |
| SNRPN | 6 | 6 | 3 | 6 | 6 | 3 | 34.3 | 34.3 | 13.6 | 17.546 |
| RPS28 | 3 | 3 | 2 | 3 | 3 | 2 | 46.4 | 46.4 | 33.3 | 7.8409 |
| RPS10 | 5 | 5 | 4 | 5 | 5 | 4 | 29.1 | 29.1 | 20 | 18.898 |
| MCMBP | 16 | 17 | 6 | 16 | 17 | 6 | 31.1 | 32.3 | 9.4 | 72.748 |
| THRAP3 | 17 | 21 | 11 | 3 | 3 | 2 | 46.1 | 46.1 | 29 | 41.6 |
| PHGDH | 12 | 12 | 1 | 12 | 12 | 1 | 29.6 | 29.6 | 3.6 | 56.65 |
| PPIL4 | 14 | 15 | 6 | 14 | 15 | 6 | 38.4 | 42.5 | 16.7 | 57.224 |
| PCMT1 | 7 | 7 | 1 | 7 | 7 | 1 | 52.9 | 52.9 | 6.2 | 24.636 |
| CSNK2A1 | 11 | 11 | 2 | 11 | 11 | 2 | 39.6 | 41.2 | 5.6 | 45.143 |
| GCN1L1 | 36 | 39 | 2 | 36 | 39 | 2 | 16.4 | 17.7 | 1 | 292.71 |
| SF3A3 | 11 | 10 | 6 | 11 | 10 | 6 | 28.1 | 24.4 | 15.6 | 58.876 |
| GEMIN5 | 19 | 20 | 5 | 19 | 20 | 5 | 16.8 | 17.4 | 3.3 | 168.59 |
| ZC3HAV1 | 17 | 21 | 2 | 17 | 21 | 2 | 22.2 | 30.5 | 3.1 | 101.43 |
| SF3B5 | 4 | 4 | 1 | 4 | 4 | 1 | 55.8 | 55.8 | 12.8 | 10.135 |
| HEL-S-15 | 6 | 7 | 4 | 6 | 7 | 4 | 47.6 | 53 | 30.7 | 18.502 |
| NOP2 | 7 | 8 | 0 | 7 | 8 | 0 | 12.9 | 14.7 | 0 | 89.301 |
| TCP1 | 18 | 18 | 3 | 18 | 18 | 3 | 42.3 | 42.8 | 6.7 | 60.343 |
| GNL3 | 9 | 10 | 0 | 9 | 10 | 0 | 20.4 | 23.5 | 0 | 61.992 |
| SRRT | 13 | 16 | 3 | 13 | 16 | 3 | 19.4 | 25 | 4.1 | 100.67 |
| MCM3 | 16 | 18 | 1 | 16 | 18 | 1 | 19.9 | 24.4 | 1.1 | 90.98 |
| C1QBP | 5 | 7 | 2 | 5 | 7 | 2 | 25.9 | 41.8 | 8.5 | 31.38 |
| SON | 13 | 12 | 0 | 3 | 3 | 0 | 7.4 | 6.7 | 0 | 263.83 |
| RBM15 | 15 | 18 | 4 | 15 | 18 | 4 | 23.7 | 29.2 | 4.9 | 98.013 |
| RRP12 | 17 | 19 | 3 | 17 | 19 | 3 | 15.3 | 16.8 | 2.6 | 143.67 |
| HSPD1 | 12 | 13 | 6 | 12 | 13 | 6 | 32.6 | 35.3 | 13.4 | 61.054 |
| CROP | 9 | 7 | 1 | 9 | 7 | 1 | 44.9 | 35.2 | 3.4 | 27.016 |
| GRWD1 | 7 | 6 | 3 | 7 | 6 | 3 | 25.3 | 20.4 | 9.2 | 49.447 |
| SRSF1 | 10 | 10 | 5 | 9 | 9 | 4 | 42.7 | 42.7 | 19 | 28.329 |
| AIFM1 | 13 | 10 | 1 | 13 | 10 | 1 | 31 | 24.6 | 2 | 66.9 |
| PRKRA | 9 | 8 | 3 | 5 | 4 | 2 | 39 | 35.1 | 12.8 | 34.404 |
| COPB1 | 16 | 17 | 3 | 16 | 17 | 3 | 23.3 | 24.8 | 4.2 | 107.14 |
| DDX39A | 16 | 16 | 6 | 3 | 3 | 0 | 45 | 43.8 | 14.1 | 49.129 |
| SNRPD1 | 4 | 4 | 3 | 4 | 4 | 3 | 37 | 37 | 20.2 | 13.281 |
| ZNF638 | 11 | 12 | 3 | 11 | 12 | 3 | 9.6 | 10.4 | 2.3 | 175.97 |
| LYAR | 7 | 7 | 0 | 7 | 7 | 0 | 25.3 | 25.3 | 0 | 43.633 |
| CDC73 | 13 | 12 | 1 | 13 | 12 | 1 | 28.6 | 26.4 | 1.5 | 60.576 |
| L1RE1 | 7 | 7 | 6 | 7 | 7 | 6 | 22.8 | 22.8 | 20.1 | 40.055 |
| NOP14 | 11 | 10 | 1 | 11 | 10 | 1 | 15.5 | 14.4 | 1.1 | 97.669 |
| TARDBP | 8 | 8 | 2 | 8 | 8 | 2 | 32.1 | 32.1 | 8.7 | 44.739 |
| EIF2S3 | 13 | 15 | 4 | 13 | 15 | 4 | 38.3 | 42.8 | 11.9 | 51.109 |
| ZFR | 17 | 19 | 3 | 17 | 19 | 3 | 21.5 | 23.9 | 3.9 | 117.01 |
| ATP1A1 | 12 | 14 | 3 | 12 | 14 | 3 | 15.6 | 17.6 | 3.4 | 112.44 |
| ARF4 | 5 | 6 | 0 | 3 | 5 | 0 | 35.6 | 37.8 | 0 | 20.511 |
| DNAJA2 | 7 | 8 | 0 | 7 | 8 | 0 | 19.4 | 25.5 | 0 | 45.745 |
| RPS17 | 3 | 3 | 1 | 3 | 3 | 1 | 7 | 7 | 1.5 | 64.532 |
| XRCC5 | 14 | 13 | 5 | 14 | 13 | 5 | 26.4 | 26.2 | 6.7 | 82.704 |
| SLC25A11 | 9 | 9 | 0 | 9 | 9 | 0 | 34.8 | 34.8 | 0 | 32.182 |
| RPL9 | 9 | 10 | 1 | 9 | 10 | 1 | 64.6 | 65.1 | 3.6 | 21.863 |
| EMD | 7 | 8 | 1 | 7 | 8 | 1 | 29.1 | 37.4 | 5.1 | 28.994 |
| WDR57 | 8 | 9 | 2 | 8 | 9 | 2 | 28.6 | 37 | 7.3 | 39.31 |
| ADAR | 12 | 16 | 0 | 12 | 16 | 0 | 15.8 | 22.6 | 0 | 101.54 |
| PRRC2B | 19 | 22 | 6 | 15 | 19 | 5 | 11.6 | 12.5 | 3.4 | 242.96 |
| CAND1 | 12 | 13 | 0 | 12 | 13 | 0 | 12.1 | 12.9 | 0 | 136.32 |
| ZNF326 | 15 | 17 | 5 | 15 | 17 | 5 | 31.4 | 37.6 | 9.8 | 65.653 |
| C1orf57 | 6 | 8 | 1 | 6 | 8 | 1 | 46.8 | 60.5 | 5.8 | 20.713 |
| EIF2AK2 | 8 | 7 | 1 | 8 | 7 | 1 | 20.9 | 18.7 | 4.1 | 57.619 |
| PRPF40A | 9 | 9 | 0 | 9 | 9 | 0 | 12.1 | 12.1 | 0 | 104.36 |
| AP2A1 | 11 | 10 | 3 | 7 | 7 | 2 | 13.2 | 10.7 | 3.3 | 107.54 |
| SUGP2 | 5 | 8 | 0 | 5 | 8 | 0 | 8 | 12.2 | 0 | 93.859 |
| ATP5B | 10 | 10 | 2 | 10 | 10 | 2 | 32.8 | 32.8 | 6.1 | 48.113 |
| DDX41 | 14 | 15 | 3 | 14 | 15 | 3 | 35.3 | 36.1 | 6.2 | 55.409 |
| ZC3H11A | 16 | 15 | 6 | 16 | 15 | 6 | 26.7 | 25.1 | 8.3 | 82.771 |
| RBM25 | 7 | 7 | 2 | 7 | 7 | 2 | 9.8 | 9.5 | 2.3 | 100.18 |
| NOP16 | 7 | 6 | 2 | 7 | 6 | 2 | 41 | 36.5 | 10.7 | 21.188 |
| NOP58 | 10 | 11 | 4 | 10 | 11 | 4 | 25 | 27.4 | 7.8 | 59.578 |
| IPO7 | 12 | 15 | 0 | 11 | 14 | 0 | 16.4 | 21.2 | 0 | 119.52 |
| NSRP1 | 13 | 12 | 2 | 13 | 12 | 2 | 24.7 | 22.6 | 3 | 66.388 |
| RBM6 | 17 | 16 | 3 | 16 | 15 | 3 | 16.6 | 15.6 | 2.8 | 128.62 |
| GTPBP4 | 13 | 12 | 0 | 13 | 12 | 0 | 21.8 | 20.1 | 0 | 73.708 |
| SNRPA1 | 7 | 7 | 2 | 7 | 7 | 2 | 37.3 | 37.3 | 9 | 28.415 |
| UPF3B | 9 | 8 | 5 | 9 | 8 | 5 | 21.9 | 18.3 | 14.3 | 56.113 |
| BRIX1 | 5 | 4 | 1 | 5 | 4 | 1 | 19 | 14.2 | 2.8 | 41.401 |
| CMSS1 | 4 | 5 | 2 | 4 | 5 | 2 | 23.8 | 33.1 | 12.7 | 20.955 |
| NAT10 | 14 | 18 | 0 | 9 | 10 | 0 | 18 | 22.7 | 0 | 115.73 |
| CSNK2B | 5 | 5 | 0 | 5 | 5 | 0 | 30.2 | 30.2 | 0 | 24.942 |
| EIF3S3 | 9 | 11 | 4 | 9 | 11 | 4 | 30.4 | 37.2 | 15.3 | 39.93 |
| DKC1 | 10 | 11 | 1 | 10 | 11 | 1 | 23.5 | 27 | 2.5 | 57.673 |
| SMC4 | 18 | 18 | 2 | 18 | 18 | 2 | 15.8 | 16.1 | 1.8 | 144.45 |
| WDR61 | 6 | 7 | 1 | 6 | 7 | 1 | 34.4 | 37.4 | 3.4 | 32.253 |
| SLC25A1 | 7 | 7 | 1 | 7 | 7 | 1 | 23.6 | 23.6 | 2.5 | 34.787 |
| RBM7 | 8 | 8 | 0 | 8 | 8 | 0 | 41.4 | 41.4 | 0 | 30.531 |
| AP2M1 | 8 | 8 | 2 | 8 | 8 | 2 | 25.5 | 25.5 | 4.1 | 49.654 |
| FEN1 | 7 | 7 | 1 | 7 | 7 | 1 | 27.9 | 26.5 | 2.3 | 38.791 |
| ZC3H13 | 8 | 8 | 1 | 7 | 7 | 1 | 6.4 | 6.4 | 0.7 | 196.7 |
| SNRPF | 3 | 3 | 3 | 3 | 3 | 3 | 27.9 | 27.9 | 27.9 | 9.7251 |
| UNC45A | 14 | 15 | 1 | 14 | 15 | 1 | 15.9 | 17.9 | 1 | 118.39 |
| EIF3D | 6 | 5 | 1 | 6 | 5 | 1 | 12.8 | 10.2 | 2.6 | 63.972 |
| ACP1 | 5 | 6 | 1 | 5 | 6 | 1 | 37.6 | 43.6 | 6.7 | 18.698 |
| YTHDC2 | 12 | 16 | 1 | 12 | 16 | 1 | 9.2 | 13.1 | 0.8 | 160.25 |
| THOC7 | 7 | 7 | 2 | 7 | 7 | 2 | 41.2 | 41.2 | 11.3 | 23.743 |
| AQR | 12 | 11 | 1 | 12 | 11 | 1 | 14.4 | 12.3 | 1.6 | 156.84 |
| RRS1 | 5 | 4 | 0 | 5 | 4 | 0 | 19.2 | 14.5 | 0 | 41.193 |
| NOC4L | 8 | 9 | 1 | 8 | 9 | 1 | 20.2 | 26 | 2.3 | 58.467 |
| CTR9 | 11 | 11 | 1 | 11 | 11 | 1 | 11 | 10.3 | 1.2 | 133.5 |
| EBNA1BP2 | 7 | 5 | 0 | 7 | 5 | 0 | 24.5 | 17.3 | 0 | 34.852 |
| DBR1 | 8 | 9 | 1 | 8 | 9 | 1 | 19.5 | 21.5 | 1.9 | 58.5 |
| LEO1 | 4 | 4 | 1 | 4 | 4 | 1 | 6.8 | 6.8 | 2 | 72.783 |
| RBBP4 | 9 | 7 | 0 | 4 | 3 | 0 | 28 | 22.4 | 0 | 47.655 |
| SMU1 | 9 | 9 | 2 | 9 | 9 | 2 | 20.1 | 20.1 | 5.8 | 57.543 |
| DHX57 | 12 | 13 | 4 | 12 | 12 | 4 | 10.8 | 12.8 | 3.5 | 155.6 |
| NOP56 | 8 | 8 | 2 | 8 | 8 | 2 | 19.9 | 19.2 | 5.1 | 49.756 |
| SKIV2L2 | 10 | 13 | 3 | 10 | 13 | 3 | 12.9 | 16.3 | 3.3 | 117.9 |
| NOC3L | 7 | 7 | 1 | 7 | 7 | 1 | 11 | 11 | 1.4 | 92.547 |
| TRIM28 | 6 | 7 | 2 | 6 | 7 | 2 | 7.2 | 9.5 | 2.3 | 88.559 |
| RTCA | 5 | 5 | 0 | 5 | 5 | 0 | 17.5 | 17.5 | 0 | 39.336 |
| ABCD3 | 9 | 11 | 0 | 9 | 11 | 0 | 17.5 | 19.9 | 0 | 75.421 |
| RARS | 10 | 11 | 0 | 10 | 11 | 0 | 18.2 | 19.5 | 0 | 75.378 |
| HACD3 | 4 | 5 | 0 | 4 | 5 | 0 | 15.7 | 21.5 | 0 | 43.159 |
| PLRG1 | 11 | 12 | 0 | 11 | 12 | 0 | 27.6 | 31.7 | 0 | 57.193 |
| MMTAG2 | 5 | 4 | 1 | 5 | 4 | 1 | 24 | 18.6 | 4.9 | 29.411 |
| SLTM | 8 | 9 | 3 | 8 | 9 | 3 | 10.2 | 11.1 | 3.9 | 117.15 |
| EEF1G | 9 | 9 | 2 | 9 | 9 | 2 | 19 | 19 | 5.9 | 50.118 |
| KIF2A | 9 | 14 | 2 | 9 | 14 | 2 | 14.8 | 22.1 | 2.7 | 75.028 |
| PPP1CC | 14 | 15 | 10 | 3 | 3 | 1 | 47.3 | 53.7 | 41.5 | 33.773 |
| PPFIA1 | 12 | 12 | 0 | 12 | 12 | 0 | 14.6 | 15.7 | 0 | 135.78 |
| FLNA | 13 | 14 | 0 | 13 | 14 | 0 | 6.8 | 7.4 | 0 | 280.74 |
| YWHAQ | 9 | 10 | 6 | 6 | 7 | 3 | 35.1 | 42.9 | 25.3 | 27.764 |
| LBR | 4 | 5 | 1 | 4 | 5 | 1 | 11.9 | 17 | 1.9 | 46.925 |
| HNRNPH3 | 5 | 5 | 3 | 5 | 5 | 3 | 21.1 | 21.1 | 12.4 | 36.926 |
| ALDH18A1 | 10 | 11 | 0 | 10 | 11 | 0 | 15.2 | 16.7 | 0 | 87.301 |
| EIF3G | 5 | 6 | 2 | 5 | 6 | 2 | 22.8 | 23.4 | 7.2 | 35.601 |
| LARP4B | 8 | 10 | 2 | 8 | 10 | 2 | 17.9 | 21.2 | 2.9 | 75.347 |
| XPOT | 9 | 10 | 0 | 9 | 10 | 0 | 11.4 | 12.8 | 0 | 109.92 |
| SMC3 | 14 | 17 | 1 | 14 | 17 | 1 | 12.8 | 16.4 | 1 | 141.53 |
| EIF2S1 | 6 | 9 | 4 | 6 | 9 | 4 | 20.6 | 30.5 | 11.7 | 36.112 |
| RCC2 | 8 | 10 | 0 | 8 | 10 | 0 | 23.2 | 30.2 | 0 | 49.678 |
| SRSF5 | 4 | 3 | 2 | 3 | 3 | 1 | 15 | 12.3 | 7.1 | 37.254 |
| SART1 | 8 | 9 | 2 | 8 | 9 | 2 | 13.5 | 16.2 | 3 | 90.254 |
| CTNND1 | 9 | 9 | 0 | 9 | 9 | 0 | 14 | 14 | 0 | 92.387 |
| DRG1 | 10 | 12 | 1 | 10 | 12 | 1 | 34.9 | 43.1 | 3.3 | 40.542 |
| ATP5C1 | 4 | 5 | 0 | 4 | 5 | 0 | 16.8 | 29.2 | 0 | 27.512 |
| SFXN1 | 4 | 4 | 1 | 4 | 4 | 1 | 15.2 | 16.1 | 3.7 | 35.619 |
| COPB2 | 8 | 8 | 1 | 8 | 8 | 1 | 12.1 | 12.6 | 1.4 | 93.579 |
| RPL7L1 | 5 | 4 | 0 | 5 | 4 | 0 | 25.5 | 21.2 | 0 | 29.669 |
| FRG1 | 7 | 6 | 0 | 7 | 6 | 0 | 35.3 | 30.6 | 0 | 29.172 |
| COPG2 | 7 | 7 | 0 | 5 | 5 | 0 | 10.6 | 10.6 | 0 | 97.621 |
| PWP1 | 5 | 4 | 0 | 5 | 4 | 0 | 15.5 | 13.8 | 0 | 45.812 |
| NOC2L | 5 | 6 | 0 | 5 | 6 | 0 | 7.7 | 9.5 | 0 | 84.916 |
| AP2A2 | 11 | 10 | 1 | 7 | 7 | 0 | 16.1 | 13.8 | 1 | 103.96 |
| DDX55 | 7 | 6 | 1 | 7 | 6 | 1 | 25.1 | 22.1 | 2.2 | 46.089 |
| PHRF1 | 7 | 8 | 2 | 7 | 8 | 2 | 7.6 | 8.2 | 1.4 | 161.91 |
| RXRB | 11 | 11 | 0 | 11 | 11 | 0 | 25 | 25.9 | 0 | 56.921 |
| AKAP17A | 6 | 6 | 1 | 6 | 6 | 1 | 10.6 | 10.6 | 1.2 | 80.735 |
| AP3D1 | 11 | 13 | 1 | 11 | 13 | 1 | 13 | 15.4 | 0.9 | 135.42 |
| TRIP13 | 6 | 7 | 0 | 6 | 7 | 0 | 16 | 22 | 0 | 48.55 |
| COPA | 8 | 9 | 0 | 8 | 9 | 0 | 8.8 | 9.9 | 0 | 114.87 |
| SMC2 | 11 | 11 | 0 | 11 | 11 | 0 | 11 | 11.2 | 0 | 135.7 |
| USP9X | 10 | 11 | 0 | 10 | 11 | 0 | 4.1 | 4.3 | 0 | 291.02 |
| NSUN2 | 12 | 13 | 0 | 12 | 13 | 0 | 19.9 | 22.6 | 0 | 86.47 |
| DCUN1D5 | 5 | 4 | 0 | 5 | 4 | 0 | 22.4 | 18.6 | 0 | 27.508 |
| IPO5 | 9 | 9 | 0 | 8 | 8 | 0 | 11.2 | 11.2 | 0 | 123.63 |
| DPM1 | 6 | 6 | 1 | 6 | 6 | 1 | 27.7 | 28.1 | 4.2 | 29.634 |
| RBM22 | 5 | 6 | 1 | 5 | 6 | 1 | 15.7 | 18.1 | 2.4 | 46.895 |
| GOLPH3 | 4 | 5 | 1 | 3 | 4 | 0 | 17.1 | 19.9 | 4.6 | 32.054 |
| NUP205 | 12 | 13 | 0 | 12 | 13 | 0 | 8.1 | 9.2 | 0 | 227.92 |
| BAG2 | 5 | 5 | 0 | 5 | 5 | 0 | 35.5 | 35.5 | 0 | 23.772 |
| NUP188 | 12 | 13 | 0 | 12 | 13 | 0 | 8.2 | 8.8 | 0 | 196.04 |
| GNL2 | 5 | 6 | 0 | 5 | 6 | 0 | 7.5 | 8.9 | 0 | 83.654 |
| PHF6 | 6 | 7 | 1 | 6 | 7 | 1 | 27.5 | 30.2 | 4.9 | 36.43 |
| PPIL1 | 5 | 5 | 2 | 5 | 5 | 2 | 35.5 | 35.5 | 18.7 | 18.237 |
| SHINC3 | 6 | 5 | 0 | 6 | 5 | 0 | 23.9 | 19.8 | 0 | 27.951 |
| RPF2 | 4 | 5 | 0 | 4 | 5 | 0 | 18.6 | 24.2 | 0 | 35.572 |
| SMC1A | 9 | 9 | 0 | 9 | 9 | 0 | 11.1 | 11.4 | 0 | 97.946 |
| NHP2L1 | 3 | 5 | 3 | 3 | 5 | 3 | 26.6 | 53.1 | 31.2 | 14.173 |
| RFC1 | 11 | 11 | 0 | 11 | 11 | 0 | 13.2 | 13.2 | 0 | 128.25 |
| PCNA | 6 | 7 | 0 | 6 | 7 | 0 | 33 | 41.8 | 0 | 28.715 |
| FHL1 | 7 | 7 | 0 | 7 | 7 | 0 | 23.5 | 23.2 | 0 | 36.263 |
| U2AF1 | 3 | 3 | 1 | 3 | 3 | 1 | 14.2 | 14.2 | 5 | 27.873 |
| RAD50 | 11 | 11 | 0 | 11 | 11 | 0 | 9.5 | 9.5 | 0 | 142.94 |
| MSI1 | 7 | 6 | 1 | 6 | 5 | 1 | 24.9 | 22.1 | 2.8 | 39.124 |
| TRMT1L | 5 | 5 | 0 | 5 | 5 | 0 | 13.3 | 13.3 | 0 | 65.033 |
| IPO9 | 6 | 7 | 2 | 6 | 7 | 2 | 8.4 | 9.3 | 1.7 | 115.96 |
| ZRANB2 | 3 | 4 | 0 | 3 | 4 | 0 | 9.4 | 13 | 0 | 37.404 |
| SLC25A10 | 5 | 4 | 0 | 5 | 4 | 0 | 19.5 | 17 | 0 | 48.099 |
| PTGES3 | 3 | 3 | 0 | 3 | 3 | 0 | 21.3 | 21.3 | 0 | 19.155 |
| NOM1 | 7 | 7 | 0 | 7 | 7 | 0 | 10.1 | 10.1 | 0 | 96.256 |
| PRPS1 | 5 | 6 | 0 | 5 | 6 | 0 | 22.3 | 27 | 0 | 34.834 |
| ZC3H15 | 5 | 5 | 0 | 5 | 5 | 0 | 13.8 | 13.8 | 0 | 44.89 |
| PELO | 6 | 7 | 0 | 6 | 7 | 0 | 18.2 | 20.3 | 0 | 43.378 |
| PRPF38A | 4 | 4 | 0 | 4 | 4 | 0 | 13.5 | 13.5 | 0 | 37.476 |
| RUVBL1 | 4 | 4 | 0 | 4 | 4 | 0 | 11.6 | 11.6 | 0 | 50.215 |
| RPSA | 5 | 5 | 0 | 5 | 5 | 0 | 28.1 | 28.1 | 0 | 29.404 |
| NPEPPS | 7 | 9 | 0 | 7 | 9 | 0 | 9.6 | 12 | 0 | 103.28 |
| MTHFD1 | 8 | 7 | 0 | 8 | 7 | 0 | 10.2 | 8.8 | 0 | 92.739 |
| TFIP11 | 7 | 8 | 0 | 7 | 8 | 0 | 9.6 | 11 | 0 | 96.793 |
| CCNL1 | 5 | 4 | 1 | 5 | 4 | 1 | 11 | 8.7 | 1.9 | 59.633 |
| P4HA1 | 6 | 4 | 1 | 6 | 4 | 1 | 13.9 | 9.6 | 2.6 | 60.966 |
| SAFB2 | 13 | 14 | 7 | 6 | 7 | 1 | 16.2 | 16.9 | 9.1 | 107.47 |
| SRPRB | 5 | 5 | 0 | 5 | 5 | 0 | 27.7 | 26.9 | 0 | 29.702 |
| RBM28 | 8 | 10 | 1 | 8 | 10 | 1 | 11.5 | 14.8 | 1.2 | 85.737 |
| PAXBP1 | 5 | 6 | 1 | 5 | 6 | 1 | 8.3 | 10 | 1.1 | 104.8 |
| MCM6 | 7 | 7 | 0 | 7 | 7 | 0 | 10.5 | 10.2 | 0 | 92.888 |
| LTV1 | 5 | 5 | 1 | 5 | 5 | 1 | 15.6 | 14.9 | 2.3 | 54.854 |
| MAD2L1 | 5 | 5 | 0 | 5 | 5 | 0 | 28.3 | 28.3 | 0 | 23.51 |
| MMS19 | 5 | 6 | 0 | 5 | 6 | 0 | 6.5 | 7.1 | 0 | 113.29 |
| FAF2 | 5 | 5 | 0 | 5 | 5 | 0 | 18 | 18 | 0 | 52.623 |
| GART | 5 | 6 | 0 | 5 | 6 | 0 | 7.4 | 8.5 | 0 | 107.72 |
| KRR1 | 4 | 4 | 0 | 4 | 4 | 0 | 11.3 | 11.3 | 0 | 43.664 |
| LSM14B | 6 | 6 | 1 | 5 | 5 | 0 | 19.7 | 19.7 | 2.9 | 42.07 |
| LOC84524 | 4 | 4 | 1 | 4 | 4 | 1 | 15.8 | 15.8 | 3.4 | 30.812 |
| CTPS1 | 6 | 7 | 0 | 4 | 5 | 0 | 12.2 | 14.4 | 0 | 63.723 |
| SKP1 | 3 | 3 | 1 | 3 | 3 | 1 | 22.3 | 22.3 | 9.6 | 22.727 |
| UMPS | 4 | 4 | 0 | 4 | 4 | 0 | 11.7 | 11.7 | 0 | 52.221 |
| EHD4 | 4 | 5 | 0 | 3 | 4 | 0 | 8.1 | 10.2 | 0 | 61.155 |
| CWC25 | 4 | 4 | 0 | 4 | 4 | 0 | 9.4 | 9.4 | 0 | 49.647 |
| POLR2E | 3 | 3 | 2 | 3 | 3 | 2 | 21.7 | 21.7 | 12.5 | 21.459 |
| WDR5 | 3 | 4 | 0 | 3 | 4 | 0 | 11.7 | 14.1 | 0 | 36.588 |
| RPP30 | 3 | 4 | 0 | 3 | 4 | 0 | 13.8 | 19.4 | 0 | 29.321 |
| BRD2 | 6 | 6 | 0 | 6 | 6 | 0 | 8 | 8 | 0 | 88.06 |
| DNAJA3 | 3 | 4 | 0 | 3 | 4 | 0 | 10.8 | 14.3 | 0 | 49.583 |
| ISY1 | 3 | 3 | 0 | 3 | 3 | 0 | 16.1 | 16.1 | 0 | 32.992 |
| RFC3 | 5 | 6 | 0 | 5 | 6 | 0 | 16.9 | 21.9 | 0 | 40.556 |
| PNO1 | 5 | 6 | 0 | 5 | 6 | 0 | 26.6 | 33.7 | 0 | 27.924 |
| ZAK | 4 | 5 | 1 | 4 | 5 | 1 | 5.9 | 7.9 | 1.9 | 91.154 |
| IPO8 | 6 | 6 | 0 | 3 | 3 | 0 | 7.8 | 7.8 | 0 | 119.94 |
| AHSA1 | 3 | 3 | 0 | 3 | 3 | 0 | 11.8 | 11.8 | 0 | 38.274 |
| KPNB1 | 7 | 9 | 0 | 7 | 9 | 0 | 11.8 | 17.2 | 0 | 97.183 |
| ZW10 | 4 | 3 | 0 | 4 | 3 | 0 | 5.4 | 3.7 | 0 | 88.839 |
| EIF6 | 4 | 3 | 0 | 4 | 3 | 0 | 22.9 | 17.1 | 0 | 26.599 |
| HELLS | 7 | 9 | 0 | 7 | 9 | 0 | 9.8 | 12.7 | 0 | 93.961 |
| DDX54 | 5 | 6 | 0 | 5 | 6 | 0 | 6.1 | 8.2 | 0 | 98.594 |
| PPAN | 4 | 5 | 1 | 4 | 5 | 1 | 9 | 10.7 | 1.7 | 47.144 |
| CIZ1 | 4 | 5 | 0 | 4 | 5 | 0 | 5.9 | 7.6 | 0 | 91.124 |
| RBBP6 | 6 | 7 | 2 | 6 | 7 | 2 | 4.2 | 4.8 | 1.5 | 197.36 |
| PHB2 | 4 | 3 | 0 | 4 | 3 | 0 | 21.1 | 17 | 0 | 27.437 |
| ARL1 | 3 | 4 | 0 | 3 | 4 | 0 | 24.9 | 33.7 | 0 | 20.417 |
| GPATCH4 | 4 | 6 | 0 | 4 | 6 | 0 | 13.5 | 18.4 | 0 | 50.381 |
| DARS | 6 | 8 | 2 | 6 | 8 | 2 | 12.6 | 18.6 | 6 | 57.136 |
| MRPS27 | 4 | 4 | 0 | 4 | 4 | 0 | 14 | 14 | 0 | 41.329 |
| FYTTD1 | 3 | 3 | 0 | 3 | 3 | 0 | 9.7 | 11.6 | 0 | 35.818 |
| EIF4G2 | 8 | 9 | 1 | 8 | 9 | 1 | 10.4 | 11.5 | 1.1 | 102.36 |
| MKRN1 | 3 | 4 | 0 | 3 | 4 | 0 | 7.5 | 10.8 | 0 | 53.349 |
| KPNA1 | 5 | 5 | 0 | 5 | 5 | 0 | 11.5 | 11.5 | 0 | 60.221 |
| VDAC1 | 4 | 4 | 0 | 4 | 4 | 0 | 16.3 | 16.3 | 0 | 30.772 |
| TECR | 4 | 5 | 0 | 4 | 5 | 0 | 12.3 | 15.6 | 0 | 36.053 |
| AKAP8L | 5 | 7 | 0 | 5 | 7 | 0 | 8.7 | 11.9 | 0 | 68.193 |
| HMMR | 5 | 4 | 1 | 5 | 4 | 1 | 11.6 | 10.1 | 3.3 | 71.197 |
| TRIM27 | 5 | 5 | 0 | 5 | 5 | 0 | 11.1 | 11.1 | 0 | 58.489 |
| NCOA5 | 7 | 7 | 0 | 7 | 7 | 0 | 18.8 | 18.8 | 0 | 65.536 |
| SLIRP | 3 | 3 | 0 | 3 | 3 | 0 | 38 | 38 | 0 | 10.353 |
| KRI1 | 4 | 5 | 0 | 4 | 5 | 0 | 8 | 9.4 | 0 | 83.251 |
| EIF2S2 | 5 | 5 | 0 | 5 | 5 | 0 | 14.4 | 15.3 | 0 | 38.388 |
| KNOP1 | 4 | 5 | 0 | 4 | 5 | 0 | 10.7 | 13.3 | 0 | 51.588 |
| MRPL14 | 4 | 4 | 0 | 4 | 4 | 0 | 28.3 | 28.3 | 0 | 15.947 |
| FAM208B | 7 | 6 | 0 | 7 | 6 | 0 | 3.6 | 3.2 | 0 | 259.71 |
| KIF23 | 7 | 12 | 1 | 7 | 12 | 1 | 11.1 | 17.9 | 2.8 | 92.682 |
| STT3A | 4 | 4 | 0 | 4 | 4 | 0 | 6.1 | 6.1 | 0 | 80.529 |
| CCT7 | 5 | 6 | 0 | 5 | 6 | 0 | 9.6 | 13.6 | 0 | 59.328 |
| EXOSC10 | 6 | 5 | 0 | 6 | 5 | 0 | 7.9 | 6.6 | 0 | 100.83 |
| WDR3 | 5 | 5 | 1 | 5 | 5 | 1 | 5.9 | 5.9 | 1.4 | 106.1 |
| ZNF24 | 5 | 3 | 1 | 4 | 3 | 0 | 15.5 | 10.6 | 2.4 | 42.155 |
| DHX8 | 5 | 6 | 0 | 4 | 5 | 0 | 5.7 | 6.6 | 0 | 132.78 |
| CEBPZ | 7 | 7 | 0 | 7 | 7 | 0 | 7.5 | 7.1 | 0 | 120.97 |
| CD2BP2 | 4 | 3 | 0 | 4 | 3 | 0 | 13.5 | 10.3 | 0 | 37.646 |
| AIMP2 | 4 | 5 | 0 | 4 | 5 | 0 | 20.3 | 23.1 | 0 | 35.448 |
| UCK2 | 3 | 3 | 0 | 3 | 3 | 0 | 13.8 | 14.6 | 0 | 27.408 |
| PRPF4B | 5 | 5 | 0 | 5 | 5 | 0 | 9.5 | 9.5 | 0 | 68.4 |
| CRNKL1 | 6 | 4 | 0 | 6 | 4 | 0 | 8.6 | 6.3 | 0 | 83.138 |
| ZCCHC9 | 4 | 5 | 0 | 4 | 5 | 0 | 16.6 | 20.3 | 0 | 30.477 |
| ACAT1 | 5 | 5 | 0 | 5 | 5 | 0 | 18.5 | 18.5 | 0 | 45.199 |
| WDR6 | 6 | 6 | 0 | 6 | 6 | 0 | 7.7 | 7.7 | 0 | 125 |
| DDB1 | 7 | 6 | 0 | 7 | 6 | 0 | 5.6 | 4.7 | 0 | 126.97 |
| MRPS26 | 4 | 4 | 0 | 4 | 4 | 0 | 18.5 | 18.5 | 0 | 24.211 |
| MRPS22 | 3 | 4 | 0 | 3 | 4 | 0 | 10.7 | 13.4 | 0 | 33.528 |
| CHD4 | 5 | 7 | 0 | 5 | 7 | 0 | 3.1 | 4.3 | 0 | 216.84 |
| SPATS2 | 5 | 4 | 1 | 5 | 4 | 1 | 11.9 | 9.4 | 1.7 | 59.544 |
| HUWE1 | 6 | 7 | 1 | 6 | 7 | 1 | 2.1 | 2.3 | 0.3 | 374.19 |
| NKTR | 3 | 5 | 0 | 3 | 5 | 0 | 3.2 | 4.7 | 0 | 165.67 |
| PDCD11 | 9 | 8 | 0 | 9 | 8 | 0 | 5 | 4.4 | 0 | 208.83 |
| TCERG1 | 4 | 4 | 0 | 4 | 4 | 0 | 4.2 | 4.2 | 0 | 126 |
| DNAAF5 | 4 | 4 | 0 | 4 | 4 | 0 | 5.8 | 5.8 | 0 | 93.52 |
| USP7 | 9 | 9 | 1 | 9 | 9 | 1 | 9.8 | 9.6 | 1 | 117.03 |
| SLC25A22 | 4 | 4 | 0 | 4 | 4 | 0 | 13.9 | 13.9 | 0 | 34.47 |
| RACGAP1 | 6 | 5 | 0 | 6 | 5 | 0 | 11.9 | 9.3 | 0 | 71.026 |
| PDHB | 4 | 3 | 0 | 4 | 3 | 0 | 15 | 10.6 | 0 | 39.233 |
| FAM208A | 4 | 5 | 0 | 4 | 5 | 0 | 2.5 | 3.3 | 0 | 189.03 |
| TNPO3 | 4 | 6 | 1 | 4 | 6 | 1 | 5.4 | 9.8 | 1.1 | 104.23 |
| UBE3C | 4 | 4 | 0 | 4 | 4 | 0 | 5.3 | 5.3 | 0 | 106.52 |
| ARHGEF2 | 5 | 5 | 0 | 5 | 5 | 0 | 5.6 | 5.6 | 0 | 111.49 |
| BMS1 | 3 | 3 | 0 | 3 | 3 | 0 | 3 | 3 | 0 | 145.81 |
| EIF5B | 6 | 11 | 0 | 6 | 11 | 0 | 7.1 | 12.2 | 0 | 138.8 |
| RPN2 | 3 | 3 | 0 | 3 | 3 | 0 | 8.1 | 6.2 | 0 | 69.333 |
| DDX24 | 3 | 3 | 0 | 3 | 3 | 0 | 5.1 | 4.8 | 0 | 86.188 |
| SMG7 | 4 | 4 | 0 | 4 | 4 | 0 | 5.9 | 5.9 | 0 | 89.724 |
| LUZP1 | 6 | 5 | 0 | 6 | 5 | 0 | 7.2 | 5.7 | 0 | 120.27 |
| HNRNPLL | 4 | 4 | 0 | 4 | 4 | 0 | 10.2 | 10.2 | 0 | 56.45 |
| BUD13 | 4 | 4 | 1 | 4 | 4 | 1 | 7.3 | 7.3 | 1.6 | 70.52 |
| REXO4 | 3 | 4 | 0 | 3 | 4 | 0 | 7.1 | 10.9 | 0 | 46.671 |
| LRRC47 | 4 | 4 | 0 | 4 | 4 | 0 | 8.4 | 8.4 | 0 | 63.472 |
| **USP4** | 3 | 5 | 0 | 3 | 5 | 0 | 15.9 | 29.5 | 0 | 24.874 |
| CFAP20 | 3 | 3 | 0 | 3 | 3 | 0 | 13.5 | 13.5 | 0 | 22.774 |
| DNAJC7 | 3 | 4 | 0 | 3 | 4 | 0 | 6.5 | 8.5 | 0 | 56.44 |
| SERPINH1 | 3 | 4 | 0 | 3 | 4 | 0 | 17.9 | 24.4 | 0 | 23.092 |
| MAP7D3 | 3 | 4 | 0 | 3 | 4 | 0 | 3.6 | 5.1 | 0 | 90.131 |
| MRE11A | 4 | 7 | 0 | 4 | 7 | 0 | 7.5 | 16.2 | 0 | 59.724 |
| TCEB1 | 4 | 3 | 0 | 4 | 3 | 0 | 50.6 | 39.3 | 0 | 9.9601 |
| PSMD6 | 3 | 3 | 0 | 3 | 3 | 0 | 8.5 | 8.5 | 0 | 45.531 |
| PSMC2 | 3 | 3 | 0 | 3 | 3 | 0 | 9.5 | 9.5 | 0 | 45.831 |
| CCDC47 | 4 | 4 | 0 | 4 | 4 | 0 | 8.7 | 11.4 | 0 | 55.873 |
| POLRMT | 4 | 5 | 0 | 4 | 5 | 0 | 4.2 | 6.4 | 0 | 121.23 |
| MSH6 | 4 | 4 | 1 | 4 | 4 | 1 | 3.2 | 3.2 | 1.4 | 142.85 |
| GLYR1 | 3 | 3 | 0 | 3 | 3 | 0 | 6.9 | 6.9 | 0 | 57.215 |
| EXOSC5 | 3 | 3 | 0 | 3 | 3 | 0 | 18.7 | 18.7 | 0 | 27.465 |
| METAP2 | 3 | 3 | 0 | 3 | 3 | 0 | 11.1 | 11.1 | 0 | 28.23 |
| GNL3L | 4 | 5 | 0 | 4 | 5 | 0 | 9.1 | 11.4 | 0 | 58.012 |
| NUSAP1 | 4 | 6 | 0 | 4 | 6 | 0 | 9.8 | 14.5 | 0 | 49.438 |
| CKAP2 | 4 | 4 | 0 | 4 | 4 | 0 | 7.3 | 7.3 | 0 | 76.986 |
| AP3B1 | 4 | 5 | 0 | 4 | 5 | 0 | 4.5 | 5.8 | 0 | 121.32 |
| TBL2 | 3 | 4 | 0 | 3 | 4 | 0 | 11.6 | 15.7 | 0 | 35.379 |
| FASN | 8 | 11 | 1 | 8 | 11 | 1 | 3.9 | 5 | 0.5 | 273.42 |
| TOE1 | 3 | 3 | 0 | 3 | 3 | 0 | 8.8 | 8.8 | 0 | 56.547 |
| CDKN2AIP | 4 | 6 | 0 | 4 | 6 | 0 | 8.1 | 12.1 | 0 | 61.124 |
| SRPK2 | 5 | 5 | 1 | 4 | 4 | 0 | 8.8 | 8.8 | 1.5 | 66.413 |
| PPP2R2A | 3 | 3 | 0 | 3 | 3 | 0 | 8.3 | 8.1 | 0 | 51.647 |
| ADCK3 | 3 | 4 | 0 | 3 | 4 | 0 | 9 | 12 | 0 | 42.476 |
| TELO2 | 3 | 3 | 0 | 3 | 3 | 0 | 4.3 | 4.9 | 0 | 91.746 |
| NUMA1 | 4 | 4 | 0 | 4 | 4 | 0 | 2.7 | 2.7 | 0 | 178.08 |
| PDS5A | 5 | 4 | 0 | 5 | 4 | 0 | 5.7 | 4.3 | 0 | 150.83 |
| HEL103 | 4 | 3 | 0 | 4 | 3 | 0 | 6.1 | 4.3 | 0 | 74.605 |
| MKI67 | 4 | 4 | 0 | 4 | 4 | 0 | 1.4 | 1.4 | 0 | 358.69 |
| YARS | 3 | 3 | 0 | 3 | 3 | 0 | 4.9 | 4.9 | 0 | 59.143 |
| KIAA1524 | 3 | 3 | 0 | 3 | 3 | 0 | 3.5 | 5.3 | 0 | 102.18 |
| MYO1B | 3 | 3 | 0 | 3 | 3 | 0 | 4.9 | 4.9 | 0 | 128.48 |
| RBM5 | 4 | 4 | 0 | 4 | 4 | 0 | 5.4 | 5.4 | 0 | 92.153 |
| SLC25A12 | 6 | 6 | 1 | 3 | 3 | 0 | 9.4 | 9.4 | 1.8 | 74.777 |
| TRIM26 | 3 | 3 | 0 | 3 | 3 | 0 | 5.9 | 7.8 | 0 | 62.174 |
| GTF3C4 | 3 | 3 | 0 | 3 | 3 | 0 | 6.6 | 6.6 | 0 | 91.981 |
| SRBD1 | 5 | 5 | 0 | 5 | 5 | 0 | 4.7 | 6.1 | 0 | 111.74 |
| GIGYF2 | 3 | 3 | 0 | 3 | 3 | 0 | 3.2 | 3.2 | 0 | 150.07 |
| HAX1 | 3 | 3 | 0 | 3 | 3 | 0 | 17.3 | 17.3 | 0 | 26.099 |
| ZNF598 | 3 | 4 | 0 | 3 | 4 | 0 | 4.4 | 6.4 | 0 | 98.636 |
| UTP15 | 4 | 4 | 0 | 4 | 4 | 0 | 11.2 | 11 | 0 | 58.414 |
| FARSLA | 3 | 3 | 0 | 3 | 3 | 0 | 6.1 | 6.1 | 0 | 57.563 |
| RPRD1A | 3 | 3 | 0 | 3 | 3 | 0 | 12.2 | 12.2 | 0 | 35.719 |
| CKAP5 | 3 | 3 | 1 | 3 | 3 | 1 | 1.9 | 1.9 | 0.8 | 225.49 |
| FANCI | 3 | 3 | 0 | 3 | 3 | 0 | 2.7 | 2.4 | 0 | 122.83 |

**Table S2: Information of primary antibodies.**

| **Antibody** | **Company** | **Cat no.** | **Source** | **Application** | **Dilution** |
| --- | --- | --- | --- | --- | --- |
| β-actin | Zen-Bio | R380624 | Rabbit | WB | 1:100000 |
| Lgr6 | Zen-Bio | R24486 | Rabbit | IF | 1:100 |
| Lgr6 | Zen-Bio | R24486 | Rabbit | WB | 1:1000 |
| PPARA | Immunoway | YT3835 | Rabbit | WB | 1:1000 |
| PPARA | Immunoway | YT3835 | Rabbit | IF | 1:100 |
| PPARR | Immunoway | YT3836 | Rabbit | WB | 1:1000 |
| PPARD | Immunoway | YM3601 | Mouse | WB | 1:1000 |
| USP4 | Abclonal | A21644 | Rabbit | WB | 1:1000 |
| USP4 | Abclonal | A21644 | Rabbit | IF | 1:100 |
| USP4 | Abcam | ab245654 | Rabbit | IP | 1:50 |
| P-VASP | Abclonal | AP0187 | Rabbit | WB | 1:1000 |
| VASP | Abclonal | A14217 | Rabbit | WB | 1:1000 |
| P-CREB1 | Abclonal | AP0019 | Rabbit | WB | 1:1000 |
| CREB1 | Abclonal | A11898 | Rabbit | WB | 1:1000 |
| CREB1 | CST | 9198 | Rabbit | CHIP | 1:50 |
| MYH7 | Zen-Bio | 671207 | Rabbit | WB | 1:1000 |
| Ubiquitin | Abclonal | A19686 | Rabbit | COIP | 1:1000 |
| Flag-Tag | Abclonal | AE005 | Mouse | WB | 1:3000 |
| Flag-Tag | Abclonal | AE005 | Mouse | COIP | 1:50 |
| HA-Tag | Abclonal | AE008 | Mouse | WB | 1:3000 |
| HA-Tag | Abclonal | AE008 | Mouse | COIP | 1:50 |
| Myc-Tag | Abclonal | AE010 | Mouse | WB | 1:3000 |
| Myc-Tag | Abclonal | AE010 | Mouse | COIP | 1:50 |

**Table S3: Primers in this study.**

| **Gene Name** | **Sequences** | **Application** |
| --- | --- | --- |
| Mouse Anp | ACCTGCTAGACCACCTGGAG | RT-PCR |
|  | CCTTGGCTGTTATCTTCGGTACCGG |  |
| Mouse Bnp | GAGGTCACTCCTATCCTCTGG | RT-PCR |
|  | GCCATTTCCTCCGACTTTTCTC |  |
| Mouse Col1 | ACTGCAACATGGAGACAGGTCAGA | RT-PCR |
|  | ATCGGTCATGCTCTCTCCAAACCA |  |
| Mouse Myh7 | CCGAGTCCCAGGTCAACAA | RT-PCR |
|  | CTTCACGGGCACCCTTGGA |  |
| Mouse Actin | GGCTGTATTCCCCTCCATCG | RT-PCR |
|  | CCAGTTGGTAACAATGCCATGT |  |
| Mouse Hk1 | TCCATCCACACTTCTCCAGAATC | RT-PCR |
|  | GATCCTGGCTCTTAGGCGTTC |  |
| Mouse Hk2 | TCATTGTTGGCACTGGAAGC | RT-PCR |
|  | TTGCCAGGGTTGAGAGAGAG |  |
| Mouse Pfkl | CCATCAGCAACAATGTGCCTGG | RT-PCR |
|  | TGAGGCTGACTGCTTGATGCGA |  |
| Mouse Eno1 | GCTCTGTGACCGAATCCCTG | RT-PCR |
|  | ACCAGTCTTGATCTGCCCAGTG |  |
| Mouse Ldha | CAAGCAGGTGGTGGACAGTG | RT-PCR |
|  | TGGGACACTGAGGAAGACATC |  |
| Mouse Sdha | GCCCATGCCAGGGAAGATTA | RT-PCR |
|  | GCACTGGCTCGATACTTACC |  |
| Mouse Ogdhl | GGCCCCGCAGCACATTCTCC | RT-PCR |
|  | TCCCGGCCGACATACCATA |  |
| Mouse Fh1 | CAGCCCAAGTCATGGGGAAT | RT-PCR |
|  | CGTTCTTGTGTGCGGTCTTG |  |
| Mouse Idh1 | GAGCTCTCTTGGACCGACTT | RT-PCR |
|  | TGCATCTCCACCACAGAACC |  |
| Mouse Cs | CTCAGCCGCCTCCTTTCAA | RT-PCR |
|  | GGACCGAAGGGAGAGAGCTA |  |
| Rat Hk1 | CTTCATCCACACTTCTCCAGAATC | RT-PCR |
|  | TAGGCGATCGAAGGGTCTCC |  |
| Rat Hk2 | GTCTCAGATAGAGAGCGACTGC | RT-PCR |
|  | ACGGTTCTCTCTTATCTTGTCCAC |  |
| Rat Pfkl | GCTGAGGAATGAGAAGTGCAAC | RT-PCR |
|  | CTGTCAAAGGGAGTTGGGCTTC |  |
| Rat Eno1 | TCAAGGACTACCCAGTGGTG | RT-PCR |
|  | CTTTGAGCAGGAGGCAGTTG |  |
| Rat Ldha | GGTGGTTGACAGTGCATACG | RT-PCR |
|  | TGGGACGCTGAGGAAGACATC |  |
| Rat Sdha | GTTGCAGCACAGGGAGGTAT | RT-PCR |
|  | TCAGAGCCTTTCACGGTGTC |  |
| Rat Ogdhl | GCCGGCCAGGGGGTCGTCTAT | RT-PCR |
|  | CTTGTACATGAGCGGCTGCGTGAA |  |
| Rat Rh1 | CAGGTCCTGTTGCCAGGATT | RT-PCR |
|  | TCTGGGCATGGCAGCTTTTA |  |
| Rat Idh1 | GGTGTGAGCGGGGTTATTGA | RT-PCR |
|  | ACTTGACGCCGACGTTGTAT |  |
| Rat Cs | CCTCTGCATGGACTAGCAAAC | RT-PCR |
|  | GTTGGGCCAGGGGTTCTTAG |  |
| Rat Anp | GGGTAGGATTGACAGGATTGG | RT-PCR |
|  | CTCCAGGAGGGTATTCACCAC |  |
| Rat Bnp | TTCCTTAATCTGTCGCCGCTGG | RT-PCR |
|  | CAGCAGCTTCTGCATCGTGGAT |  |
| Rat Β-Actin | GCGACGAGGCCCAGAGCA | RT-PCR |
|  | GTCCCGGCCAGCCAGGTCCAG |  |
| Rat Silgr6 | UUGUCAUAAAAGUGUAUUGUC | SIRNA |
|  | CAAUACACUUUUAUGACAACC |  |
| Rat Siusp4 | AUUAAGUGUUCCUUCAAGGUC | SIRNA |
|  | CCUUGAAGGAACACUUAAUCG |  |
| Rat Sippara | UUCAGAUAAGGGACUUUCCAG | SIRNA |
|  | GGAAAGUCCCUUAUCUGAAGA |  |
| Rat Sicreb1 | AGAAAAGUCUUUUUAAGUCCU | SIRNA |
|  | GACUUAAAAAGACUUUUCUCC |  |
| Usp4 Promotor | GCCGAGATCTGTGTCTTCCC | CHIP-PCR |
|  | GTGAGATGCCTGAGGGTGTC |  |
